# Adipocyte autophagy limits gut inflammation by controlling oxylipin and IL‐10

**DOI:** 10.15252/embj.2022112202

**Published:** 2023-02-16

**Authors:** Felix Clemens Richter, Matthias Friedrich, Nadja Kampschulte, Klara Piletic, Ghada Alsaleh, Ramona Zummach, Julia Hecker, Mathilde Pohin, Nicholas Ilott, Irina Guschina, Sarah Karin Wideman, Errin Johnson, Mariana Borsa, Paula Hahn, Christophe Morriseau, Bruce D Hammock, Henk Simon Schipper, Claire M Edwards, Rudolf Zechner, Britta Siegmund, Carl Weidinger, Nils Helge Schebb, Fiona Powrie, Anna Katharina Simon

**Affiliations:** ^1^ Kennedy Institute of Rheumatology University of Oxford Oxford UK; ^2^ Translational Gastroenterology Unit, Nuffield Department of Medicine, John Radcliffe Hospital University of Oxford Oxford UK; ^3^ Faculty of Mathematics and Natural Sciences University of Wuppertal Wuppertal Germany; ^4^ Max Delbrück Center Berlin Germany; ^5^ Charité – Universitätsmedizin Berlin, Corporate Member of Freie Universität Berlin, Humboldt‐Universität zu Berlin and Berlin Institute of Health Berlin Germany; ^6^ Department of Gastroenterology, Infectious Diseases and Rheumatology Campus Benjamin Franklin Berlin Germany; ^7^ School of Biosciences Cardiff University Cardiff UK; ^8^ MRC Human Immunology Unit, MRC Weatherall Institute of Molecular Medicine, John Radcliffe Hospital University of Oxford Oxford UK; ^9^ The Dunn School of Pathology University of Oxford Oxford UK; ^10^ Department of Entomology and Nematology, UC Davis Comprehensive Cancer Center University of California Davis CA USA; ^11^ Center for Translational Immunology University Medical Center Utrecht Utrecht The Netherlands; ^12^ Nuffield Department of Orthopaedics, Rheumatology and Musculoskeletal Sciences, Botnar Research Centre University of Oxford Oxford UK; ^13^ Nuffield Department of Surgical Sciences, Botnar Research Centre University of Oxford Oxford UK; ^14^ Institute of Molecular Biosciences University of Graz Graz Austria

**Keywords:** adipocyte, autophagy, IL‐10, inflammation, oxylipin, Autophagy & Cell Death

## Abstract

Lipids play a major role in inflammatory diseases by altering inflammatory cell functions, either through their function as energy substrates or as lipid mediators such as oxylipins. Autophagy, a lysosomal degradation pathway that limits inflammation, is known to impact on lipid availability, however, whether this controls inflammation remains unexplored. We found that upon intestinal inflammation visceral adipocytes upregulate autophagy and that adipocyte‐specific loss of the autophagy gene *Atg7* exacerbates inflammation. While autophagy decreased lipolytic release of free fatty acids, loss of the major lipolytic enzyme *Pnpla2/Atgl* in adipocytes did not alter intestinal inflammation, ruling out free fatty acids as anti‐inflammatory energy substrates. Instead, *Atg7*‐deficient adipose tissues exhibited an oxylipin imbalance, driven through an NRF2‐mediated upregulation of *Ephx1*. This shift reduced secretion of IL‐10 from adipose tissues, which was dependent on the cytochrome P450‐EPHX pathway, and lowered circulating levels of IL‐10 to exacerbate intestinal inflammation. These results suggest an underappreciated fat‐gut crosstalk through an autophagy‐dependent regulation of anti‐inflammatory oxylipins via the cytochrome P450‐EPHX pathway, indicating a protective effect of adipose tissues for distant inflammation.

## Graphical abstract

Gut inflammation modulates autophagy‐dependent oxylipin metabolism in distant fat cells to trigger the release of anti‐inflammatory signals.

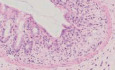

## Introduction

Autophagy is an essential cellular recycling pathway that engulfs cellular contents, including organelles and macromolecules, in a double membraned autophagosome and directs them toward lysosomal degradation. Many cell types, including immune cells, are reliant on autophagy during their differentiation and for their functions (Clarke & Simon, 2019). Consequently, autophagy dysfunction is associated with the development of a variety of inflammatory diseases and metabolic disorders (Deretic, 2021; Klionsky *et al*, 2021). Inflammatory bowel diseases (IBD) including its two predominant manifestations, Crohn's disease (CD) and ulcerative colitis (UC), describe a complex spectrum of intestinal inflammation. Genome‐wide association studies identified autophagy‐related genes as susceptibility alleles in IBD (Hampe *et al*, 2007; McCarroll *et al*, 2008; Jostins *et al*, 2012). Mechanistic studies revealed that ablation of autophagy in immune and epithelial cells promotes intestinal inflammation (Cadwell *et al*, 2008, 2009; Kabat *et al*, 2016). In addition to the strong genetic association of autophagy and IBD, patients with CD often present with an expansion of the mesenteric adipose tissue around the inflamed intestine, indicating an active involvement of the adipose tissue in the disease pathology (Sheehan *et al*, 1992).

Adipose tissues represent an important immunological organ harboring a variety of immune cells, which are highly adapted to live in lipid‐rich environments such as adipose tissue macrophages (ATMs) (Trim & Lynch, 2021). Lean adipose tissues are predominantly populated by tissue‐resident M2‐type ATMs, while inflammation, such as induced by obesity, subverts their homeostatic function and promotes pro‐inflammatory M1‐type polarization (Russo & Lumeng, 2018). Polarization and function of ATMs depend on the integration of a variety of inflammatory and metabolic signals. M2‐type macrophages rely on the availability and uptake of lipids and the subsequent metabolization of free fatty acids (FFA) compared to M1‐type macrophages (Huang *et al*, 2014). In addition, oxygenated polyunsaturated fatty acids, so called oxylipins, which are produced through enzymatic lipid oxidation can be released from adipocytes to modify macrophage cytokine production (Klein‐Wieringa *et al*, 2013). Oxylipins have been widely described as regulatory lipid mediators that regulate inflammatory processes and resolution (Imig & Hammock, 2009; Gilroy *et al*, 2016; Edin *et al*, 2018). It is plausible that, either or both, availability of energy substrates such as FFA and signaling through oxylipin mediators will modulate immune responses. Autophagy contributes to FFA release (Singh *et al*, 2009) and lipid peroxidation (Cai *et al*, 2018), however, to‐date, little is known about the impact of autophagy in adipocytes on these metabolic cues and whether these may affect inflammation.

Here, we sought to investigate the impact of adipocyte autophagy on the immune system during inflammation of a distant organ, the intestine. We observed that autophagy is induced in mature adipocytes upon dextran sulphate sodium (DSS)‐induced intestinal inflammation, and that loss of autophagy in adipocytes exacerbated gut inflammation. Mechanistically, while autophagy in mature adipocytes is required for the optimal release of FFA during inflammation, this was not causative for increased intestinal inflammation. Instead, loss of adipocyte autophagy stabilized the oxidative stress master transcription factor NRF2 and promoted the oxylipin pathway activity shifting the balance of intratissual oxylipins. Local oxylipin imbalance limited the production of anti‐inflammatory IL‐10 from ATMs, aggravating intestinal inflammation. Taken together, we demonstrate a novel mechanism of autophagy in adipocytes regulating local oxylipins that promote the anti‐inflammatory fat‐gut crosstalk, highlighting the importance of intertissual control of inflammation.

## Results

### DSS‐induced intestinal inflammation induces lipolysis and autophagy in adipose tissues

**Figure 1:**
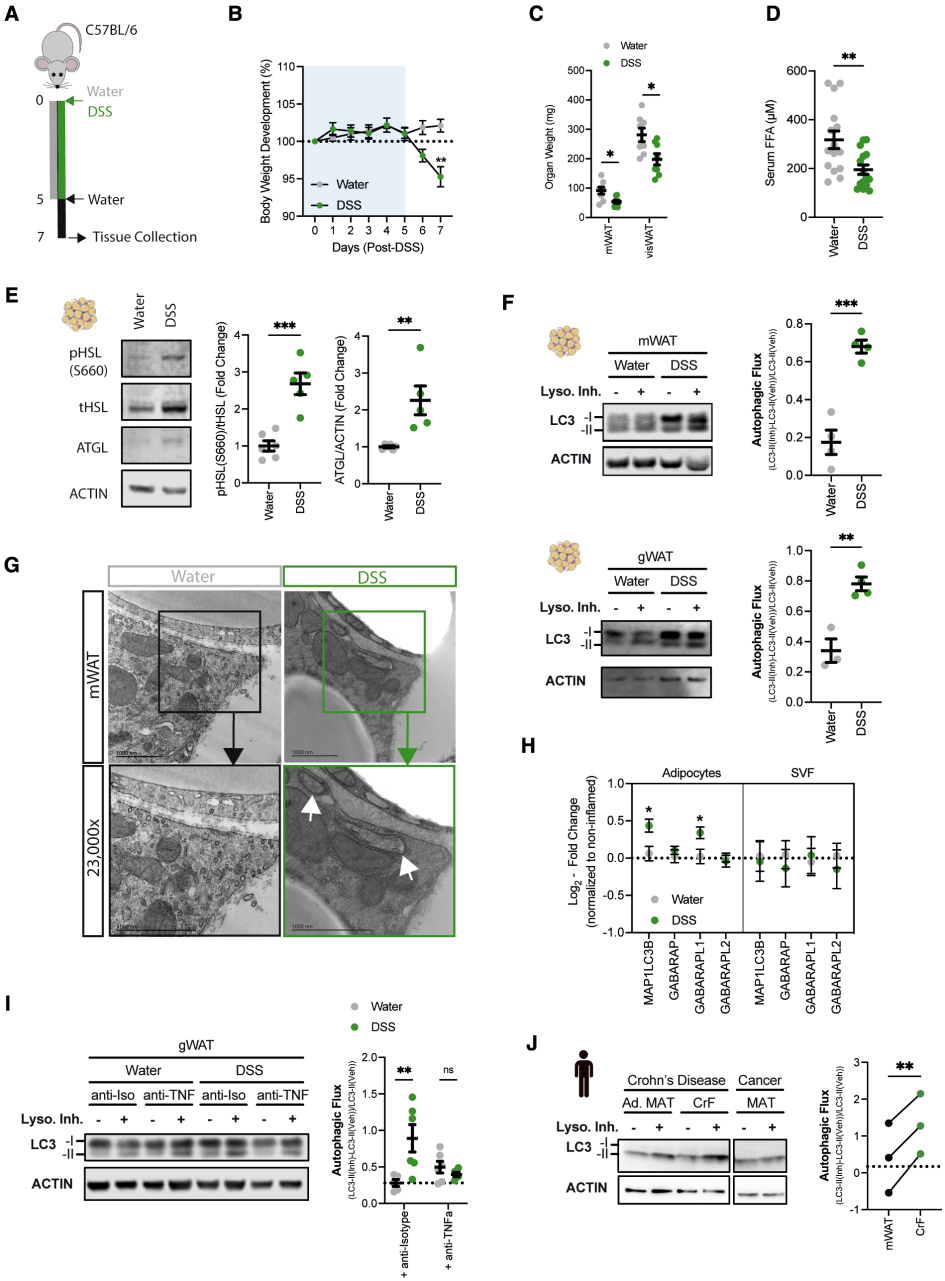
Intestinal inflammation promotes lipolysis and autophagy in adipose tissues Schematic of experimental design. Sex‐matched and age‐matched wild‐type mice were treated for 5 days with 1.5–2% DSS in drinking water, before switched to water for two more days. Mice were sacrificed on day 7 post‐DSS induction.Body weight development upon DSS treatment (*n* = 13/group).Tissue weights measured in mesenteric (mWAT) and collective visceral white adipose tissue (visWAT), consisting of gonadal (gWAT), retroperitoneal and omental white adipose tissue on day 7 after start of DSS regime (*n* = 8/group).Circulating serum levels of FFA during DSS‐induced colitis on day 7 (*n* = 15/group).Representative immunoblot for key lipolytic enzymes HSL and ATGL protein expression and quantification (*n* = 5–6/group).Immunoblot analysis of autophagic flux in mWAT (upper panel) and gWAT (lower panel) adipose tissue stimulated *ex vivo* with lysosomal inhibitors 100 nM Bafilomycin A1 and 20 mM NH_4_Cl for 4 h or DMSO (Vehicle) (*n* = 3–4/group).Representative transmission electron microscopy images from mesenteric adipose tissue 7 days post DSS‐induced colitis induction. Lower panel is showing magnification of selected area. White arrows show autophagosomal structures.
*Atg8* homologs expression was measured by qPCR in visceral adipocytes fraction (left panel) and stromal vascular fraction (right panel) during DSS‐induced colitis (*n* = 7–8/group).Representative immunoblot for LC3‐I/‐II protein expression and quantification of autophagic flux in gWAT via *ex vivo* lysosomal inhibition using 100 nM Bafilomycin A1 and 20 mM NH_4_Cl for 4 h or DMSO (Vehicle). Mice were initially treated with 500 μg anti‐TNFα antibody or isotype control, before administrating DSS in drinking water for 5 days. Mice were sacrificed on day 7 post‐DSS induction (*n* = 5–6/group).Representative immunoblot for LC3‐I/‐II and ACTIN protein expression and quantification of autophagic flux in creeping fat tissues (CrF) and adjacent mesenteric adipose tissues (Ad. MAT) of Crohn's disease patients (*n* = 3/group). Additionally, autophagic flux was determined in the mesenteric adipose tissue (MAT) of a colorectal cancer patient as control (dotted line). Data are represented as mean ± s.e.m. (B) Two‐Way repeated measures ANOVA. (C–E, G) Unpaired Student's *t*‐test. (I) Two‐Way ANOVA. (J) Paired Student's *t*‐test. *P < 0.05, **P < 0.01, ***P < 0.001. Source data are available online for this figure.

**Figure EV1:**
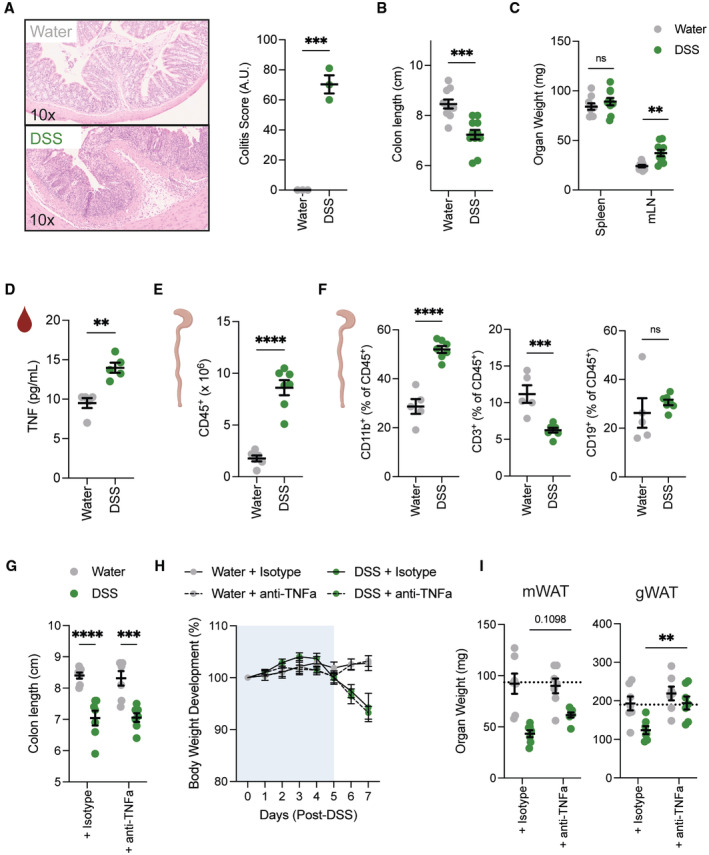
DSS leads to efficient induction of intestinal inflammation Representative H&E staining of colon histology and quantification on day 7 after DSS colitis induction (*n* = 3/group) from one independent experiment.Colon length measured after 1.5–2% DSS colitis regime on day 7 (*n* = 10–11/group).Spleen weight and mesenteric lymph node weight after 1.5–2% colitis regime on day 7 (*n* = 9–10/group).TNFα levels in serum were measured in wild‐type mice on day 7 after water and DSS treatment (*n* = 5/group).Absolute number of colonic CD45^+^ immune cells on day 7 post‐DSS treatment (*n* = 6–7/group).Frequency of CD11b^+^ myeloid cells, CD3^+^ T cells and CD19^+^ B cells in colon on day 7 post‐DSS treatment (*n* = 5–7/group).Colon length of mice upon DSS‐induced colitis treated with anti‐isotype or anti‐TNFα neutralizing antibody (*n* = 7/group).Body weight development upon DSS‐induced colitis of mice treated either with anti‐isotype or anti‐TNFα neutralizing antibody (*n* = 7/group).Tissue weight of mWAT or gWAT upon DSS‐induced colitis of mice treated either with anti‐isotype or anti‐TNFα neutralizing antibody (*n* = 7/group). Data are represented as mean ± s.e.m. (A–E) Unpaired Student's *t*‐test. (G, I) Two‐way ANOVA. (H) Repeat‐measure two‐way ANOVA. **P < 0.01, ***P < 0.001, ****P < 0.0001. Source data are available online for this figure.

To investigate how the adipose tissue is affected by intestinal inflammation, we deployed a mouse model of intestinal inflammation evoked by the administration of DSS in drinking water (Fig 1A). As expected, treatment with DSS led to an increased histopathological inflammation, shortened colon length, enlarged mesenteric lymph nodes, and elevated circulating levels of the pro‐inflammatory cytokine TNFα (Fig EV1A–D). In addition, DSS treatment resulted in a significantly higher infiltration of immune cells in the inflamed colon, predominantly of myeloid origin (Fig EV1E and F). Furthermore, DSS colitis reduced body weight (Fig 1B), and in line with that, visceral adipose tissue mass (Fig 1C), as well as serum FFA levels (Fig 1D). Next, we examined whether DSS‐induced colitis can lead to the activation of key lipolysis enzymes in the adipose tissue, contributing to the decrease in adipose tissue mass. We confirmed that DSS‐induced colitis increased phosphorylation of hormone‐sensitive lipase (HSL) and the expression of adipose triglyceride lipase (ATGL) (Fig 1E), in line with increased lipolytic activity from the adipocytes.

Lipids can also be provided via the classical lipolysis or through degradation of the lipid droplet via autophagy. Thus, we assessed the autophagy levels in adipose tissue explants from water‐ or DSS‐treated animals, which were cultured in the absence or presence of lysosomal inhibitors and the accumulation of the lipidated autophagosomal marker LC3 protein (LC3‐II) was quantified. DSS‐induced intestinal inflammation substantially increased autophagic flux in mesenteric and in gonadal white adipose tissue (mWAT and gWAT, respectively) (Fig 1F), indicating that both adipose tissues proximal and distal to the intestine are responsive to the inflammation. To validate that adipocytes, but not other adipose tissue‐resident cell types, contribute to the increased autophagic flux in the adipose tissue, we first prepared adipose tissues for transmission electron microscopy. Autophagosomal double‐membrane structures were readily identified in adipocytes from DSS‐treated mice (Fig 1G). Additionally, enriched adipocytes, but not vascular stromal fractions, increased the expression of several *Atg8* homologs upon DSS colitis, further demonstrating an induction of autophagy in this cell type (Fig 1H).

TNFα has previously been shown to be a potent inducer of autophagy in *in vitro* differentiated 3T3‐L1 cells (Ju *et al*, 2019), prompting the hypothesis that the release of TNFα during DSS‐induced intestinal inflammation augments autophagic flux in the adipose tissue. To test this, we blocked TNFα *in vivo* using a neutralizing antibody. Mice treated with anti‐TNFα and anti‐Isotype showed similar body weight loss and colon shortening during DSS‐induced inflammation, indicating that the mice were similarly inflamed (Fig EV1G and H). In contrast, loss of adipose tissue mass was partially prevented by neutralization of TNFα (Fig EV1I), possibly indicating a reduced release of lipids from the adipose tissue. Importantly, we found that adipose tissue autophagic flux was reduced upon anti‐TNFα treatment (Fig 1I).

Lastly, we wanted to assess whether the increase in autophagic flux also occurs in IBD. For this purpose, we collected creeping fat tissues and adjacent noninflamed mesenteric adipose tissues from CD patients. Interestingly, similar to the DSS‐induced mice, we found an increased autophagic flux in the creeping fat compared to the same patient's adjacent noninflamed mesenteric adipose tissue (Fig 1J).

Overall, these results demonstrate that lipolysis and autophagy are induced in adipocytes in response to DSS‐induced intestinal inflammation.

### Loss of adipocyte autophagy exacerbates intestinal inflammation

**Figure 2:**
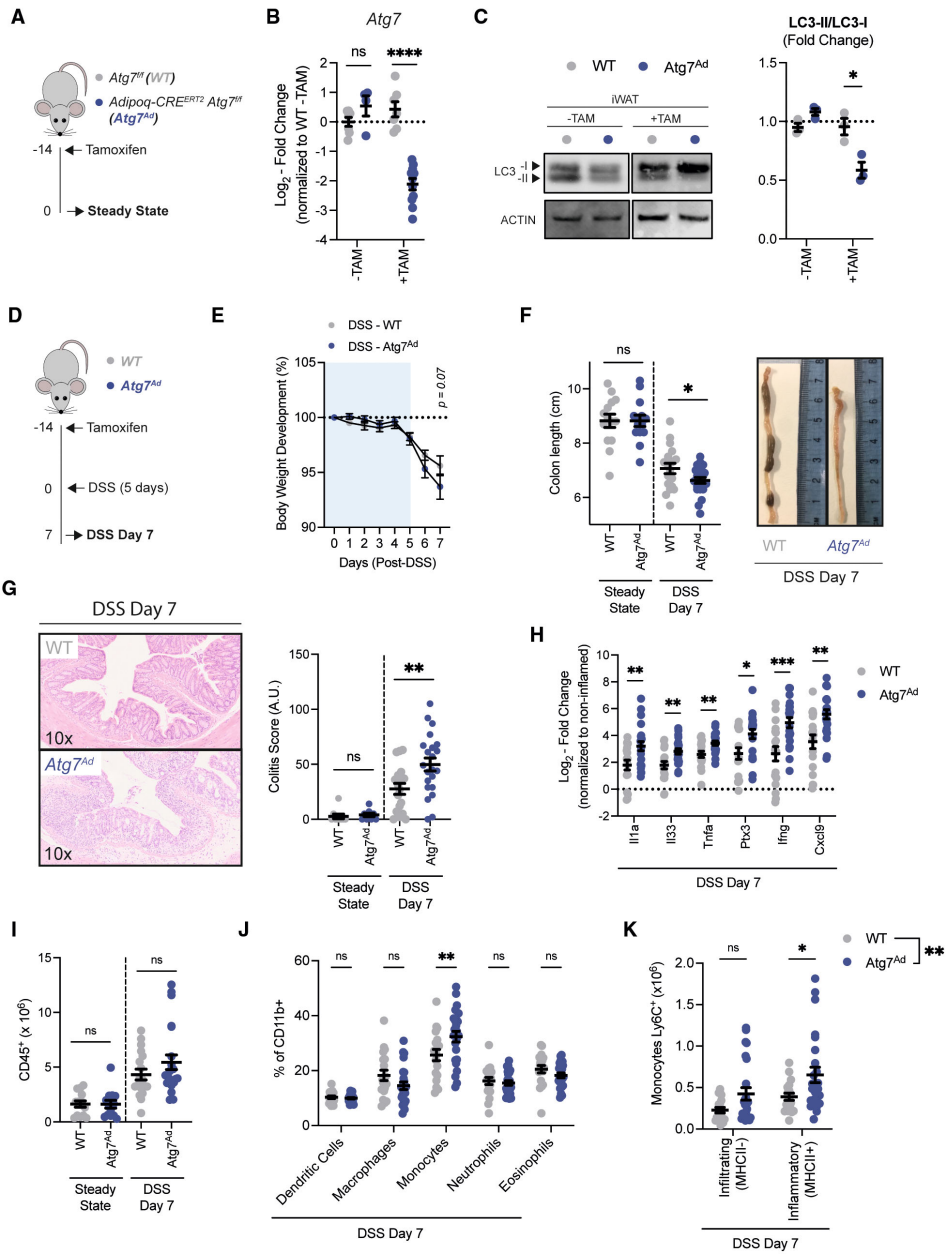
Loss of adipocyte autophagy exacerbates DSS‐induced colitis Schematic of experimental design. Sex‐matched and age‐matched littermates were treated at 8–12 weeks of age with tamoxifen for 5 consecutive days before tissues were analyzed 14 days after the last tamoxifen administration (Steady State).Representative quantification of knock‐out efficiency measured on *Atg7* transcript level by qRT‐PCR in purified primary visceral adipocyte at 2 weeks post‐tamoxifen treatment (*n* = 4–11/group).Representative immunoblot for LC3‐I and LC3‐II protein expression and quantification of LC3 conversion ratio (LC3‐II/LC3‐I) (*n* = 3/group).Schematic of experimental design. Sex‐matched and age‐matched littermates were treated at 8–12 weeks of age with tamoxifen for 5 consecutive days and DSS‐induced colitis was induced after a 2‐week washout phase (DSS Day 7).Body weight development upon DSS treatment (*n* = 25/group).Colon length after 2 weeks postdeletion (steady state; *n* = 14/group) and after DSS on day 7 (*n* = 18–22/group).Representative H&E staining images (10× magnification) of colon sections and quantification of histological score at steady state (*n* = 9/group) and DSS colitis (*n* = 18–22/group).Expression of pro‐inflammatory cytokines in colon tissues at 7 days post‐DSS induction (*n* = 18–22/group). Dotted line represents uninflamed controls.Absolute number CD45^+^ immune cells from colons at steady state (*n* = 13–14/group) or at 7 days post‐DSS induction (*n* = 18–22/group).Frequency of myeloid cell population in colon on day 7 post‐DSS induction (*n* = 18–25/group).Absolute number of Ly6C^+^ monocytes discriminated by the absence or presence of MHCII for infiltrating and inflammatory monocytes, respectively (*n* = 18–25/group). Data are represented as mean ± s.e.m. (B, C, F–I) Unpaired Student's *t*‐test. (E) Two‐Way repeated measures ANOVA. (J, K) Two‐Way ANOVA. *P < 0.05, **P < 0.01, ***P < 0.001, ****P < 0.0001. Source data are available online for this figure.

Given the increased adipose autophagy we observed as a reaction to intestinal inflammation, we next investigated whether loss of autophagy in adipocytes affects intestinal inflammation. To exclude developmental effects of autophagy loss (Singh *et al*, 2009; Zhang *et al*, 2009), we used a tamoxifen‐inducible knockout mouse model to ablate the essential autophagy gene *Atg7* specifically in mature adipocytes (*Atg7* ^*Ad*^) in adult mice (Fig 2A). Tamoxifen administration led to the significant reduction of *Atg7* transcript levels in visceral adipocytes (Fig 2B). Importantly, the adipocyte‐specific loss of *Atg7* resulted in the interruption of conversion of LC3‐I to LC3‐II in the adipose tissue (Fig 2C), confirming effective disruption of the autophagic process in adipose tissue.

Having confirmed efficient deletion of *Atg7* and disruption of autophagic flux in adipocytes, we next compared the effects of autophagy loss in adipocytes in steady‐state and DSS‐induced colitis. As tamoxifen is known to induce autophagy, and we found this to be true in this setting, we included tamoxifen treatment for all genotypes and added a 2‐week wash‐out period before treatment with DSS (Fig 2D). In all assessed parameters, loss of adipocyte autophagy in steady‐state mice had no effects on intestinal immune homeostasis. In contrast, *Atg7* ^*Ad*^ mice showed an increased loss of body weight in comparison to littermate controls upon DSS‐treatment (Fig 2E). In addition, *Atg7* ^*Ad*^ mice treated with DSS had significantly shorter colon when compared to their wild‐type littermates during acute inflammation (Fig 2F). Blinded histopathological assessment (Dieleman *et al*, 1998) confirmed that DSS‐treated *Atg7* ^*Ad*^ mice exhibited more severe tissue damage accompanied by increased inflammation and reduced features of repair throughout the colon (Fig 2G). Consistent with an increased inflammatory response, we found increased gene expression of alarmins such as *Il1a* and *Il33*, pro‐inflammatory cytokines *Tnfa*, *Ptx3*, *Ifng*, and the IFNγ‐regulated chemokine *Cxcl9* in *Atg7* ^*Ad*^ mice (Fig 2H). Although total CD45^+^ immune cell numbers were comparable between adipocyte autophagy‐deficient mice and littermate controls (Fig 2I), DSS‐inflamed *Atg7* ^*Ad*^ mice showed an increased frequency of monocytes infiltrating the intestinal tissue (Fig 2J). In particular, the number of MHCII‐expressing, inflammatory monocytes were increased in the lamina propria of *Atg7* ^*Ad*^ mice (Fig 2K). This phenotype is in line with the fact that autophagic flux was induced in adipose tissues upon intestinal damage by DSS (Fig 1F) suggesting an important function of adipocyte autophagy during intestinal inflammation. Taken together, these data demonstrate that loss of adipocyte autophagy exacerbates intestinal inflammation in the acute phase of DSS‐induced colitis.

**Figure EV2:**
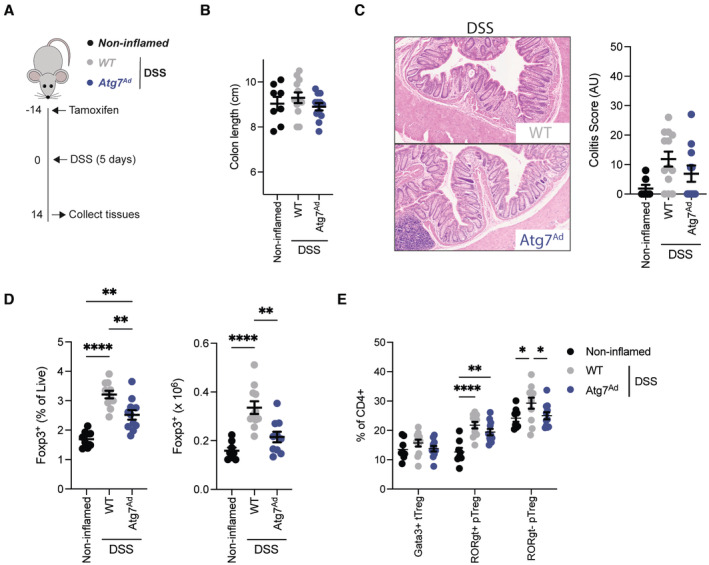
Expansion of intestinal Treg populations is blunted in adipocyte autophagy‐deficient mice without affecting intestinal resolution Schematic of experimental design. Sex‐matched and age‐matched littermates were treated with DSS for 5 days and mice were sacrificed 14 days after start of DSS treatment.Colon length from noninflamed control mice (*n* = 8/group), adipocyte autophagy‐sufficient WT mice and adipocyte autophagy‐deficient mice (*n* = 12/group).Representative H&E staining images (10× magnification) of distal colon sections and quantification of histopathological score (*n* = 7–13/group).Frequency (left panel) and absolute number (right panel) of CD4^+^ FOXP3^+^ cells in the colon on day 14 post‐DSS treatment (*n* = 8–11/group).Frequency of peripheral and thymic Treg (pTreg and tTreg, respectively) cell populations in colon on day 14 post‐DSS treatment (*n* = 8–11/group).Data are represented as mean ± s.e.m. (B–D) One‐way ANOVA. (E) Two‐way ANOVA. *P < 0.05, **P < 0.01, ****P < 0.0001. Source data are available online for this figure.

Since intestinal inflammation induced by DSS is self‐resolving, we assessed the impact of adipocyte autophagy loss during resolution of the inflammation (Fig EV2A). Two weeks after initial DSS administration, we did not find any differences in colon length between *Atg7* ^*Ad*^ and littermate controls, and equally, there were no significant histopathological differences observed between the groups (Fig EV2B and C). Interestingly, frequencies and total numbers of colonic FOXP3^+^ regulatory T cells (Tregs) were decreased in adipocyte autophagy‐deficient animals compared to wild‐type animals (Fig EV2D), despite not affecting disease recovery. Intestinal FOXP3^+^ Tregs are classified into three distinct subsets based on co‐expression of TH_2_ and TH_17_ transcription factors GATA3^+^ and RORgt^+^, respectively (Whibley *et al*, 2019). While all populations tended to be diminished in *Atg7* ^*Ad*^ mice, only RORgt^−^ FOXP3^+^ Tregs were significantly reduced (Fig EV2E). These data suggest that adipocyte autophagy is dispensable for the resolution of DSS‐induced inflammation but may affect expansion of intestinal Tregs in response to intestinal tissue injury.

**Figure 3:**
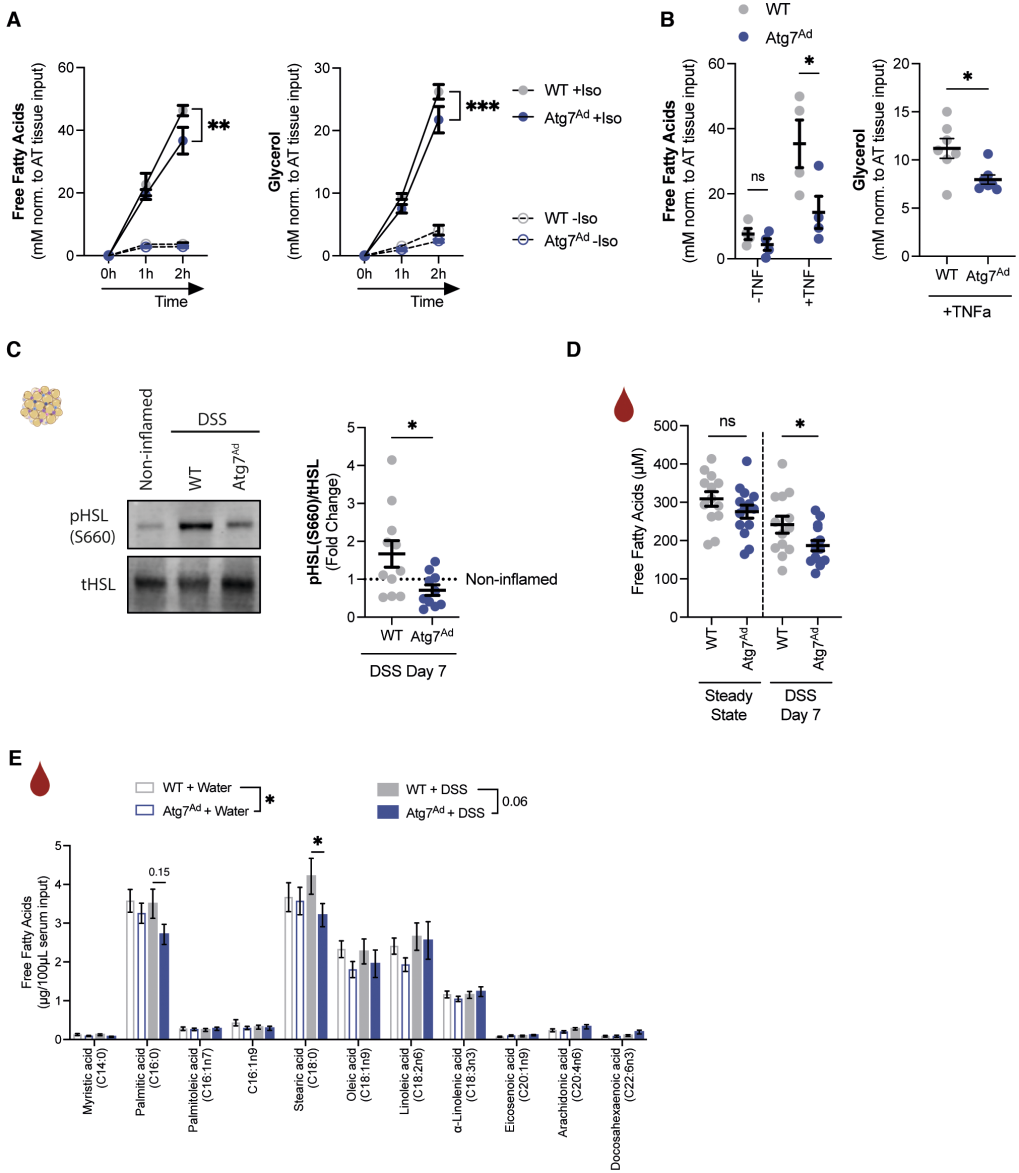
Autophagy loss reduces secretion of fatty acids from adipocytes *Ex vivo* lipolysis measured by released free fatty acid (left, *n* = 4–5/group) and glycerol (right, *n* = 7–8/group) in culture supernatant of adipose tissue explants simulated with isoproterenol (10 μM) for 1–2 h.
*Ex vivo* lipolysis measured by released free fatty acid (left, *n* = 4/group) and glycerol (right, *n* = 7/group) adipose tissue explants simulated with TNFα (100 ng/ml) for 24 h before replacing with fresh medium in the absence of TNFα for 3 h.Representative immunoblot for key lipolytic enzymes HSL, pHSL (Ser660) and quantification (*n* = 10–11/group).Serum levels of circulating FFAs measured in wild‐type and *Atg7*‐deficient mice (*n* = 13–14/group).Concentration of individual FFA species in serum in water‐treated and DSS‐treated mice as measured by FID‐GC (*n* = 12–14/group). Data are represented as mean ± s.e.m. (A, B, E) Two‐Way ANOVA. (B, D) Unpaired Student's *t*‐test. (C) Mann–Whitney test. *P < 0.05, **P < 0.01, ***P < 0.001. Source data are available online for this figure.

### Adipocyte autophagy promotes FFA secretion

**Figure EV3:**
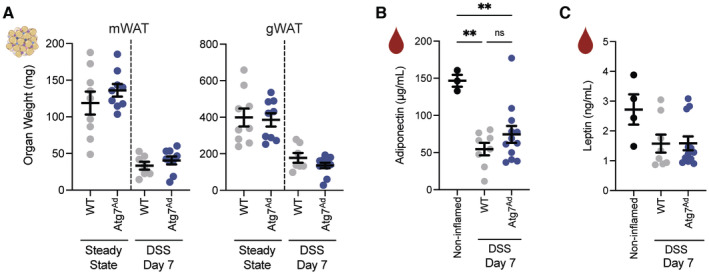
Loss of adipocyte autophagy had no effects on adipose tissue and circulating levels of leptin and adiponectin Adipose tissue mass at steady state and on day 7 post‐DSS induction (*n* = 7–11/group).Circulating levels of adiponectin (*n* = 3–12/group).Circulating levels of leptin (*n* = 4–12/group).Data are represented as mean ± s.e.m. (A) Unpaired Student's *t*‐test. (B, C) One‐way ANOVA. **P < 0.01. Source data are available online for this figure.

Recent reports implicated autophagy in mature adipocytes in the secretion of FFA in response to β‐adrenergic receptor‐mediated lipolysis (Cai *et al*, 2018; Son *et al*, 2020). To confirm the importance of adipocyte autophagy for optimal lipolytic output, adipose tissue explants were stimulated with the β‐adrenergic receptor agonist isoproterenol and FFA levels were quantified. As expected, FFA and glycerol release was reduced upon lipolysis stimulation in autophagy‐deficient as compared to autophagy‐proficient adipocytes (Fig 3A). TNFα is a crucial cytokine for human and murine IBD pathologies (Friedrich *et al*, 2019) and it can affect adipose tissue through inhibition of lipogenesis and by promoting FFA secretion (Cawthorn & Sethi, 2008). Since circulating TNFα levels were elevated during DSS colitis (Fig EV1D), we investigated its effects on adipocyte lipid metabolism. In the presence of TNFα, FFA and glycerol release was significantly blunted in autophagy‐deficient compared to wild‐type adipocytes (Fig 3B). Next, the impact of adipocyte autophagy loss on the induction of lipolysis in the context of DSS‐induced colitis was assessed. Induction of HSL phosphorylation was reduced in adipose tissues of *Atg7* ^*Ad*^ mice, suggesting a reduced lipolytic potential of autophagy‐deficient adipocytes (Fig 3C). Consistent with the decreased lipolytic activity of autophagy‐deficient adipocytes, *Atg7* ^*Ad*^ mice exhibit reduced serum FFA levels compared to wild‐type littermates upon DSS colitis (Fig 3D). While we established that autophagy could modulate overall FFA release, we next tested whether autophagy affects the production and secretion of specific FFA species. To investigate this, serum samples from water‐ and DSS‐treated animals were analyzed by GC‐FID. Confirming our initial findings, the serum concentration of many FFA species was reduced upon adipocyte autophagy loss, indicating that adipocyte autophagy controls overall FFA levels rather than specific FFAs (Fig 3E). Interestingly, loss of adipose tissue mass was comparable between both genotypes upon DSS‐induced colitis (Fig EV3A).

**Figure 4:**
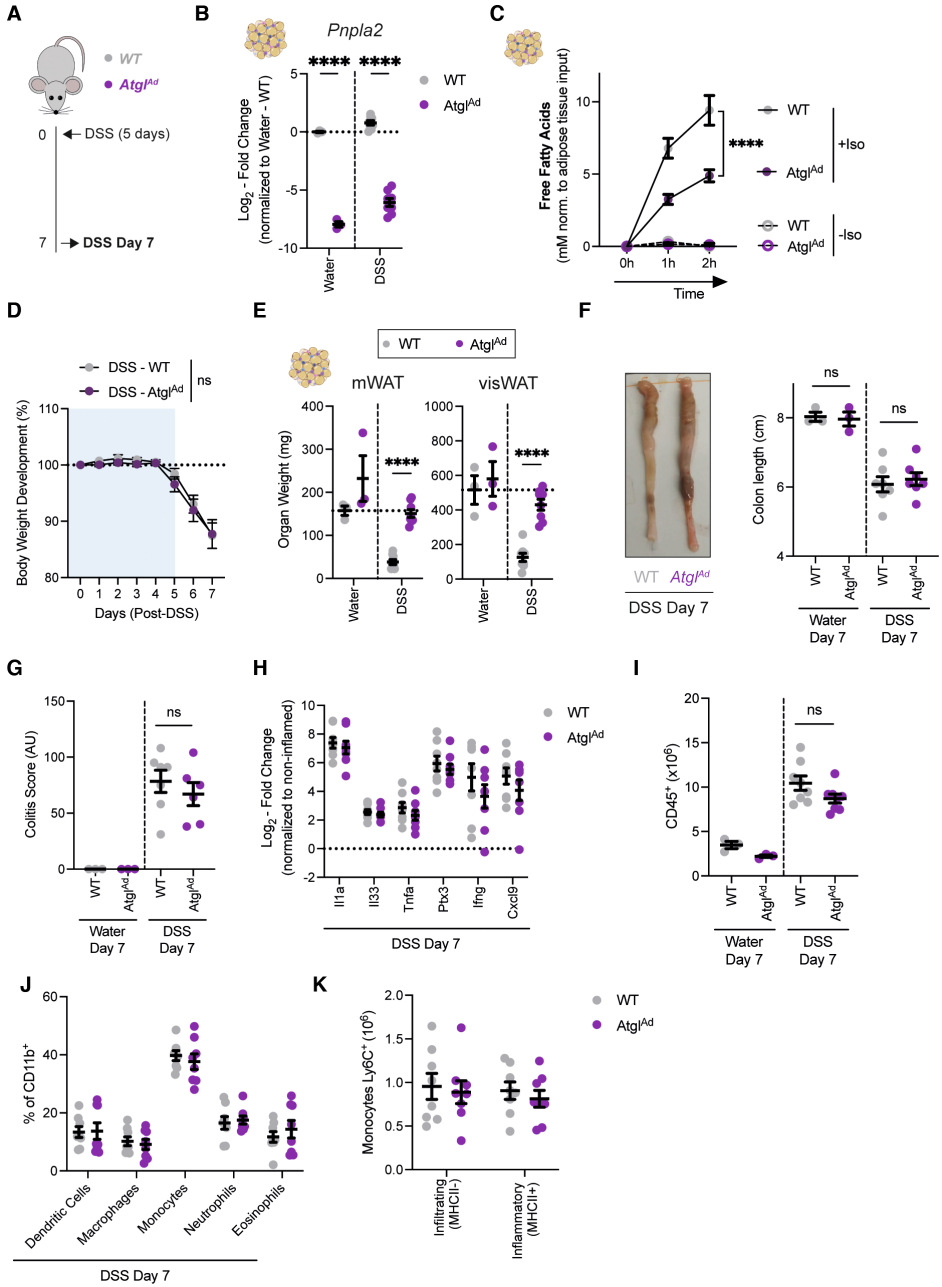
Adipocyte‐specific loss of *Atgl* was dispensable for regulation of intestinal inflammation Schematic of experimental design. DSS‐induced colitis was induced in sex‐matched and age‐matched littermates.Representative quantification of knockout efficiency measured on *Atgl* transcript level by qRT‐PCR in purified primary visceral adipocyte (*n* = 3–8/group).
*Ex vivo* lipolysis assays on *Atg7*‐deficient adipose tissue explants simulated with isoproterenol (10 μM) for 1–2 h (*n* = 5–6/group).Body weight development upon DSS treatment (*n* = 8/group).Tissue weights of mWAT and visWAT on day 7 after start of DSS (*n* = 3–8/group).Colon length after DSS on day 7 (*n* = 3–8/group).Quantification of histological score at steady state (*n* = 3/group) and DSS colitis (*n* = 6–7/group).Expression of pro‐inflammatory cytokines in colon tissues on 7 days post‐DSS induction (*n* = 8/group). Dotted line represents noninflamed controls.Absolute number CD45^+^ immune cells from colons on 7 days post‐DSS induction (*n* = 3–8/group).Frequency of myeloid cell population in colon on day 7 post‐DSS induction (*n* = 8/group).Absolute number of Ly6C^+^ monocytes discriminated by the absence or presence of MHCII for infiltrating and inflammatory monocytes, respectively (*n* = 8/group). Data are represented as mean ± s.e.m. (B, E–I, K) Unpaired Student's *t*‐test. (D) Two‐Way repeated measures ANOVA. (C, J) Two‐Way ANOVA. ****P < 0.0001. Source data are available online for this figure.

It has previously been described that the adipokines leptin and adiponectin can influence intestinal inflammation in both preclinical and clinical situations (Siegmund *et al*, 2002; Weidinger *et al*, 2018), we, therefore, assessed the impact of adipocyte autophagy loss on circulating levels of these adipokines. The levels of both adipokines were equally reduced in their circulation, paralleling the general loss of adipose tissue mass (Fig EV3B and C). Taken together, our data suggest that adipocyte autophagy fine‐tunes the lipolytic output in an inflammatory setting.

### Adipocyte lipolysis is dispensable for DSS‐induced colitis severity

Based on our data, we hypothesized that differences in FFA availability may be responsible for a differential intestinal immune response. We, therefore, sought to determine the importance of adipocyte lipolysis during DSS‐induced colitis (Fig 4A) by deleting the cytoplasmic lipase *Pnpla2/Atgl*, a rate‐limiting enzyme in the lipolytic pathway (Schweiger *et al*, 2006). Using adipocyte‐specific *Pnpla2/Atgl* (*Atgl* ^*Ad*^) knockout mice, we first confirmed that *Pnpla2/Atgl* was efficiently deleted in purified visceral adipocytes (Fig 4B), leading to a strong reduction of isoproterenol‐induced FFA release (Fig 4C). Strikingly, upon DSS‐induced colitis, *Atgl* ^*Ad*^ mice lost comparable amounts of body weight (Fig 4D), although adipose tissue loss was completely prevented (Fig 4E). These data underline that *Atgl*‐driven lipolysis is a main driver for adipose tissue loss during DSS‐induced colitis. However, detailed analysis of the colon showed no changes in colon shortening, histopathological scores, and expression of inflammatory cytokines (Fig 4F–H). Similarly, there was no difference in the infiltration and presence of different pro‐inflammatory immune cell population in the colonic lamina propria (Fig 4I–K). In summary, inhibition of bulk FFA release from adipocytes through *Atgl* loss does not mimic the effects on intestinal inflammation observed in *Atg7* ^*Ad*^ mice. This suggests that provision of FFA is unlikely to be the mechanism by which autophagy in adipocytes exerts its anti‐inflammatory role.

### Intestinal inflammation promotes a lipolytic transcriptional profile in primary adipocytes

**Figure EV4:**
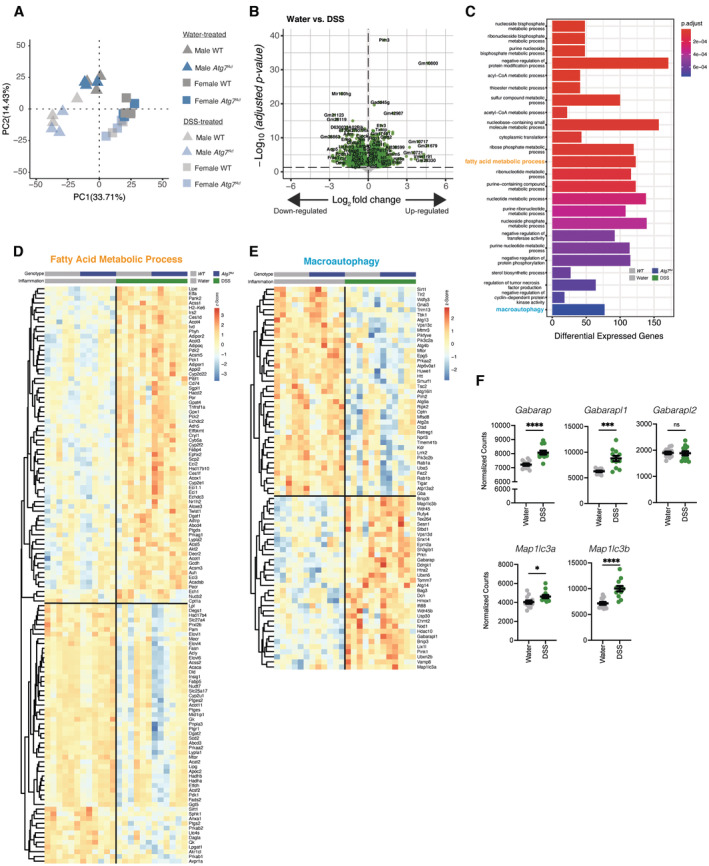
Intestinal inflammation induces distinct transcriptional programs in primary visceral adipocytes Principal component analysis of all mice revealing a strong sex effect in the overall transcriptome.Differential gene expression assessing transcriptional changes associated with DSS‐induced inflammation after regressing effect of sex and genotypes in visceral adipocytes.Pathway enrichment analysis of significantly differentially expressed genes in visceral adipocytes during DSS colitis.Heatmap representing differentially expressed genes associated in fatty acid metabolism during DSS‐induced colitis in visceral adipocytes.Heatmap representing differentially expressed genes associated with macroautophagy during DSS‐induced colitis in visceral adipocytes.Normalized counts of *Atg8* homologs in visceral adipocytes (*n* = 12/group). Data are represented as mean ± s.e.m. (F) Unpaired Student's *t*‐test. *P < 0.05, ***P < 0.001 Source data are available online for this figure.

At this point, it remained unclear how adipocytes regulate intestinal inflammation. We hypothesized that visceral adipocytes would alter their transcriptional inflammatory profile during intestinal inflammation. To test this, visceral adipocytes were collected from wild‐type and *Atg7* ^*Ad*^ mice treated with water or DSS and subjected to RNA sequencing. Since we anticipated sex‐specific differences in adipocyte transcription profiles, we included the same number of male and female mice in each experimental group. The treatment clearly separated the experimental groups in the principal component analysis (PCA) (Fig EV4A). As expected, sex‐specific transcriptional changes explained ~33% of the dataset variance (Fig EV4A), in line with previous reports (Oliva *et al*, 2020). Next, we compared noninflamed to inflamed adipocytes by regressing genotype and sex to identify the impact of intestinal inflammation on the adipocyte transcriptome. More than 4,700 genes were differentially regulated between these states (Fig EV4B), among which 2,415 were significantly upregulated and 2,333 downregulated. Gene ontology analysis of these differentially expressed genes revealed an enrichment in several pathways (Fig EV4C). Confirming our earlier results that adipocyte autophagy is affected by DSS‐induced colitis (Fig 1H), intestinal inflammation led to an enrichment of genes involved in macroautophagy in visceral adipocytes, including an increased expression of several *Atg8* homologs (*Gabarap*, *Gabarapl1*, *Map1lc3a*, *Map1lc3b*) (Fig EV4E and F). In addition, genes related to fatty acid metabolism were enriched in visceral adipocytes during intestinal inflammation (Fig EV4D). Similar to cachexic conditions, an increase in lipolytic genes (*Lipe*, *Pnpla2*) and simultaneous decrease in lipogenic genes (*Dgat2*, *Mogat2*, *Lpl*) was observed (Baazim *et al*, 2021). Overall, intestinal inflammation leads to a broad transcriptional response in visceral adipocytes, altering autophagy and fatty acid metabolism, which is reminiscent of a cachexic response phenotype (Baazim *et al*, 2021). These findings are in line with our previous observations.

### Adipocyte autophagy loss promotes NRF2‐mediated stress response and alters tissue oxylipin levels

**Figure 5:**
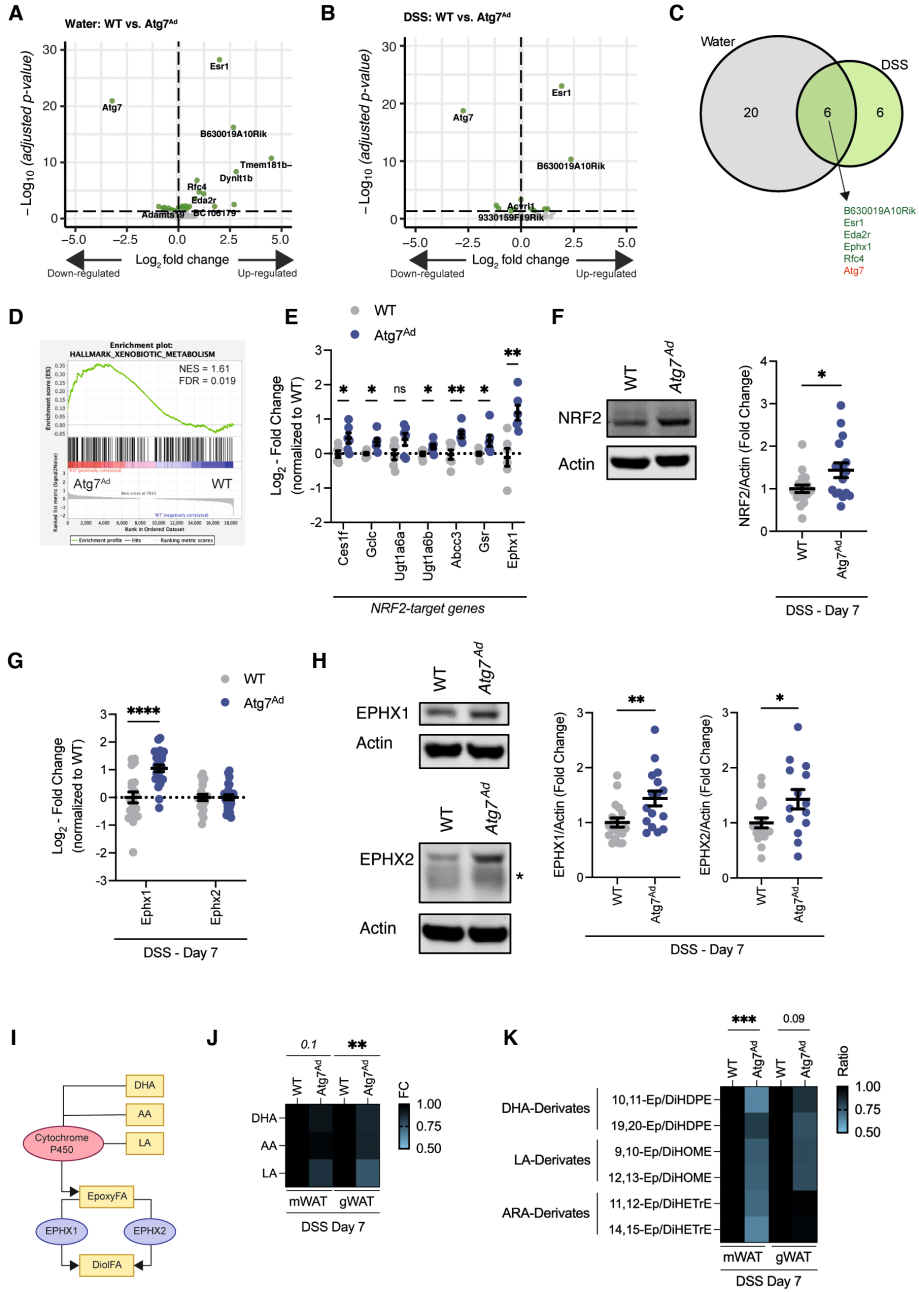
Adipocyte autophagy loss activates NRF2‐EPHX1 pathway and alters intratissual oxylipin balance Differential gene expression in visceral adipocytes from water‐treated WT and *Atg7* ^*Ad*^ animals 2 weeks after tamoxifen treatment.Differential gene expression in visceral adipocytes from DSS‐treated WT and *Atg7* ^*Ad*^ animals on day 7 post‐DSS treatment.Venn diagram of commonly regulated genes between *Atg7*‐deficient and *Atg7*‐sufficient adipocytes during water or DSS treatment.GSEA enrichment analysis between *Atg7*‐deficient and *Atg7*‐sufficient adipocytes during DSS treatment.Fold change expression of NRF2‐target genes in primary visceral adipocytes on day 7 after DSS induction from normalized counts of RNAseq dataset (*n* = 6/group).Representative immunoblot for NRF2 protein expression and quantification (*n* = 16–18/group).Transcriptional expression of *Ephx1* and *Ephx2* in visceral adipocytes on day 7 after DSS induction (*n* = 20–25/group).Representative immunoblot of EPHX1 and EPHX2 in gonadal adipose tissues on day 7 after DSS induction. Asterix indicating nonspecific band (*n* = 14–18/group).Schematic overview of cytochrome P450‐EPHX oxylipin pathway.Normalized fold change differences in epoxy fatty acid precursor fatty acids, docosahexaenoic acid (DHA), arachidonic acid (AA), and linoleic acid (LA) in mWAT and gWAT (*n* = 13–14/group).Normalized ratios of epoxy fatty acid to their corresponding diol fatty acid pairs in mWAT and gWAT (*n* = 6–8/group). Data are represented as mean ± s.e.m. (E–H, L) Unpaired Student's *t*‐test. (J, K) Two‐way ANOVA. *P < 0.05, **P < 0.01, ***P < 0.001, ****P < 0.0001. Source data are available online for this figure.

**Figure EV5:**
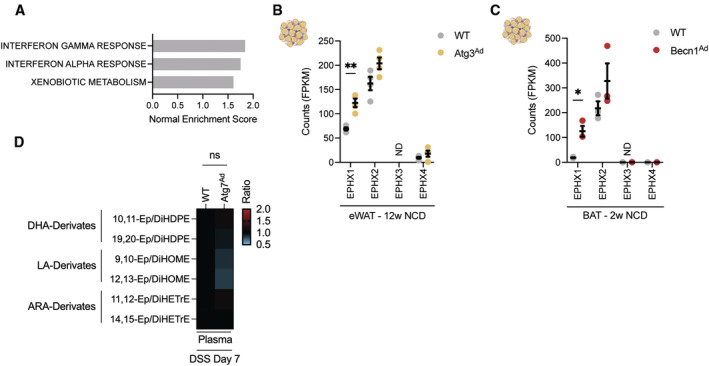
Loss of autophagy‐related genes results in the induction of epoxy hydrolases in adipocytes GSEA enrichment analysis between *Atg7*‐deficient and *Atg7*‐sufficient adipocytes during DSS treatment.Fragments per kilobase of exon per million mapped fragments (FPKM) counts from bulk RNAseq dataset of Cai *et al* (2018) (*n* = 4/group)Fragments per kilobase of exon per million mapped fragments (FPKM) counts from bulk RNAseq dataset of Son *et al* (2020) (*n* = 3/group).Normalized ratios of epoxy fatty acid precursors to their corresponding diol fatty acids pairs in plasma (*n* = 8/group).Data are represented as mean ± s.e.m. (B, C) Unpaired Student's *t*‐test. (D) Two‐way ANOVA. *P < 0.05, **P < 0.01. Source data are available online for this figure.

To get a better understanding of pathways that may be affected by the loss of autophagy in adipocytes, we further analyzed our transcriptomic data by splitting the dataset based on their condition and genotype. Visceral adipocytes from *Atg7* ^*Ad*^ mice had a strong reduction in *Atg7* levels and an increase in estrogen receptor 1 (*Esr1*) expression (Fig 5A and B). The latter was verified to be caused by the overexpression of the Cre‐ERT2 construct which mapped to mouse *Esr1*. Across both treatment groups, we found a total of 32 genes being differentially regulated between WT and *Atg7* ^*Ad*^ visceral adipocytes. Six genes were differentially expressed under both water and DSS treatment conditions (Fig 5C). Using ranked gene set enrichment analysis (GSEA) (Subramanian *et al*, 2005), we found that the xenobiotic pathway was significantly enriched in *Atg7* ^*Ad*^ adipocytes upon DSS‐induced colitis (Figs 5D and EV5A). Enzymes which are known for their role in xenobiotic metabolism, such as the large family of cytochrome P450 monooxygenases and epoxide hydrolases (EPHX), metabolize and detoxify exogenous substrates and mediate the production of oxylipins from endogenous polyunsaturated fatty acids. The expression of many of the key genes involved in these processes are regulated by NRF2, a major transcription factor of the xenobiotic and oxidative stress responses. We found that *Ephx1* was consistently upregulated upon *Atg7* loss in adipocytes (Fig 5C). Remarkably, *Ephx1* expression was also increased in datasets obtained from other studies in which autophagy genes such as *Atg3* and *Beclin‐1* were specifically deleted in adipocytes (Fig EV5B and C) (Cai *et al*, 2018; Son *et al*, 2020). Among the genes that were enriched in *Atg7*‐deficient adipocytes were several other NRF2‐target genes (Fig 5E). In agreement with an activation of the NRF2 pathway, NRF2 protein abundance was increased in *Atg7* ^*Ad*^ visceral adipose tissues (Fig 5F). Specificity for NRF2 activation was further confirmed since only NRF2 target gene *Ephx1* was transcriptionally upregulated, whereas *Ephx2* which is not controlled by NRF2 remained transcriptionally unchanged in autophagy‐deficient adipocytes (Fig 5G). However, both EPHX1 and EPHX2 protein expression were increased in *Atg7* ^*Ad*^ adipose tissues (Fig 5H) suggesting that EPHX2 may be affected by autophagy deletion on a post‐transcriptional level.

EPHX1, together with EPHX2, are central for the enzymatic conversion of cytochrome P450‐derived oxylipins such as epoxy fatty acids (EpFA) to dihydroxy/diol fatty acids (DiolFA). EpFA have strong anti‐inflammatory, analgesic, and hypotensive activity, while DiolFA are less biologically active and are associated with more pro‐inflammatory properties (Fig 5I) (McReynolds *et al*, 2020). In the mesenteric and gonadal adipose tissues, the abundance of nonesterified linoleic acid was reduced (Fig 5J), possibly reflecting their reduced lipolytic capacity. Since we found predominantly changes in EPHX enzyme expression, we tested whether this would shift the balance of oxylipins in the tissue and, possibly, plasma. Indeed, loss of adipocyte autophagy reduced the EpFA:DiolFA ratio, indicating a lower availability of anti‐inflammatory EpFAs in both mesentery and gonadal adipose tissues during DSS colitis (Fig 5K). This was consistently observed for all analyzed DHA‐derived EpFAs which are important substrates for EPHX1 (Fig 5K) (Edin *et al*, 2018). Strikingly, these effects appear to be locally restricted to the adipose tissues since no changes in oxylipin levels were observed in the plasma (Fig EV5D). In summary, these data suggest that loss of adipocyte autophagy activates NRF2 and increased the expression of EPHX enzymes promoting a local imbalance of EpFA:DiolFA. We hypothesize that this imbalance, in turn, might alter the local inflammatory response in the adipose tissue to intestinal inflammation.

### Loss of adipocyte autophagy reduces IL‐10 secretion from adipose tissues and systemic IL‐10 levels upon DSS‐induced colitis

**Figure 6:**
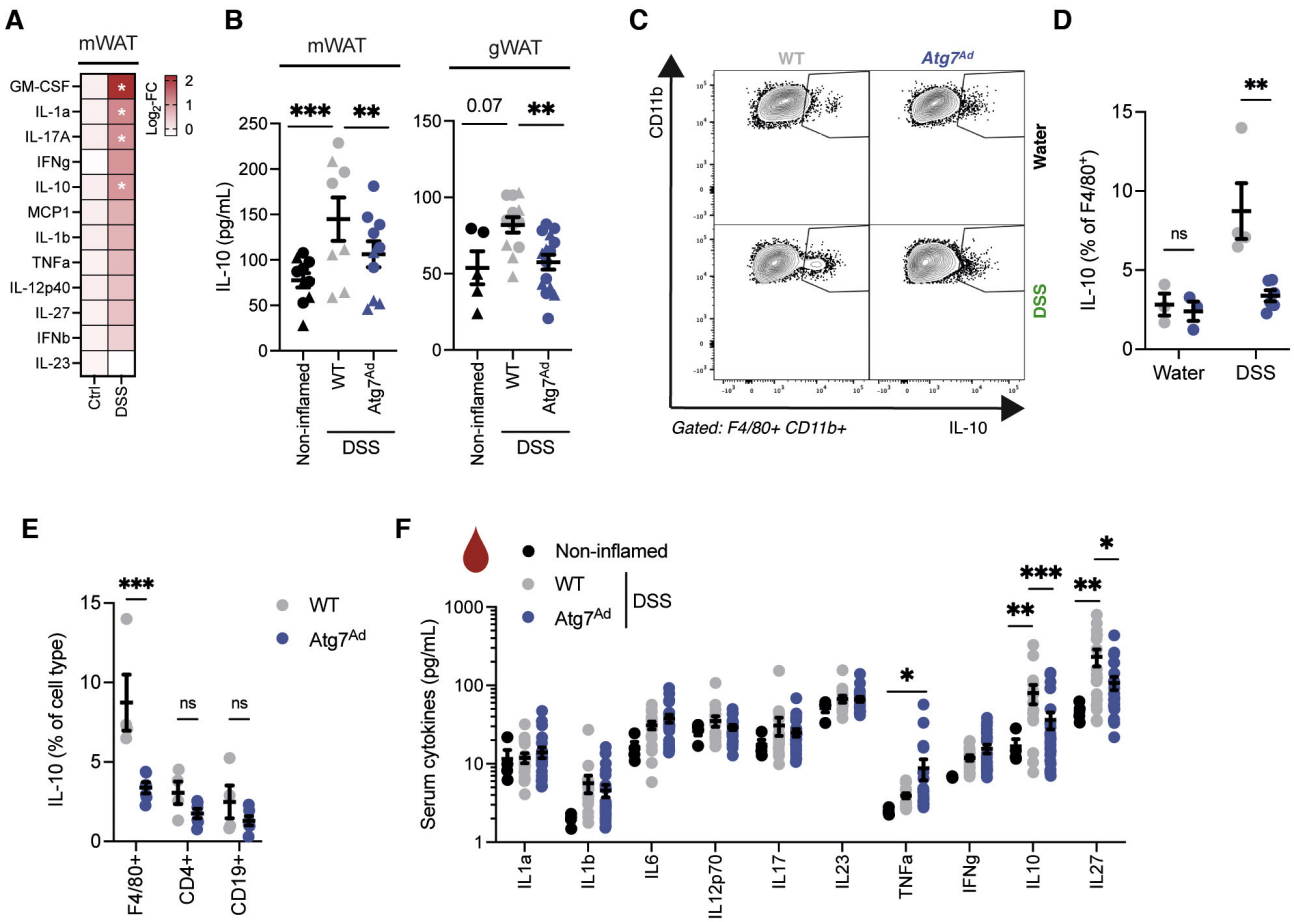
Reduced adipose tissue‐derived IL‐10 secretion and IL‐10 serum levels in adipocyte autophagy‐deficient mice during DSS‐induced colitis Colitis was induced in mice for 7 days and mesenteric adipose tissue explants were cultured with FBS. Cytokine secretion into the supernatant was measured after 24 h of culture (*n* = 4–12/group).Colitis was induced in mice for 7 days and adipose tissues were extracted and cultured for 6 h in serum‐starved medium. Secretion of IL‐10 and from mesenteric (left panel) and gonadal adipose tissues (right panel) was measured by ELISA. Shapes identify individual experiments (*n* = 5–15/group).Representative FACS plots of CD11b^+^ F4/80^+^ ATMs in visceral adipose tissue from WT and *Atg7* ^*Ad*^ mice upon DSS‐induced colitis on day 7.Quantification of IL‐10‐producing ATMs in visceral adipose tissue from WT and *Atg7* ^*Ad*^ upon DSS‐induced colitis on day 7 (*n* = 3–6/group).Frequencies of IL‐10‐producing immune cells in visceral adipose tissues from WT and *Atg7* ^*Ad*^ upon DSS‐induced colitis by flow cytometry (*n* = 4–6/group).Serum cytokines upon DSS‐induced colitis on day 7 postinduction (*n* = 17–23/group). Data are represented as mean ± s.e.m. (A) Multiple *t*‐test. (D–F) Two‐way ANOVA. (B) Two‐way ANOVA with regression for experiment. *P < 0.05, **P < 0.01, ***P < 0.001. Source data are available online for this figure.

Since the changes in EpFA:DiolFA appeared to be locally restricted, we next wanted to identify which soluble factors may impact on gut inflammation. Evidence suggests that stimulation of macrophages with EpFA promotes the production of IL‐10, while abundance of DiolFA can quench IL‐10 production (McDougle *et al*, 2017; Levan *et al*, 2019). Thus, we next tested whether cytokine production from the adipose tissue may contribute to systemic inflammation during DSS‐induced colitis and assessed the secreted cytokine profile from mesenteric adipose tissues. We found that the mesenteric adipose tissue increases the secretion of several cytokines including the anti‐inflammatory cytokine IL‐10 in response to DSS (Fig 6A). Next, we tested whether IL‐10 secretion from the mesenteric and gonadal adipose tissue was affected by adipocyte autophagy loss. Remarkably, disruption of adipocyte autophagy abolished DSS‐induced IL‐10 secretion from both mesenteric and gonadal adipose tissues (Fig 6B). We found that, upon DSS‐induced colitis, CD11b^+^ F4/80^+^ adipose tissue macrophages (ATMs) are one of the major cell populations producing IL‐10 in visceral adipose tissues (Fig 6C and D), whereas IL‐10 production in T and B cells were unaltered (Fig 6E). In adipose tissues, ATM frequencies were increased by DSS (Appendix Fig S1A), which resulted in an increased presence of crown‐like structures, however, this was comparable between wild‐type and *Atg7* ^*Ad*^ mice. EpFA can alter macrophage polarization and increase tissue‐resident macrophage marker expression such as CD206 (Lopez‐Vicario *et al*, 2015). In line with reduced EpFA levels in the adipose tissue, *Atg7* ^*Ad*^ ATMs had a slightly reduced CD206 expression (Appendix Fig S1B) but remained the predominant type of macrophage in the tissue. In addition, expression of CD36, a lipid scavenging receptor which is commonly found on M2‐type macrophages and induced on ATMs during lipolysis, was increased on the surface of ATMs in wild‐type mice during DSS colitis. However, CD36 expression was not increased in *Atg7* ^*Ad*^ mice (Appendix Fig S1C), indicating a distinct adaptation to different lipid availability in the adipose tissue.

Due to the important role of IL‐10 in immune tolerance, we hypothesized that the reduction of IL‐10 secretion from adipose tissues may translate into a systemic reduction of circulating IL‐10 levels. Indeed, we found that while circulating IL‐10 levels were significantly upregulated in DSS‐treated wild‐type mice compared to noninflamed mice, IL‐10 levels were diminished in *Atg7* ^*Ad*^ mice (Fig 6F). Taken together, these data suggest that adipose tissues from adipocyte autophagy‐deficient mice have an impaired production and secretion of anti‐inflammatory IL‐10 in DSS‐induced colitis compared to wild‐type mice.

### Cytochrome P450‐EPHX pathway regulates IL‐10 secretion from autophagy‐deficient adipocytes during intestinal inflammation

**Figure 7:**
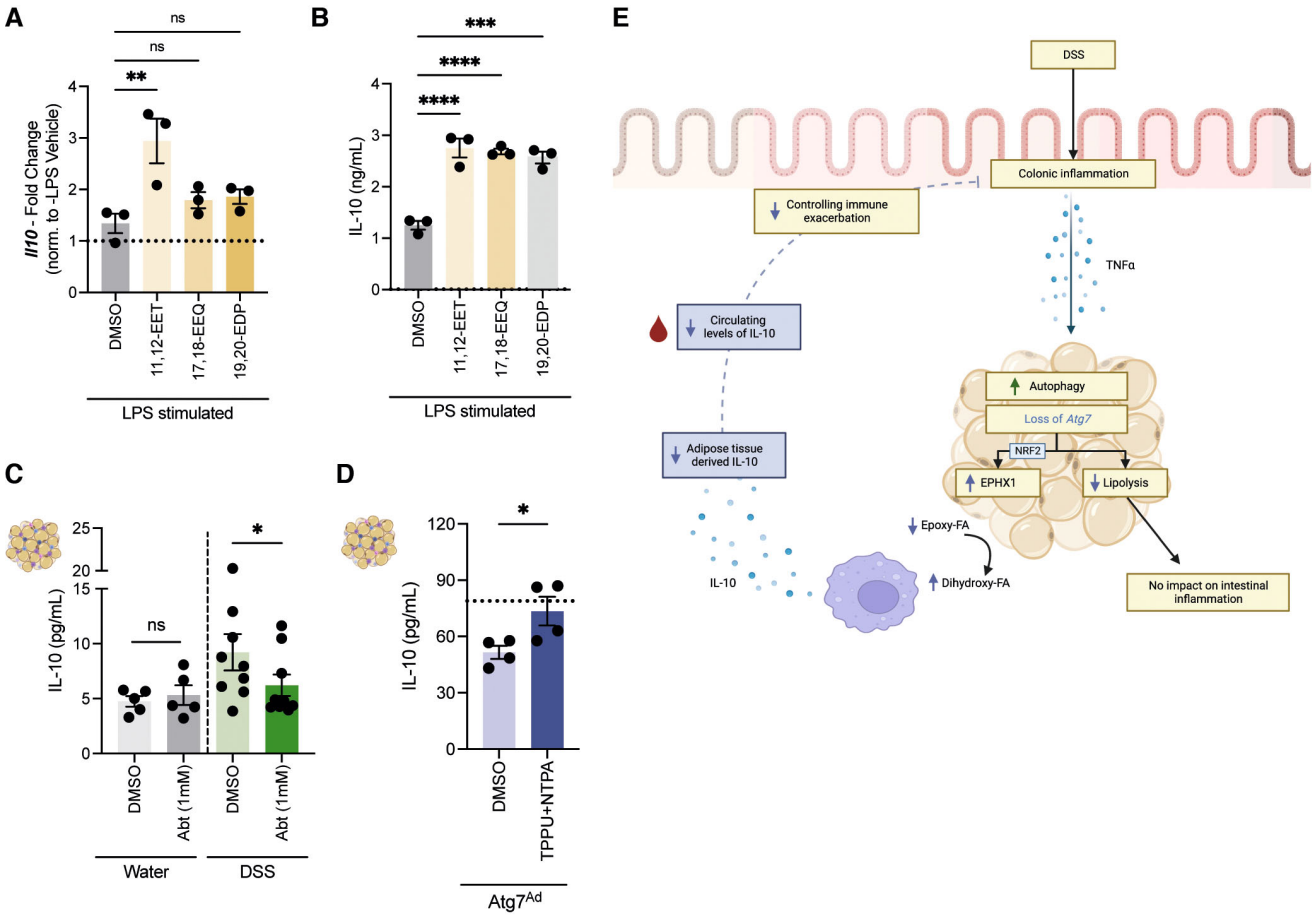
Cytochrome P450‐EPHX pathway regulates IL‐10 secretion from autophagy‐deficient adipose tissues upon DSS‐induced intestinal inflammation Quantification of *Il10* transcript levels in RAW264.7 upon stimulation to epoxy fatty acids (*n* = 3/group).Quantification of IL‐10 protein levels in the supernatant of RAW264.7 upon stimulation to epoxy fatty acids (*n* = 3/group).Quantification of IL‐10 protein levels in the supernatant of *ex vivo* cultured adipose tissues from water‐ or DSS‐treated wild‐type mice in the absence or presence of the cytochrome P450 inhibitor 1‐ABT (*n* = 5–9/group).Quantification of IL‐10 protein levels in the supernatant of *ex vivo* cultured adipose tissues from DSS‐induced *Atg7* ^*Ad*^ mice in the absence or presence of the EPHX1 inhibitor NTPA and EPHX2 inhibitor TPPU (*n* = 4/group). Dotted line represents IL‐10 secretion from adipose tissues of DSS‐induced wild‐type mice.Graphical summary of the anti‐inflammatory fat‐gut crosstalk during intestinal inflammation. Designed using BioRender. Data are represented as mean ± s.e.m. (A, B) One‐Way ANOVA. (C, D) Paired Student's *t*‐test. *P < 0.05, **P < 0.01, ***P < 0.001, ****P < 0.0001. Source data are available online for this figure.

To establish a more mechanistic link between the increased function of the cytochrome P450‐EPHX pathway and IL‐10 in adipose tissues, we first determined whether EpFA supplementation improves IL‐10 production from macrophages *in vitro*. Pretreatment of RAW264.7 macrophages with different EpFA increased *Il10* transcript levels upon LPS stimulation (Fig 7A), which was further confirmed on protein level in the supernatant (Fig 7B). Cytochrome P450 enzymes are key for the production of EpFA. Inhibition of cytochrome P450 resulted in a marked reduction of IL‐10 secretion from DSS‐induced wild‐type adipose tissues (Fig 7C), suggesting that cytochrome P450 is crucial for adipose tissue‐derived IL‐10 during intestinal inflammation. Lastly, blockade of EPHX1 and EPHX2 in *Atg7* ^*Ad*^ adipose tissue explants rescued IL‐10 production (Fig 7D), establishing that EPHX enzyme activity can control IL‐10 in autophagy‐deficient adipose tissues. Collectively, these data indicate that EpFA and enzymes controlling their production and degradation can alter adipose tissue IL‐10 levels during intestinal inflammation and this is dependent on autophagy (Fig 7E).

## Discussion

Immune cells reside within distinct tissue environments, however, the impact of local metabolic cues on inflammatory processes remains incompletely understood (Richter *et al*, 2018). Our results indicate that autophagy in mature adipocytes contributes to the balance of intratissual oxylipin levels. Furthermore, we demonstrate that adipocytes autophagy is part of the anti‐inflammatory immune response to intestinal inflammation by promoting the release of IL‐10 from adipose tissues. Autophagy‐dependent secretion from adipose tissues contributes to systemic IL‐10 levels, and limits inflammation at a distant tissue site, the colon. Therefore, our study provides novel insights into a cross‐tissue anti‐inflammatory mechanism, enabling the development of therapeutic approaches to target this crosstalk.

While polymorphisms in autophagy genes are well established as genetic risk factors for IBD, little is known about autophagy's role in adipocytes in this disease. Our study highlights that autophagic flux is increased in visceral adipose tissues of wild‐type mice upon DSS‐induced colitis and in the creeping fat tissue of CD patients. Since tamoxifen itself induces autophagy, it is possible that tamoxifen may potentiate some of the effects observed in the DSS‐treated wild‐type and *Atg7* ^*Ad*^ mouse model. However, the induction of autophagy was also found in wild‐type mice, which have not received tamoxifen, alongside a transcriptional increase of *Atg8* homologs. This increase parallel findings during muscle atrophy, where the expression of *Map1lc3b*, *Gabarapl1*, *Bnip3*, *Bnip3l*, and *Vps34* is regulated via FOXO3 activation, which subsequently controls autophagy levels (Mammucari *et al*, 2007). It appears plausible that a similar FOXO3‐dependent mechanism occurs in adipocytes, especially since visceral adipocytes showed several transcriptomic and macroscopic changes reminiscent of a “cachexia‐like” phenotype. In line with this, we found that inflammatory cues such as TNFα can promote systemic inflammation which is required for the induction of adipocyte autophagy and cachexia‐like phenotype (Rivera *et al*, 2019).

Early studies found that autophagy is crucial for the normal differentiation of adipose tissues *in vivo* (Singh *et al*, 2009; Zhang *et al*, 2009). However, the significance of autophagy in mature adipocytes remained unexplored until recently. Postdevelopmental ablation of autophagy in mature adipocytes decreased β‐adrenergic receptor‐induced lipolysis (Cai *et al*, 2018; Son *et al*, 2020). Conversely, disruption of mTOR by genetic deletion of *Raptor* increases lipolytic output via autophagy (Zhang *et al*, 2020). It is likely that adipocyte autophagy controls lipolytic output via the degradation of key proteins involved in the lipolytic machinery such as described for perilipins in fibroblasts and adipocytes (Kaushik & Cuervo, 2016; Ju *et al*, 2019). In addition, we found that phosphorylation of HSL was reduced in autophagy‐deficient adipocytes, which could indicate a role of autophagy in regulating upstream kinase activity, such as PKA, during intestinal inflammation. Moreover, we discovered that adipocyte autophagy can indeed regulate TNFα‐induced lipolysis and by this may fine‐tune lipolytic output of adipocytes upon inflammatory stress conditions.

Adipocyte lipolysis during DSS‐induced colitis is driven predominantly through ATGL. Somewhat surprisingly, loss of adipocyte lipolysis had no impact on body weight loss or colonic inflammation, thus raising the question whether adipocyte lipolysis is beneficial or maladaptive in the context of this disease. These observations are reminiscent of findings during infection‐associated cachexia, where deletion of the cytosolic lipases *Atgl* and *Hsl* had no impact on body weight loss (Baazim *et al*, 2019). In contrast, during cancer‐associated cachexia, loss of these lipases prevents body weight loss suggesting that infection and inflammation models of cachexia act through distinct and yet to be identified biological pathways (Das *et al*, 2011).

Lipid uptake occurs across the intestine with highest levels of lipid absorption in the proximal small intestine. DSS leads to a disruption of the epithelial barrier towards the distal colon. However, some studies report that DSS can alter the morphology of the small intestine such as the jejunum, affecting its function and possibly lower dietary lipid absorption (Yazbeck *et al*, 2011). This could, therefore, alter uptake of dietary FFA, although FFA absorption in the context of DSS‐induced colitis has not been conclusively determined. The observed decline in serum FFA levels may be connected to a reduced food intake during DSS‐induced colitis (Vidal‐Lletjos *et al*, 2019), their possible reduced absorption, and the depletion of lipid stores, such as gonadal adipose tissues. The induction of autophagy in the adipose tissue may help to maintain circulating FFA levels in addition to curb inflammation through signaling lipids.

Loss of adipocyte autophagy increased NRF2 stability, likely through the sequestration of its regulator KEAP1 (Cai *et al*, 2018). Here, we demonstrate for the first time that this antioxidant/xenobiotic pathway exacerbates an inflammatory disease. Increased expression of EPHX1 was paralleled by an imbalance in oxylipins leading to decreased levels of EpFA and increased DiolFA. Similar to our findings, EPHX1 was recently found to convert in particularly omega‐3 DHA substrates in adipocytes and liver (Edin *et al*, 2018; Gautheron *et al*, 2021). Since our data suggest a broader dysregulation of EpFA:DiolFA, it is likely that EPHX2, which was accumulated on protein level in *Atg7* ^*Ad*^ adipose tissues, may also contribute to the conversion of oxylipin substrates. Increasing evidence suggests that macrophages are regulated by oxylipins in their environment. Indeed, increased presence of omega‐3‐derived EpFA achieved either through inhibition of EPHX2 or through supplementation has been shown to promote CD206 expression and IL‐10 secretion (Lopez‐Vicario *et al*, 2015; McDougle *et al*, 2017). In line with the reduced presence of EpFA in *Atg7*‐deficient adipose tissue, we found these two hallmarks of anti‐inflammatory macrophages were equally decreased. Other oxylipin species, produced via the LOX and COX pathway, can also modulate DSS‐induced colitis (Willenberg *et al*, 2015; Crittenden *et al*, 2021). However, most genes involved in these pathways were reduced in visceral adipocytes during DSS‐induced colitis. It is plausible, however, that other oxylipins may contribute to the observed phenotype in the *Atg7* ^*Ad*^ mouse model.

Importantly, this study underscores the importance of adipose tissue‐derived IL‐10 in controlling disease severity. Our findings of increased IL‐10 secretion in visceral adipose tissues upon intestinal inflammation, confirmed findings from the Siegmund laboratory that mesenteric ATMs upregulate expression of IL‐10 during intestinal inflammation in both human and mouse (Batra *et al*, 2012; Kredel *et al*, 2013). In line with this, global loss of IL‐10 leads to exacerbation of intestinal inflammation (Li *et al*, 2014). The disruption in systemic IL‐10 levels may also explain the reduced colonic expansion of FOXP3^+^ Tregs at resolution, since adequate IL‐10 signaling is required for the expression of FOXP3 in intestinal Tregs (Murai *et al*, 2009). Recent single‐cell transcriptomic analysis of immune cells resident in human creeping fat tissues revealed an anti‐inflammatory and prorepair role of ATMs, further supporting their beneficial role during intestinal inflammation (Ha *et al*, 2020). Our data highlight how adipocyte dysfunction can impair this adipocyte‐immune cell crosstalk suggesting that this communication may also exist in human pathology.

While ATMs accumulate in in creeping fat tissues of CD patients and in the mesentery of mice upon DSS‐induced colitis (Batra *et al*, 2012), it remains unclear how these macrophages are regulated during intestinal inflammation. We propose that oxylipins can shift macrophage polarization, in part, through their action as PPAR ligands (Overby *et al*, 2020), which are important regulators of M2‐type polarization and function (Odegaard *et al*, 2007, 2008). We found that the PPARγ‐target gene CD36 is upregulated during DSS‐induced colitis on ATMs in wild‐type mice and but not in ATMs from *Atg7* ^*Ad*^ mice. Similarly, the expression of CD36 on adipocytes can be controlled by oxylipin levels (Lynes *et al*, 2017). In addition, it is possible that oxylipin imbalance may also affect other IL‐10‐producing cell types in the adipose tissues such as Tregs which also rely on PPARγ for their accumulation and function (Cipolletta *et al*, 2012). The resulting reduction in systemic IL‐10 levels prolongs pro‐inflammatory programs at the distal inflammation site. As such, IL‐10 signaling is required for intestinal macrophages to prevent pro‐inflammatory exacerbation during DSS‐induced colitis through inhibition of mTOR signaling which controls macrophage pro‐inflammatory activity (Li *et al*, 2014; Ip *et al*, 2017).

Overall, this study reveals that metabolically healthy adipose tissues are important regulators to prevent excessive inflammation during colitis. However, the function of adipose tissues in IBD may depend on the overall metabolic and disease state. The expansion of the mesentery during CD may initially be beneficial through prevention of bacterial translocation and signaling pathways poised to promote anti‐inflammatory pathways, as shown here (Batra *et al*, 2012; Ha *et al*, 2020). However, sustained inflammation may ultimately subvert the function of the mesentery and lead to adipose tissue fibrosis and intestinal strictures (Mao *et al*, 2019). Sustained tissue fibrosis results in tissue hypoxia (Zuo *et al*, 2016) and may impact on tissue oxylipin levels in creeping fat of CD patients.

Here, we demonstrate for the first time that adipocyte autophagy contributes to the intratissual balance of oxylipin levels and thus controls the anti‐inflammatory immune response to intestinal tissue injury through regulation of adipose tissue‐derived IL‐10 (as summarized in Fig 7E). It underlines the importance of local adipocyte‐immune cell crosstalk through regulation of lipid mediators. This may present a broader local metabolic regulatory pathway to control immune responses to inflammation and infection.

## Materials and Methods

### Mice

*Adipoq‐Cre* ^*ERT2*^ mice (Sassmann *et al*, 2010) were purchased from Charles River, UK (JAX stock number: 025124) and were crossed to *Atg7* floxed mice (Komatsu *et al*, 2005). Experimental cages were sex‐ and age‐matched and balanced for genotypes. Genetic recombination was induced at 8–10 weeks of age by oral gavage of 4 mg tamoxifen per mouse for 5 consecutive days. All experimental procedures were conducted 2 weeks after last tamoxifen administration. DSS‐induced colitis was induced by 1.5–2% (w/v) DSS (MP Biomedicals, 160110) in drinking water. Mice treated with neutralizing antibodies received 0.5 mg/mouse of either anti‐mouse TNFα (Bio X Cell, BE0058) or IgG1 isotype control (Bio X Cell, BE0088) on day 0 by intraperitoneal injection. Mice were treated with DSS for 5 days and assessed on day 7, a peak inflammation time, or on day 14, a resolution time point. Constitutive *Adipoq‐Cre* × *Pnpla2* floxed mice (Sitnick *et al*, 2013; Schoiswohl *et al*, 2015) (JAX stock number: 024278) were kindly provided by Prof. Rudolph Zechner. Wild‐type C57BL/6J mice were purchased from Charles River, UK (JAX stock number: 0000664) or bred in‐house. Mice were housed on a 12 h dark/light cycle and fed *ad libitum*, under specific pathogen‐free conditions. All animal experimentation was performed in accordance to approved procedures by the Local Review Committee and the Home Office under the project license (PPL30/3388 and P01275425).

### Human samples

Three individuals with Crohn's disease were recruited for this study. Patients were 30, 31, and 52 years old (all women) and were diagnosed with ileal stenosis at the time of ileocecal resection. At the time of surgery, patients were treated with Prednisolon and/or Azathiporin. Creeping fat was harvested from the ileum, while adjacent noninflamed mesenteric adipose tissues were collected from the caecum. As a further control, mesenteric adipose tissue from the caecum was collected from one colorectal cancer patient (female, 56 years old) undergoing right hemicolectomy. All patients gave informed consent in the framework of the IBDome (SFB‐TRR 241 B01). Tissue collection and ethics were approved by the institutional review board of the Charité‐Universitätsmedizin Berlin (Ethics Approval: EA1/200/17) and in line with the principles set out in the WMA Declaration of Helsinki and the Department of Health and Human Services Belmont Report. Adipose tissue explants (~50–100 mg) were cultured for DMEM supplemented with 10% FBS (Sigma, F9665) and 100 U/ml Pen‐Strep for 4 h in the absence or presence of lysosomal inhibitors 100 nM Bafilomycin A1 and 20 mM ammonium chloride, then snap frozen until extraction for immunoblotting.

### Histopathology assessment

Distal, mid, and proximal colon pieces were fixed in 10% neutral buffered formalin for 24 h before washed and transferred into 70% ethanol. Tissue pieces from each sample were embedded in the same paraffin block and 5 μm sections were subsequently stained with hematoxylin and eosin (H&E). Scoring of histology sections was executed in a blinded fashion according to a previously reported scoring system (Dieleman *et al*, 1998). In brief, each section was assessed for the degree inflammation, the depth of tissue damage, possible crypt damages, with high scores signifying increased tissue damage. In addition, signs of regeneration (epithelial closure, crypt regeneration) were assessed, with high scores indicating delayed regeneration. Changes were multiplied with a factor classifying the involvement tissue area. Total score was calculated and presented.

### Adipose tissue and colon digestion

We collected mesenteric adipose tissue separate from a collective set of visceral adipose tissue depots (including omental, gonadal, and retroperitoneal adipose tissue) to distinguish proximal versus distal effects of intestinal inflammation on adipose tissues. Adipose tissues were collected and digested in DMEM containing 1% fatty acid‐free BSA (Sigma, 126609), 5% HEPES (Gibco, 15630‐056), 0.2 mg/ml Liberase TL (Roche, 5401020001), and 20 μg/ml DNaseI (Roche, 11284932001). Tissues were minced in digestion medium and incubated for 25–30 min at 37°C at 180 rpm. Tissues were further broken down by pipetting using wide bore tips and filtered through a 70 μm mesh. Digestion was quenched by adding medium containing 2 mM EDTA. Adipocyte and stromal vascular fraction were separated by centrifugation (700 *g*, 10 min) and collected for further downstream analysis.

Colon digestions were performed as previously described (Danne *et al*, 2017). Colons were opened longitudinally and fecal content was removed by washing with PBS. Then colons were washed twice in RPMI containing 5% FBS and 5 mM EDTA at 37°C under agitation. Tissues were minced and digested in RPMI supplemented with 5% FBS, 1 mg/ml collagenase type VIII (Sigma), and 40 μg/ml DNaseI (Roche). Cell suspension was strained through 40 μm mesh and cells were subjected to downstream analysis.

### Flow cytometry

Flow cytometry staining was performed as previously described (Riffelmacher *et al*, 2017). Surface staining was performed by incubating cells with fluorochrome‐conjugated antibodies (Biolegend, BD Bioscience, eBioscience) and LIVE/DEAD Fixable Stains (ThermoFischer) and Fc receptor blockade (ThermoFisher, 14‐0161‐85) for 20 min at 4°C. Cells were fixed with 4% PFA for 10 min at room temperature. For intracellular staining of transcription factors, cells were fixed/permeabilized using the eBioscience™ Foxp3/ Transcription Factor Staining Set (00‐5523‐00, Invitrogen). For cytokine staining, cells were stimulated using Cell Activation cocktail (Biolegend) for 4 h at 37°C in RPMI containing 10% FBS. After surface staining, cells were fixed and stained in Cytofix/CytoPerm (BD Bioscience) following manufacturer protocol. Samples were acquired on LSRII or Fortessa X‐20 flow cytometers (BD Biosciences). Flow cytometry antibodies were purchased from Biolegend or eBioscience or BD Biosciences. The following clones were used for immune phenotyping: CD45 (30‐F11), CD11b (M1/70), CD11c (N418), Siglec‐F (S17007L), IA/IE (M5/114.14.2), Ly6C (HK1.4), Ly6G (1A8), F4/80 (BM8), CD19 (6D5), CD4 (GK1.5), CD8 (53‐6.7), Foxp3 (FJK‐16s), Gata3 (TWAJ), RORgt (Q31‐378), TCRb (H57‐597), IL‐10 (JES5‐16E3), CD206 (C068C2), CD36 (HM36).

### Quantitative PCR

Adipocytes and adipose tissue RNA were extracted using TRI reagent (T9424, Sigma). Colon tissue RNA were extracted in RLT buffer containing 1,4‐Dithiothreitol. Tissues were homogenized by lysis in 2 ml tubes containing ceramic beads (KT03961‐1‐003.2, Bertin Instruments) using a Precellys 24 homogenizer (Bertin Instruments). RNA was purified following RNeasy Mini Kit (74104, Qiagen) manufacturer instructions. cDNA was synthesized following the High‐Capacity RNA‐to‐cDNA™ kit protocol (4388950, ThermoFischer). Gene expression was assessed using validated TaqMan probes and run on a ViiA7 real‐time PCR system. All data were collected by comparative Ct method either represented as relative expression (2^−ΔCt^) or fold change (2^−ΔΔCt^). Data were normalized to the two most stable housekeeping genes; for adipose tissues *Tbp* and *Rn18s* and for colon *Actb* and *Hprt*. The following TaqMan probes were used for the quantification: Atg7 (Mm00512209_m1), Map1lc3b (Mm00782868_m1), Gabarap (Mm00490680_m1), Gabarapl1 (Mm00457880_m1), Gabarapl2 (Mm01243684_gH), Il33 (Mm00505403_m1), Tnfa (Mm00443258_m1), Ifng (mm01168134_m1), Cxcl9 (Mm00434946_m1), Il1a (Mm00439620_m1), Ptx3 (Mm00477268_m1), Il10 (Mm00439614_m1), Pnpla2 (Mm00503040_m1), Ephx1 (Mm00468752_m1), Ephx 2 (Mm01313813_m1), Actin (Mm02619580_g1), Hprt (Mm01545399_m1), Rn18s (Mm04277571_s1), Tbp (Mm01277042_m1).

### Bulk RNA sequencing

Visceral adipocytes were isolated as floating fraction upon digestion. RNA was extracted and converted to cDNA as described above. PolyA libraries were prepared through end reparation, A‐tailing and adapter ligation. Samples were then size‐selected, multiplexed, and sequenced using a NovaSeq6000. Raw read quality control was performed using pipeline readqc.py (https://github.com/cgat‐developers/cgat‐flow). Resulting reads were aligned to GRCm38/Mm10 reference genome using the pseudoalignment method kallisto (Bray *et al*, 2016). Differential gene expression analysis was performed using DEseq2 v1.30.1 (Love *et al*, 2014). Pathway enrichment analysis was performed on differentially expressed genes for “Biological Pathways” using clusterProfiler (v4.0) R package (Wu *et al*, 2021). DESeq2 median of ratios were used for visualization of expression levels. Heatmaps of selected gene sets were presented as *z*‐scores using R package pheatmap. Gene enrichment analysis was performed using GSEA software using Hallmark gene sets (Subramanian *et al*, 2005). Transcriptomic dataset is available on EBI ArrayExpress (Access Code: E‐MTAB‐12498). R code is available under https://github.com/cleete/IBD‐Adipocyte‐Autophagy.

### Lipolysis assays

Adipose tissues were collected and washed in PBS before subjected to lipolysis assays. For isoproterenol stimulation, adipose tissues were cut into small tissue pieces and incubated in serum‐free DMEM–High Glucose (Sigma, D5796) with 2% fatty acid‐free BSA (Sigma, 126579) in the absence or presence of 10 μM isoproterenol (Sigma, I6504) for the indicated time. TNFα‐induced lipolysis was induced as previously described (Ju *et al*, 2019). In brief, small adipose tissue pieces were cultured in DMEM–High Glucose for 24 h in the absence or presence of 100 ng/ml recombinant TNFα (Peprotech, 315‐01A) and then transferred into serum‐free DMEM containing 2% fatty acid‐free BSA for 3 h. Supernatants were collected and FFA concentration normalized to adipose tissue input.

### Adipose tissue explant cultures

Gonadal or mesenteric adipose tissue explants were collected from mice at indicated time points. For autophagic flux measurements, explants (~50–100 mg) were cultured for DMEM supplemented with 10% FBS (Sigma, F9665) and 100 U/ml Pen‐Strep for 4 h in the absence or presence of lysosomal inhibitors 100 nM Bafilomycin A1 and 20 mM ammonium chloride. Explants were washed in PBS before collection and then frozen at −80°C until proteins were extracted for immunoblotting. For measurement of cytokine secretion, adipose tissue explants were cultured for 6 h in DMEM/High Modified (D6429, Sigma) with 100 U/ml Pen‐Strep in the absence of FBS. For inhibition of cytochrome P450 *ex vivo*, medium was supplemented with 1‐ABT (Sigma, A3940) at a final concentration of 1 mM. For inhibition of EPHX1 and EPHX2, medium was supplemented with NTPA (kindly received from Christophe Morriseau and Bruce D. Hammock) and TPPU (Sigma, SML0750) at a final concentration of 100 and 10 μM, respectively. Supernatant was collected, spun down (400 *g*, 5 min) to remove cell debris and then frozen until further analysis.

### Cell culture

RAW264.7 cells (Sigma, 91062702) were cultured in DMEM supplemented with 2% glutamine, 10% FBS, and 100 U/ml Pen‐Strep. Cells were seeded in 24‐well plates (Sarstedt, 83.3922) at 100,000 cells/well. After 4 days, when cells reached confluence, medium was removed, and fresh medium supplemented with corresponding epoxy fatty acids (11,12‐EET Max Spec (Cayman Chemical, 10007262‐100), 17,18‐EpETE (EEQ) Max Spec (Cayman Chemical, 25367‐100), 19,20‐EDP Max‐Spec (Cayman Chemical, 25272‐100)) were added at a final concentration of 1 μM for 3 h, then cells were stimulated with 1 μg/ml LPS (Sigma, L8274) for indicated times. For qPCR measurement, cells were cultured for an additional 3 h after LPS stimulation and 12 h for subsequent ELISA readout.

### Free fatty acid analysis

Total supernatant and serum FFA levels were measured using Free Fatty Acid Assay Quantification Kit (ab65341, Abcam). For detailed analysis of FFA species, lipids were extracted by Folch's method (Folch *et al*, 1957) and subsequently run on a one‐dimensional thin layer chromatography (TLC) using a 10 × 10 cm silica gel G plate in a hexane/diethyl ether/acetic acid (80:20:1, by vol.) solvent system. Separated FFA were used for fatty acid methyl esters (FAMEs) preparation through addition of 2.5% H_2_SO_4_ solution in dry methanol/toluene (2:1 (v/v)) at 70°C for 2 h. A known amount of C17:0 was added as an internal standard for quantification. FAMEs were extracted with HPLC grade hexane. A Clarus 500 gas chromatograph with a flame ionizing detector (FID) (Perkin‐Elmer) and fitted with a 30 m × 0.25 mm i.d. capillary column (Elite 225, Perkin Elmer) was used for separation and analysis of FAs. The oven temperature was programmed as follows: 170°C for 3 min, increased to 220°C at 4°C/min, and then held at 220°C for 15 min. FAMEs were identified routinely by comparing retention times of peaks with those of G411 FA standards (Nu‐Chek Prep Inc). TotalChrom software (Perkin‐Elmer) was used for data acquisition and quantification.

### Oxylipin analysis

Oxylipins were analyzed by means of liquid chromatography mass spectrometry (Rund *et al*, 2018; Kutzner *et al*, 2019). The plasma samples were analyzed following protein precipitation and solid‐phase extraction on reversed phase/anion exchange cartridges (Rund *et al*, 2018; Kutzner *et al*, 2019). The adipose tissue was homogenized in a ball mill and oxylipins and nonesterified fatty acids were extracted with a mixture of chloroform and *iso*‐propanol following solid‐phase extraction on an amino propyl SPE cartridge (Koch *et al*, 2021, 2022). Oxylipin concentrations in adipose tissue and plasma as well as LA, DHA, and ARA in the tissue were determined by external calibration with internal standards (Rund *et al*, 2018; Kutzner *et al*, 2019).

### Immunoblotting

Autophagic flux in adipose tissues was measured by incubating adipose tissue explants from experimental animals in RPMI in the absence or presence of lysosomal inhibitors 100 nM Bafilomycin A1 and 20 mM NH_4_Cl for 4 h. DMSO was used as ‘vehicle’ control. Adipose tissues were collected and snap frozen. Protein extraction was performed as previously described (An & Scherer, 2020). In brief, 500 μl of lysis buffer containing protease inhibitors (04693159001, Roche) and phosphoStop (04906837001, Roche) were added per 100 mg of tissue. Cells were lysed using Qiagen TissueLyser II. Tissues were incubated on ice for 1 h and lipid contamination was removed via serial centrifugation and transfer of internatant into fresh tubes. Protein concentration was determined by BCA Protein Assay Kit (23227, Thermo Scientific). A total of 15–30 μg protein was separated on a 4–12% Bis‐Tris SDS–PAGE and transferred using BioRad Turbo Blot (BioRad, 1704156) or wet transfer onto PVDF membrane. Human tissues were separated on a 4–20% Tris–Glycine gel (BioRad, 4561093DC) and transferred onto PVDF membranes. Membranes were blocked in TBST containing 5% BSA. Primary antibodies were used at indicated concentration overnight: LC3 (L8918, Sigma) (1:1,500); β‐ACTIN (8H10D10, Cell Signaling) (1:5,000); EPHX1 (Santa Cruz, sc‐135984) (1:500); EPHX2 (Santa Cruz, sc‐166961) (1:500); NRF2 (GeneTex, GTX103322) (1:1,000); pHSL‐Ser600 (Cell Signaling, 45804) (1:2,000); HSL (Cell Signaling, 18381) (1:2,000); ATGL (Cell Signaling, 2439) (1:1,500). Membranes were visualized using IRDye secondary antibodies (1:10,000) (LICOR). Band quantification of Western blots was performed on ImageStudio software (LICOR). Autophagic flux was calculated using LC3‐II normalized values to β‐ACTIN loading control: (LC3‐II (Inh) – LC3‐II (Veh))/(LC3‐II (Veh)), as previously described (Zhang *et al*, 2019).

### Transmission electron microscopy

Mice were sacrificed by increasing concentrations of CO_2_. Adipose tissues were excised, cut into small 1–2 mm pieces and immediately fixed in prewarmed (37°C) primary fixative containing 2.5% glutaraldehyde and 4% formaldehyde in 0.1 M sodium cacodylate buffer, pH 7.2 for 2 h at room temperature and then stored in the fixative at 4°C until further processing. Samples were then washed for 2× 45 min in 0.1 M sodium cacodylate buffer (pH 7.2) at room temperature with rotation, transferred to carrier baskets and processed for EM using a Leica AMW automated microwave processing unit. Briefly, this included three washes with 0.1 M sodium cacodylate buffer, pH 7.2, one wash with 50 mM glycine in 0.1 M sodium cacodylate buffer to quench free aldehydes, secondary fixation with 1% osmium tetroxide +1.5% potassium ferricyanide in 0.1 M sodium cacodylate buffer, six water washes, tertiary fixation with 2% uranyl acetate, two water washes, then dehydration with ethanol from 30, 50, 70, 90, 95 to 100% (repeated twice). All of these steps were performed at 37°C and 15–20 W for 1–2 min each, with the exception of the osmium and uranyl acetate steps, which were for 12 and 9 min, respectively. Samples were infiltrated with TAAB Hard Plus epoxy resin to 100% resin in the AMW and then processed manually at room temperature for the remaining steps. Samples were transferred to 2 ml tubes filled with fresh resin, centrifuged for ~2 min at 2,000 *g* (to help improve resin infiltration), then incubated at room temperature overnight with rotation. The following day, the resin was removed and replaced with fresh resin, then the samples were centrifuged as above and incubated at room temperature with rotation for ~3 h. This step was repeated and then tissue pieces were transferred to individual Beem capsules filled with fresh resin and polymerized for 48 h at 60°C. Once polymerized, blocks were sectioned using a Diatome diamond knife on a Leica UC7 Ultramicrotome. Ultrathin (90 nm) sections were transferred onto 200 mesh copper grids and then post‐stained with lead citrate for 5 min, washed and air dried. Grids were imaged with a Thermo Fisher Tecnai 12 TEM (operated at 120 kV) using a Gatan OneView camera.

### Extracellular cytokine measurements

Serum samples were collected by cardiac puncture and collected in Microtainer tubes (365978, BD Bioscience). Samples were centrifuged for 90 s at 15,000 *g* and serum aliquots were snap‐frozen until further analysis. Global inflammatory cytokine analysis of supernatants of adipose tissue explant cultures and serum were performed using LEGENDPlex™ Mouse Inflammation Panel (740446, Biolegend). Supernatant IL‐10 levels were measured by IL‐10 Mouse Uncoated ELISA Kit (88‐7105‐86, Invitrogen). Adipose tissue‐derived cytokine levels were normalized to input tissue weight.

### Statistical analysis

Data were tested for eventual statistical outliers (ROUT analysis with a *Q* = 1%) and outliers were removed if detected. Next, data were tested normality before applying parametric or nonparametric testing. For two normally distributed groups, unpaired Student's tests were applied. Comparisons across more than two experimental groups were performed using one‐way or two‐way ANOVA with Šídák multiple testing correction. Data were considered statistically significant when *P* < 0.05 (*P < 0.05, **P < 0.01, ***P < 0.001, ****P < 0.0001). Typically, data were pooled from or representative of at least two experiments, if not otherwise indicated, and presented as mean. Data were visualized and statistics calculated in either GraphPad Prism 9 or R software.

## Author contributions

**Felix Clemens Richter:** Conceptualization; formal analysis; funding acquisition; investigation; visualization; methodology; writing – original draft; writing – review and editing. **Matthias Friedrich:** Conceptualization; formal analysis; supervision; investigation; methodology; writing – review and editing. **Nadja Kampschulte:** Formal analysis; investigation; methodology; writing – review and editing. **Klara Piletic:** Investigation; writing – review and editing. **Ghada Alsaleh:** Conceptualization; investigation; writing – review and editing. **Ramona Zummach:** Investigation. **Julia Hecker:** Resources; investigation. **Mathilde Pohin:** Conceptualization; investigation; writing – review and editing. **Nicholas Ilott:** Data curation; formal analysis. **Irina Guschina:** Investigation; methodology. **Sarah Karin Wideman:** Investigation; writing – review and editing. **Errin Johnson:** Investigation; methodology; writing – review and editing. **Mariana Borsa:** Investigation; writing – review and editing. **Paula Hahn:** Investigation; writing – review and editing. **Christophe Morriseau:** Resources; methodology. **Bruce D Hammock:** Conceptualization; resources. **Henk Simon Schipper:** Conceptualization; supervision; writing – review and editing. **Claire M Edwards:** Conceptualization; supervision; writing – review and editing. **Rudolf Zechner:** Resources; methodology. **Britta Siegmund:** Conceptualization; resources. **Carl Weidinger:** Conceptualization; resources; investigation. **Nils Helge Schebb:** Conceptualization; formal analysis; supervision; investigation; methodology; writing – review and editing. **Fiona Powrie:** Conceptualization; supervision; funding acquisition; writing – review and editing. **Anna Katharina Simon:** Conceptualization; supervision; funding acquisition; methodology; writing – original draft; writing – review and editing.

## Disclosure and competing interests statement

BDH is founder of EicOsis Human Health developing sEH inhibitors as human pharmaceuticals. FP received research support or consultancy fees from Roche, Janssen, GSK, Novartis and Genentech. AKS received consultancy fees from Calico, Oxford Healthspan, The Longevity Lab.

## Supporting information



Appendix

Expanded View Figures PDF

Source Data for Expanded View and Appendix

PDF+

Source Data for Figure 1

Source Data for Figure 2

Source Data for Figure 3

Source Data for Figure 4

Source Data for Figure 5

Source Data for Figure 6

Source Data for Figure 7

## Data Availability

All reagents used in this study are commercially available. Source data underlying the graphs as well as representative western blots have been made available in the Source Data file. Transcriptomic dataset is available on EBI ArrayExpress (Access Code: E‐MTAB‐12498). In addition, RNAseq analysis R scripts can be found on https://github.com/cleete/IBD‐Adipocyte‐Autophagy.
