# Supplementary material for: Adipocyte autophagy limits gut inflammation by controlling oxylipin and IL‐10
Source: EMBO J. 2023 Feb 16;42(6):e112202. doi: 10.15252/embj.2022112202 (PMC10015370; doi:10.15252/embj.2022112202)

Table of Content

Appendix Figure S1

Figure Legend

Appendix Figure S1: Adipocyte autophagy loss results in shifts of macrophage polarization.
(A) Frequency of adipose tissue macrophages among all immune cells in adipose tissues (n = 7-9/group).
(B) Expression of CD206 on adipose tissue macrophages in visceral adipose tissues (n = 6-12/group).
(C) Expression of CD36 on adipose tissue macrophages. Dotted line represents uninflamed controls (n = 5-7/group).

Data are represented as mean ± s.e.m. (A-C) Unpaired Student's t-test.

Appendix Figure S1

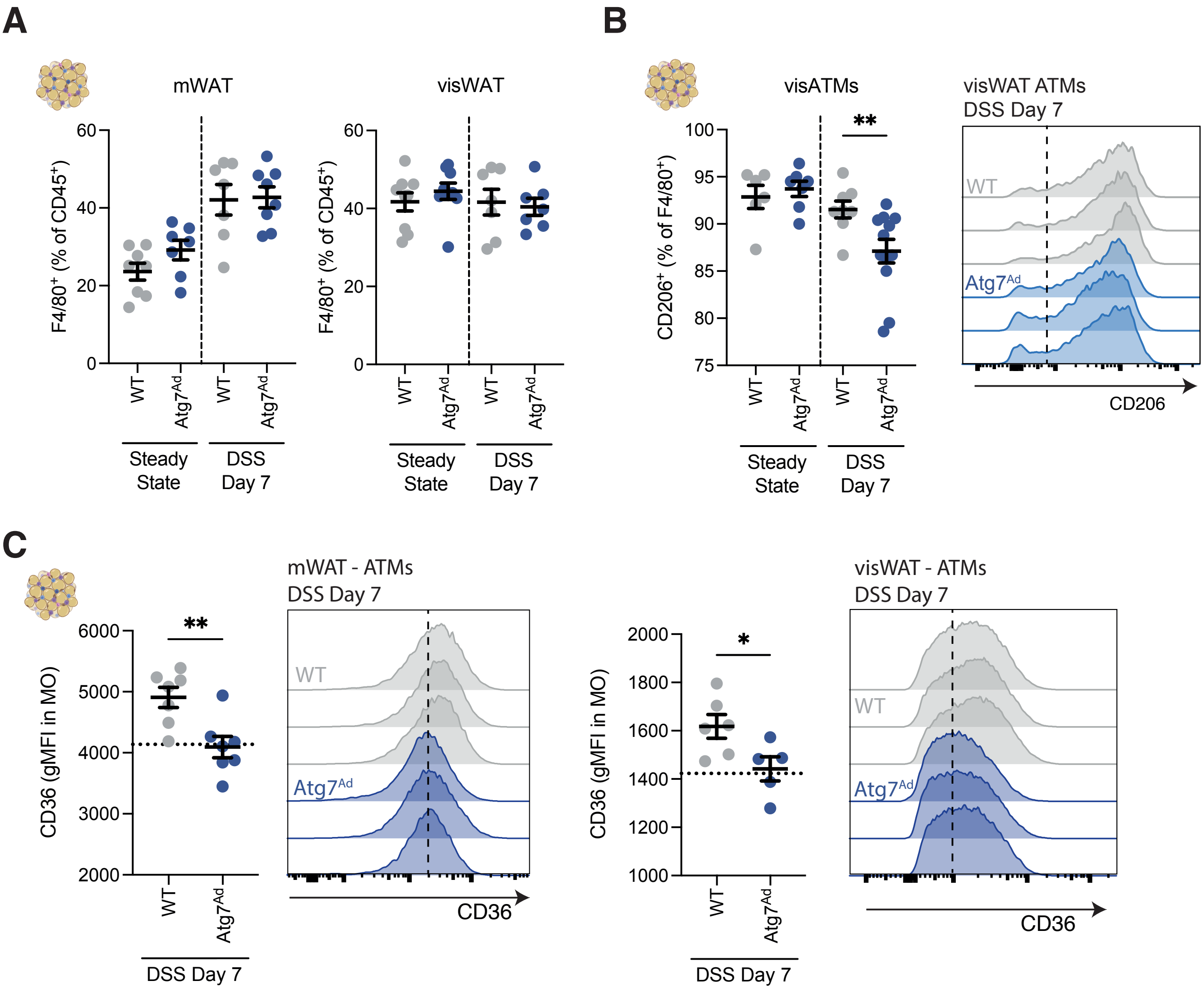

Supplement: Supplementary file 1 — Appendix [file EMBJ-42-e112202-s007.pdf]
