# Supplementary material for: Adipocyte autophagy limits gut inflammation by controlling oxylipin and IL‐10
Source: EMBO J. 2023 Feb 16;42(6):e112202. doi: 10.15252/embj.2022112202 (PMC10015370; doi:10.15252/embj.2022112202)
Supplement: Supplementary file 2 — Expanded View Figures PDF [file EMBJ-42-e112202-s004.pdf]

Expanded View Figures

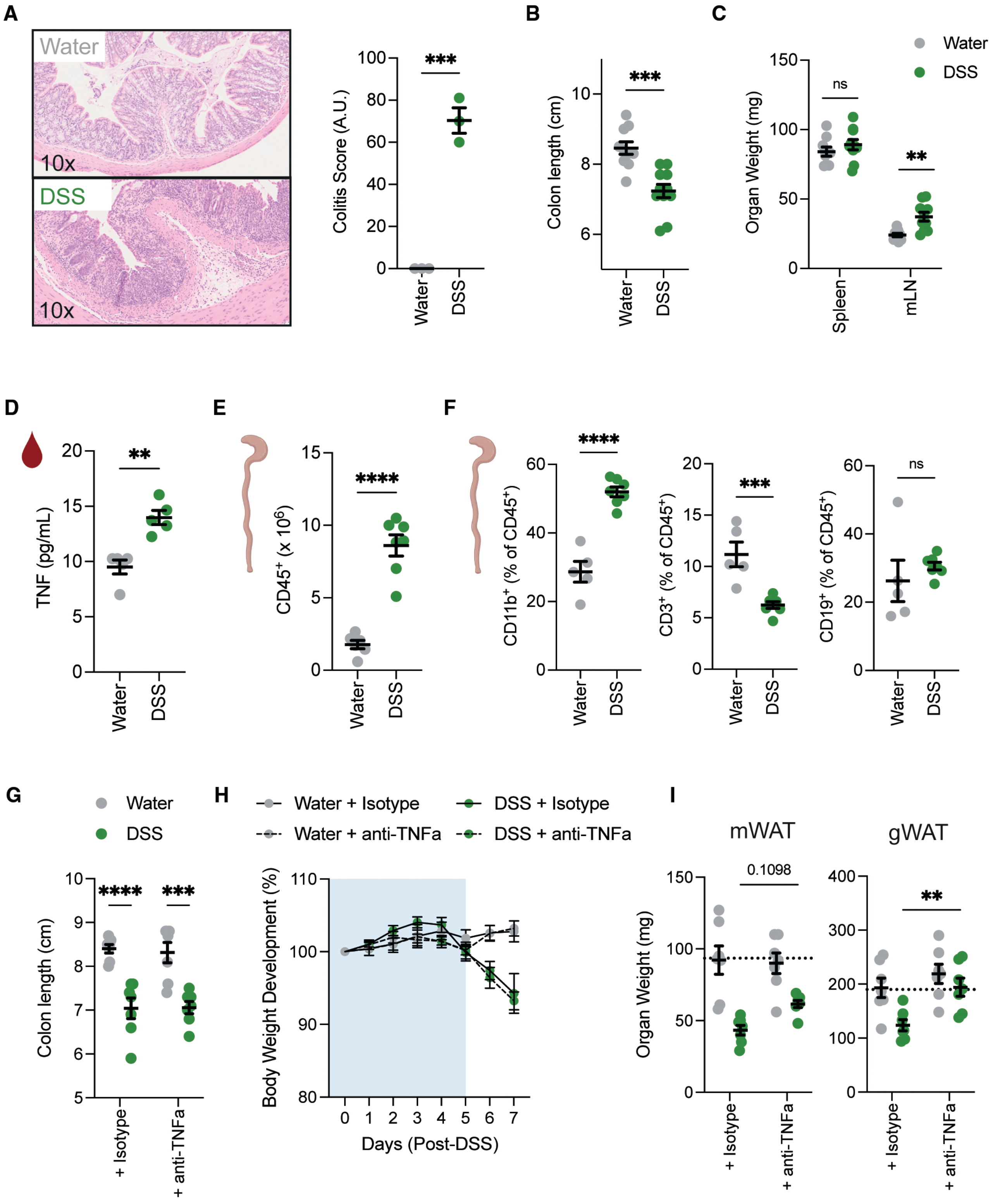

Figure EV1.

Figure EV1. DSS leads to efficient induction of intestinal inflammation.

A Representative H&E staining of colon histology and quantification on day 7 after DSS colitis induction ($n = 3$/group) from one independent experiment.
B Colon length measured after 1.5–2% DSS colitis regime on day 7 ($n = 10$–11/group).
C Spleen weight and mesenteric lymph node weight after 1.5–2% colitis regime on day 7 ($n = 9$–10/group).
D TNFα levels in serum were measured in wild-type mice on day 7 after water and DSS treatment ($n = 5$/group).
E Absolute number of colonic $CD45^+$ immune cells on day 7 post-DSS treatment ($n = 6$–7/group).
F Frequency of $CD11b^+$ myeloid cells, $CD3^+$ T cells and $CD19^+$ B cells in colon on day 7 post-DSS treatment ($n = 5$–7/group).
G Colon length of mice upon DSS-induced colitis treated with anti-isotype or anti-TNFα neutralizing antibody ($n = 7$/group).
H Body weight development upon DSS-induced colitis of mice treated either with anti-isotype or anti-TNFα neutralizing antibody ($n = 7$/group).
I Tissue weight of mWAT or gWAT upon DSS-induced colitis of mice treated either with anti-isotype or anti-TNFα neutralizing antibody ($n = 7$/group).

Data are represented as mean ± s.e.m. (A–E) Unpaired Student's t-test. (G, I) Two-way ANOVA. (H) Repeat-measure two-way ANOVA. $^{**}P < 0.01$, $^{***}P < 0.001$, $^{****}P < 0.0001$.
Source data are available online for this figure.

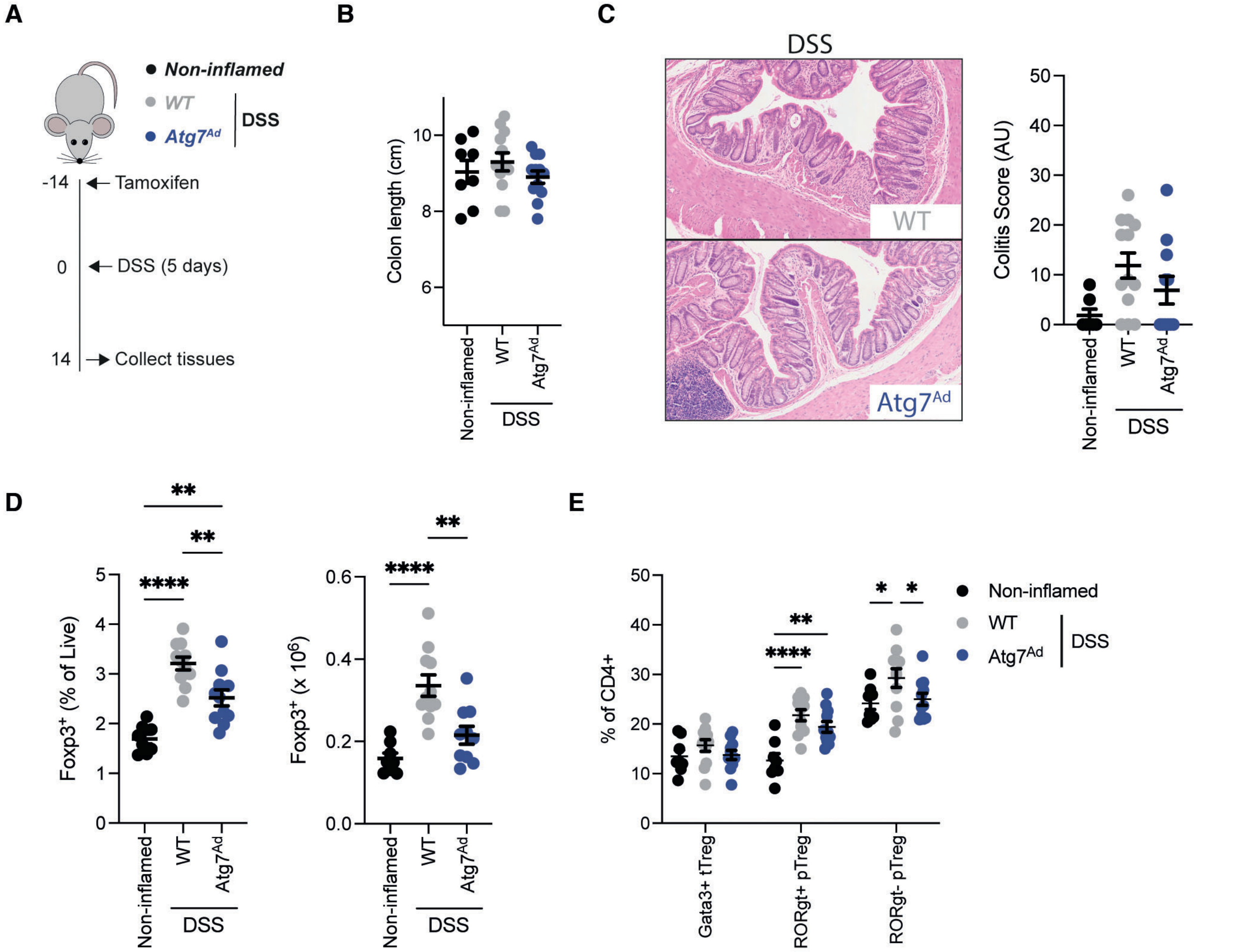

Figure EV2.

 © 2023 The Authors

Figure EV2. Expansion of intestinal Treg populations is blunted in adipocyte autophagy-deficient mice without affecting intestinal resolution.

A Schematic of experimental design. Sex-matched and age-matched littermates were treated with DSS for 5 days and mice were sacrificed 14 days after start of DSS treatment.

B Colon length from noninflamed control mice (n = 8/group), adipocyte autophagy-sufficient WT mice and adipocyte autophagy-deficient mice (n = 12/group).

C Representative H&E staining images (10× magnification) of distal colon sections and quantification of histopathological score (n = 7–13/group).

D Frequency (left panel) and absolute number (right panel) of $CD4^+$ $FOXP3^+$ cells in the colon on day 14 post-DSS treatment (n = 8–11/group).

E Frequency of peripheral and thymic Treg (pTreg and tTreg, respectively) cell populations in colon on day 14 post-DSS treatment (n = 8–11/group).

Data are represented as mean ± s.e.m. (B–D) One-way ANOVA. (E) Two-way ANOVA. *$P < 0.05$, **$P < 0.01$, ****$P < 0.0001$.
Source data are available online for this figure.

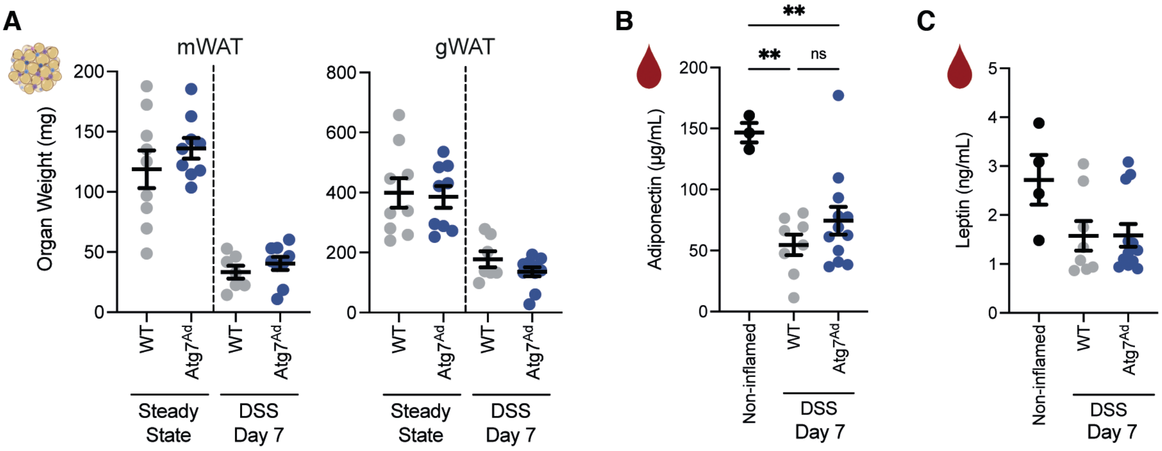

Figure EV3. Loss of adipocyte autophagy had no effects on adipose tissue and circulating levels of leptin and adiponectin.

A Adipose tissue mass at steady state and on day 7 post-DSS induction (n = 7–11/group).

B Circulating levels of adiponectin (n = 3–12/group).

C Circulating levels of leptin (n = 4–12/group).

Data are represented as mean ± s.e.m. (A) Unpaired Student's t-test. (B, C) One-way ANOVA. **$P < 0.01$.
Source data are available online for this figure.

© 2023 The Authors

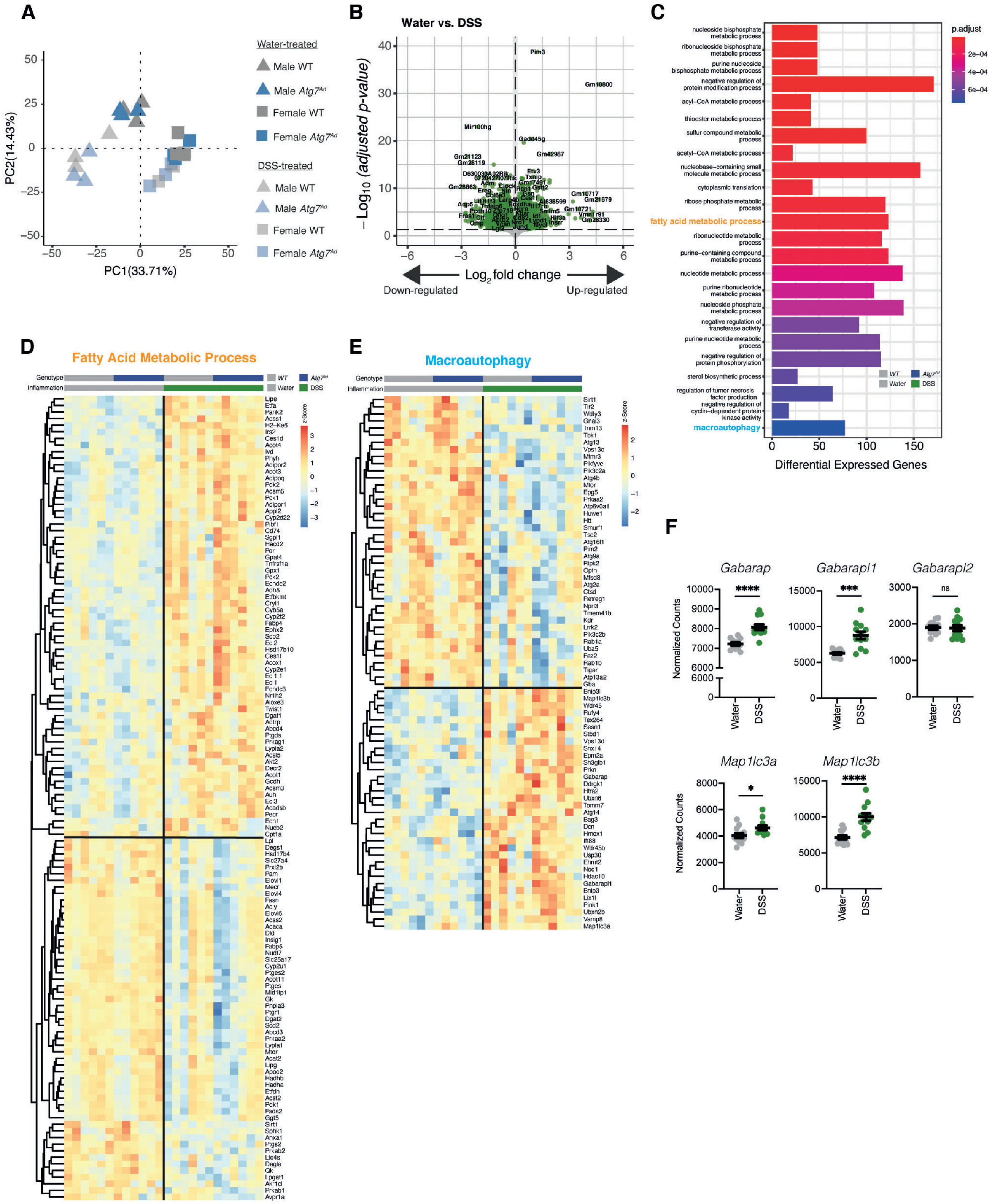

Figure EV4.

 © 2023 The Authors

Figure EV4. Intestinal inflammation induces distinct transcriptional programs in primary visceral adipocytes.

A Principal component analysis of all mice revealing a strong sex effect in the overall transcriptome.
B Differential gene expression assessing transcriptional changes associated with DSS-induced inflammation after regressing effect of sex and genotypes in visceral adipocytes.
C Pathway enrichment analysis of significantly differentially expressed genes in visceral adipocytes during DSS colitis.
D Heatmap representing differentially expressed genes associated in fatty acid metabolism during DSS-induced colitis in visceral adipocytes.
E Heatmap representing differentially expressed genes associated with macroautophagy during DSS-induced colitis in visceral adipocytes.
F Normalized counts of *Atg8* homologs in visceral adipocytes ($n = 12$/group).

Data are represented as mean ± s.e.m. (F) Unpaired Student's *t*-test. *$P < 0.05$, ***$P < 0.001$
Source data are available online for this figure.

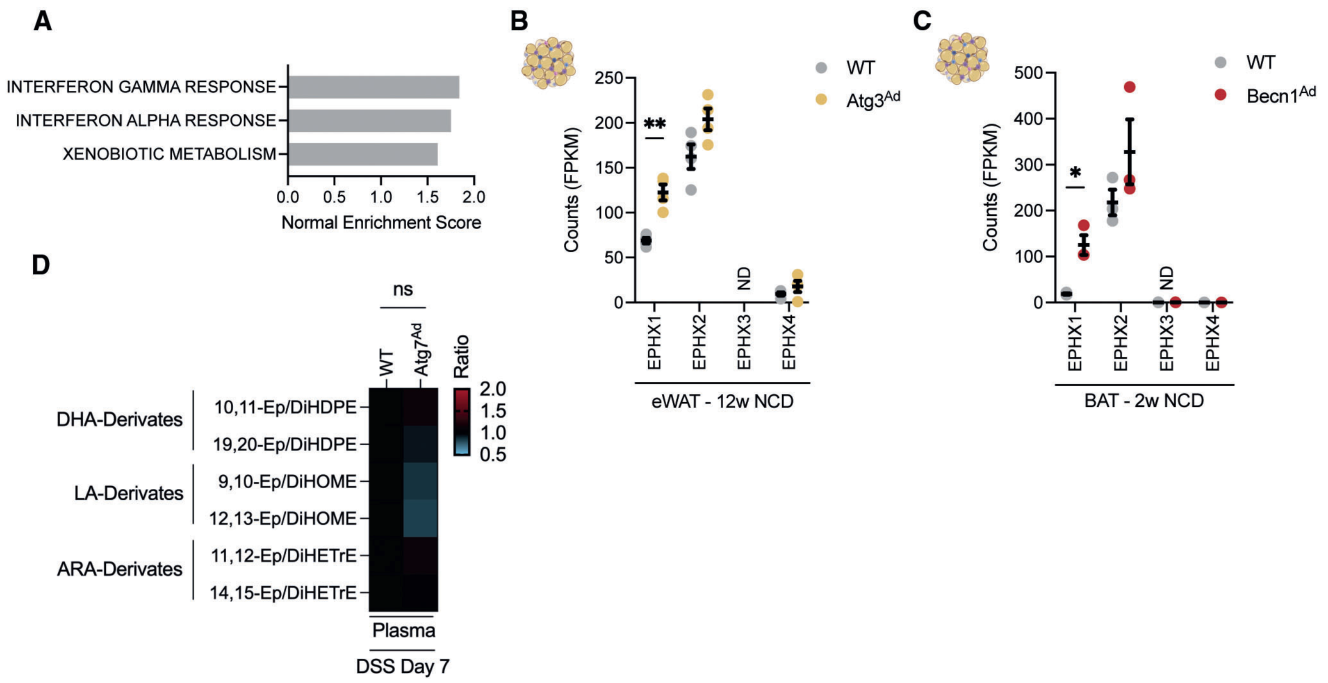

Figure EV5. Loss of autophagy-related genes results in the induction of epoxy hydrolases in adipocytes.

A GSEA enrichment analysis between *Atg7*-deficient and *Atg7*-sufficient adipocytes during DSS treatment.
B Fragments per kilobase of exon per million mapped fragments (FPKM) counts from bulk RNAseq dataset of Cai *et al* (2018) ($n = 4$/group)
C Fragments per kilobase of exon per million mapped fragments (FPKM) counts from bulk RNAseq dataset of Son *et al* (2020) ($n = 3$/group).
D Normalized ratios of epoxy fatty acid precursors to their corresponding diol fatty acids pairs in plasma ($n = 8$/group).

Data are represented as mean ± s.e.m. (B, C) Unpaired Student's *t*-test. (D) Two-way ANOVA. *$P < 0.05$, **$P < 0.01$.
Source data are available online for this figure.

© 2023 The Authors
