# Supplementary figures and images for: Adipocyte autophagy limits gut inflammation by controlling oxylipin and IL‐10
Source: EMBO J. 2023 Feb 16;42(6):e112202. doi: 10.15252/embj.2022112202 (PMC10015370; doi:10.15252/embj.2022112202)

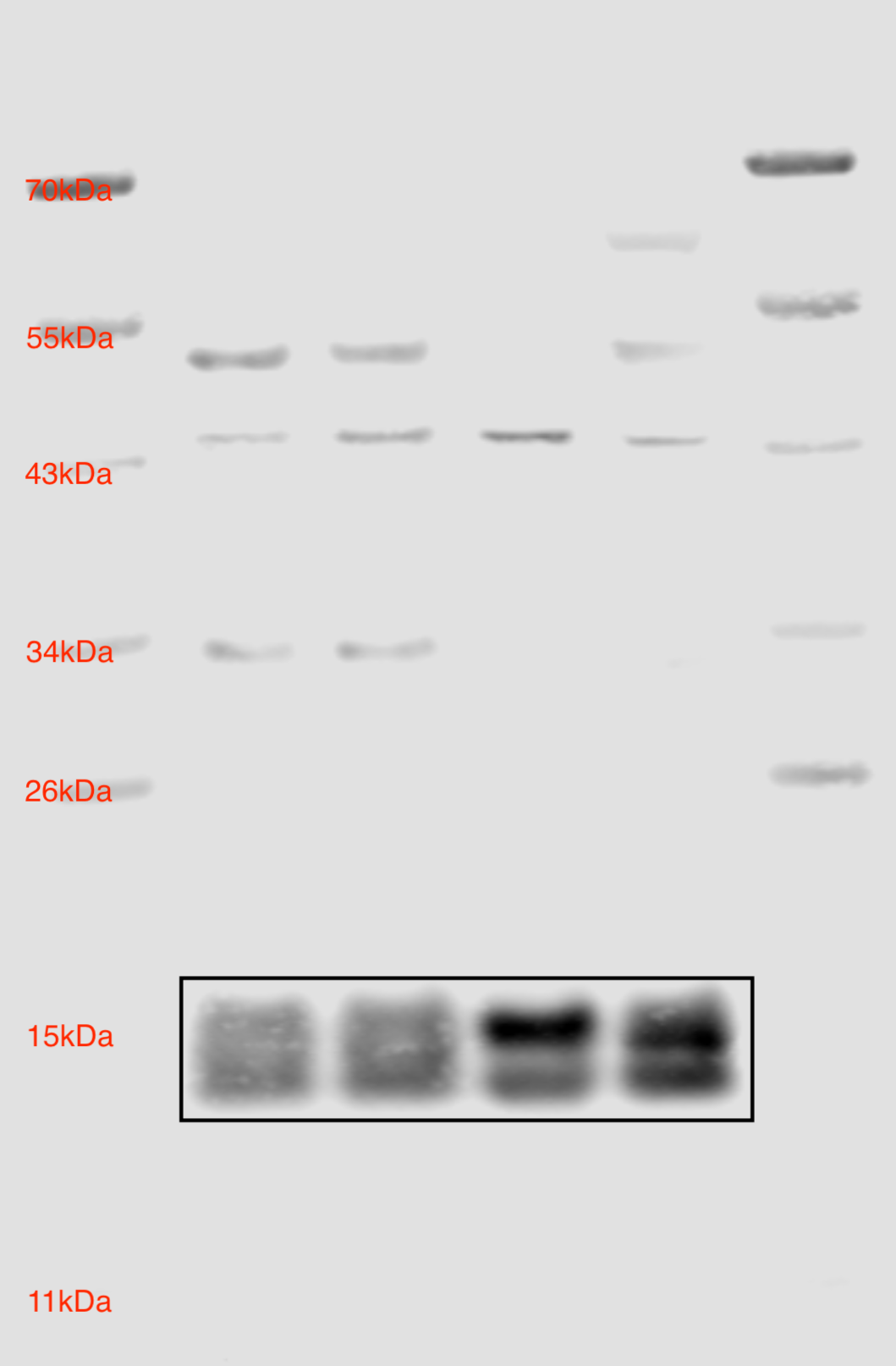

Supplement: Supplementary file 5 — Source Data for Figure 1 [file EMBJ-42-e112202-s008.zip › Figure 1/1F/mWAT LC3.tif]

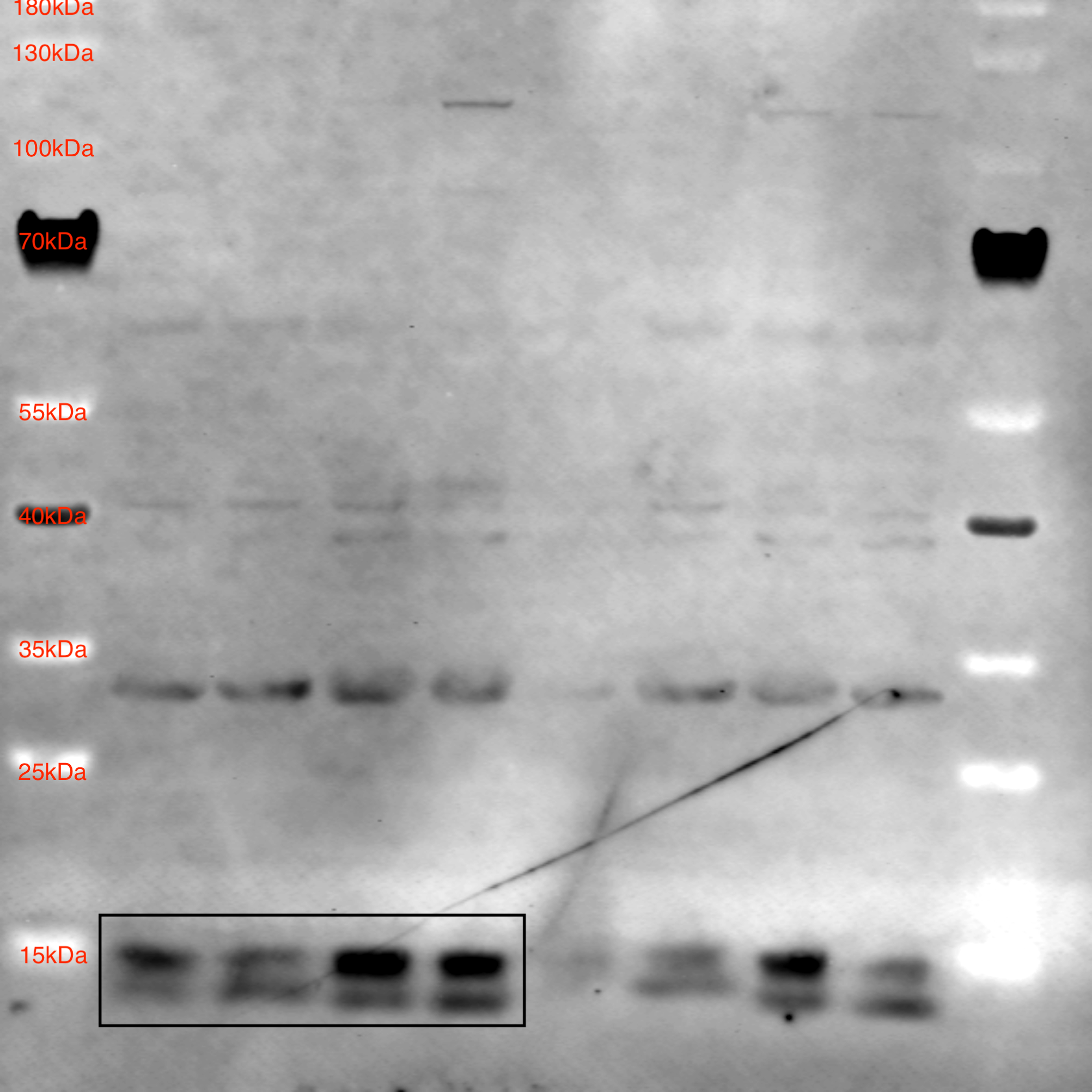

Supplement: Supplementary file 5 — Source Data for Figure 1 [file EMBJ-42-e112202-s008.zip › Figure 1/1F/gWAT LC3.tif]

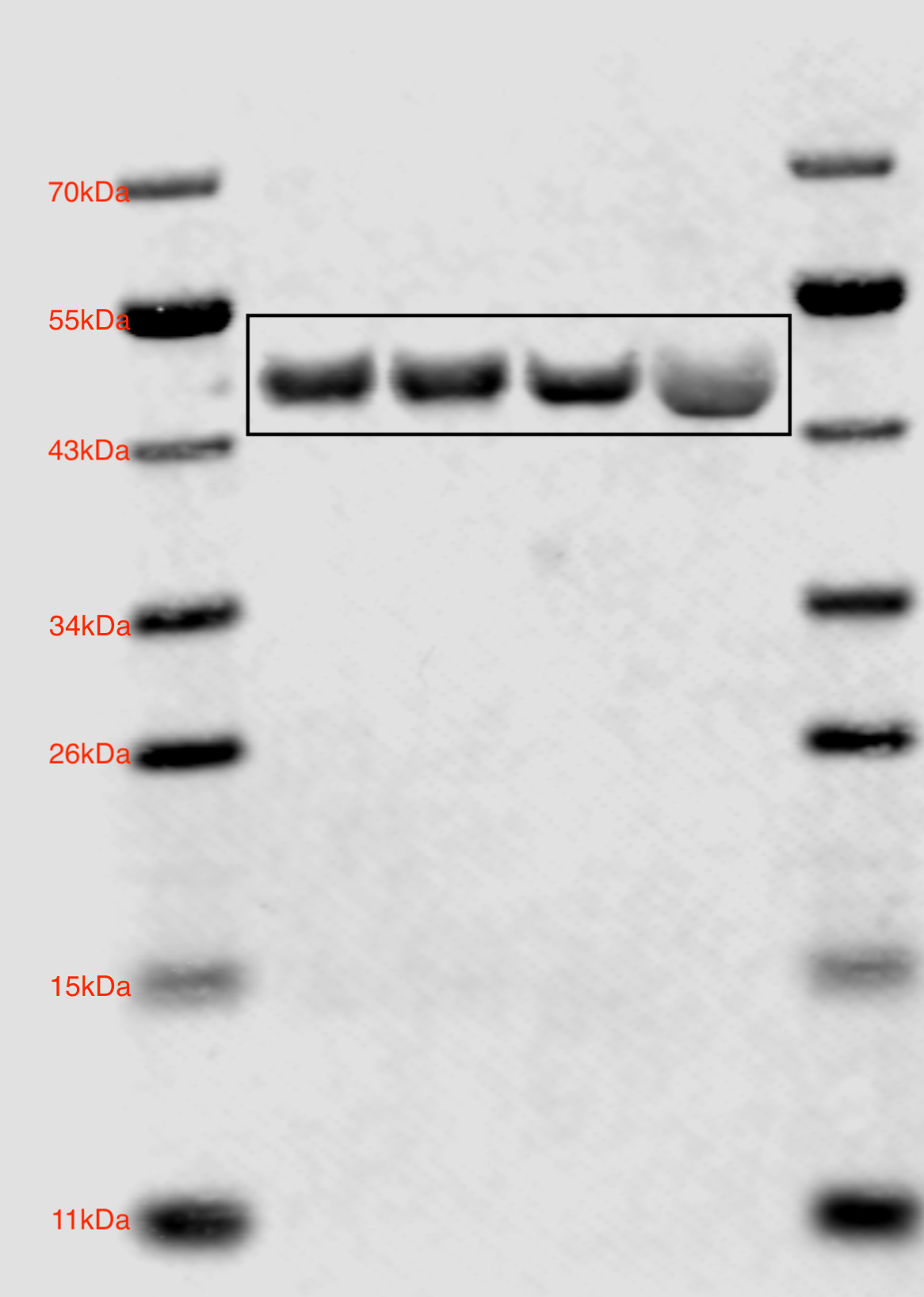

Supplement: Supplementary file 5 — Source Data for Figure 1 [file EMBJ-42-e112202-s008.zip › Figure 1/1F/mWAT Actin.tif]

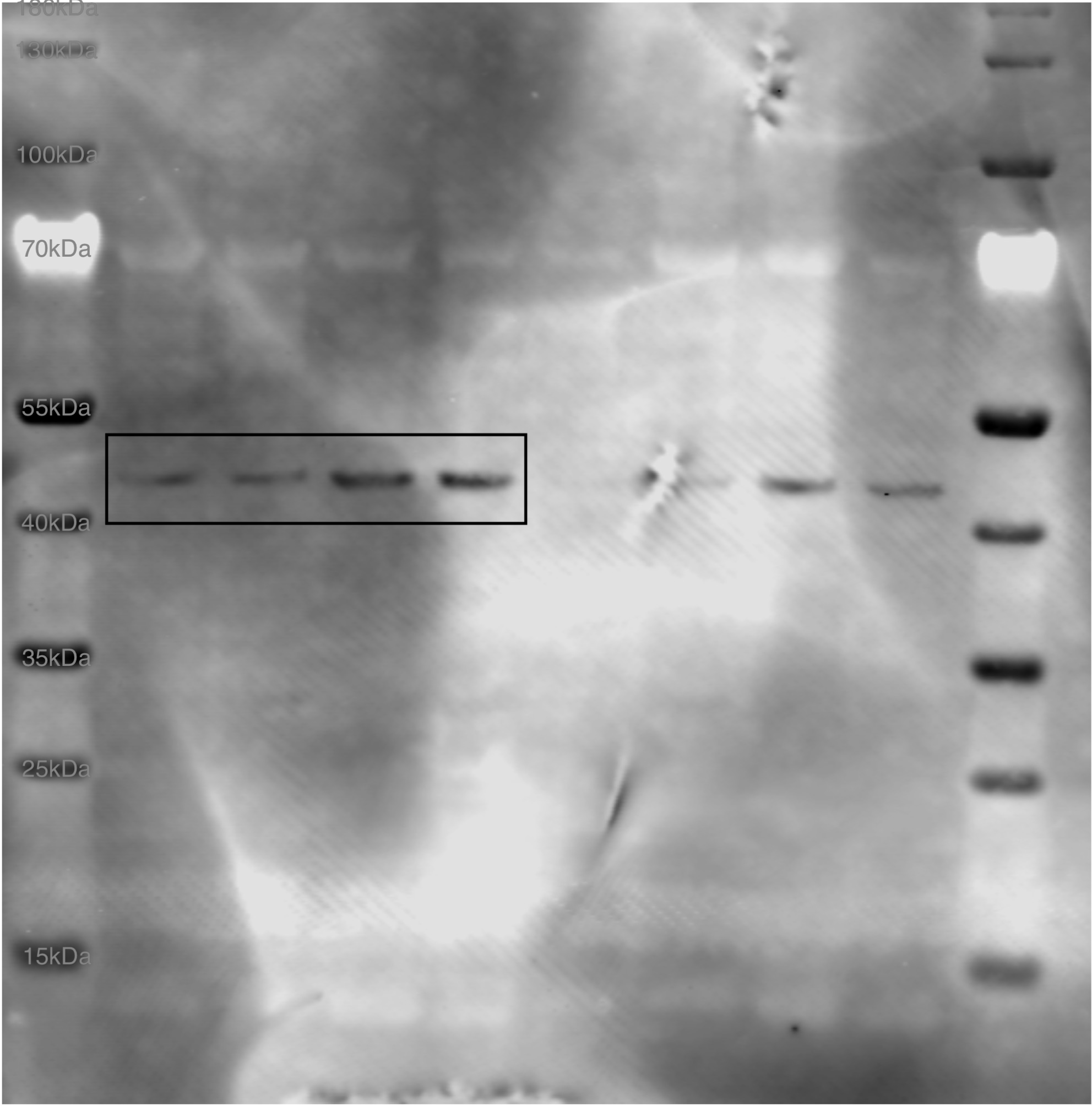

Supplement: Supplementary file 5 — Source Data for Figure 1 [file EMBJ-42-e112202-s008.zip › Figure 1/1F/gWAT Actin.tif]

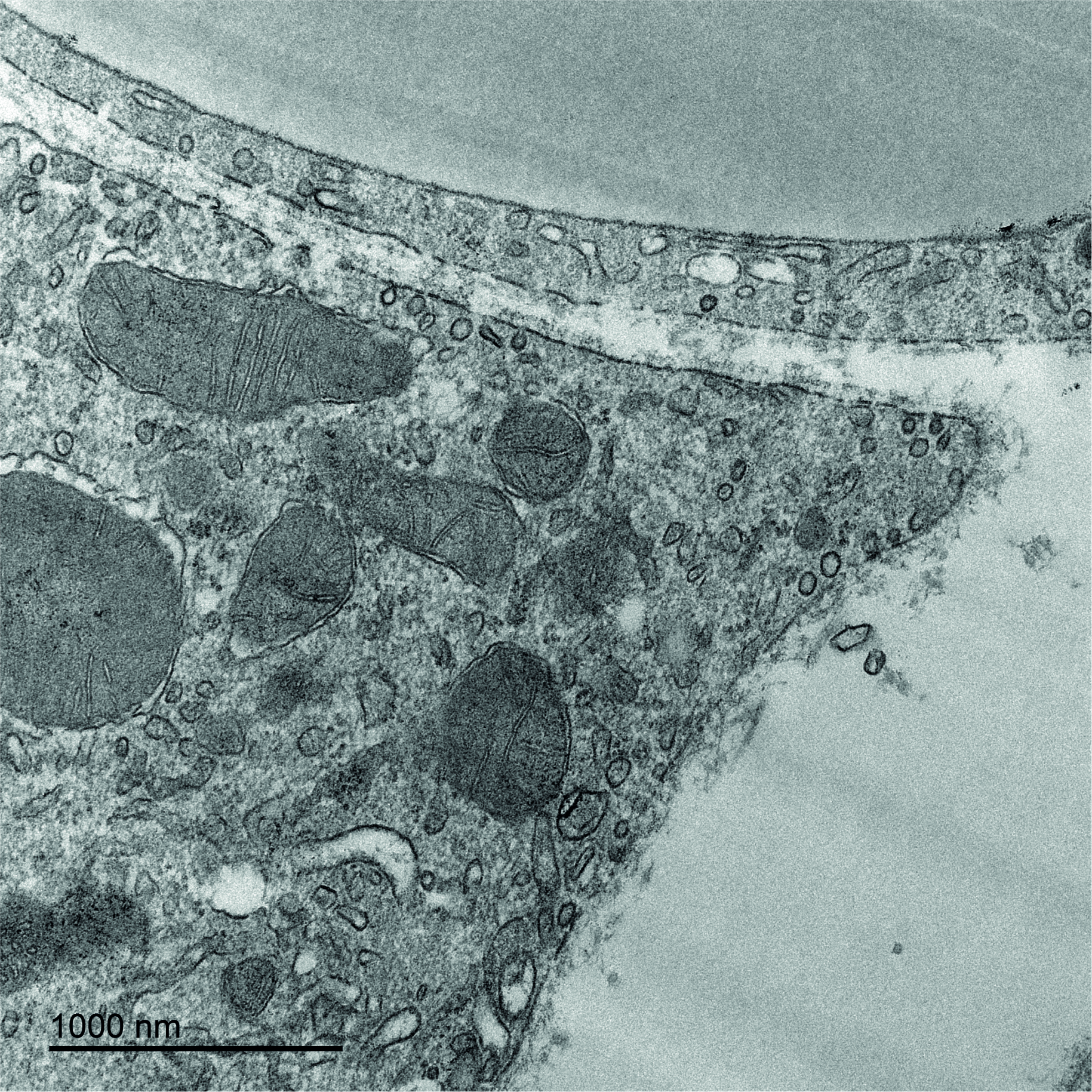

Supplement: Supplementary file 5 — Source Data for Figure 1 [file EMBJ-42-e112202-s008.zip › Figure 1/1G/Water mWAT.tif]

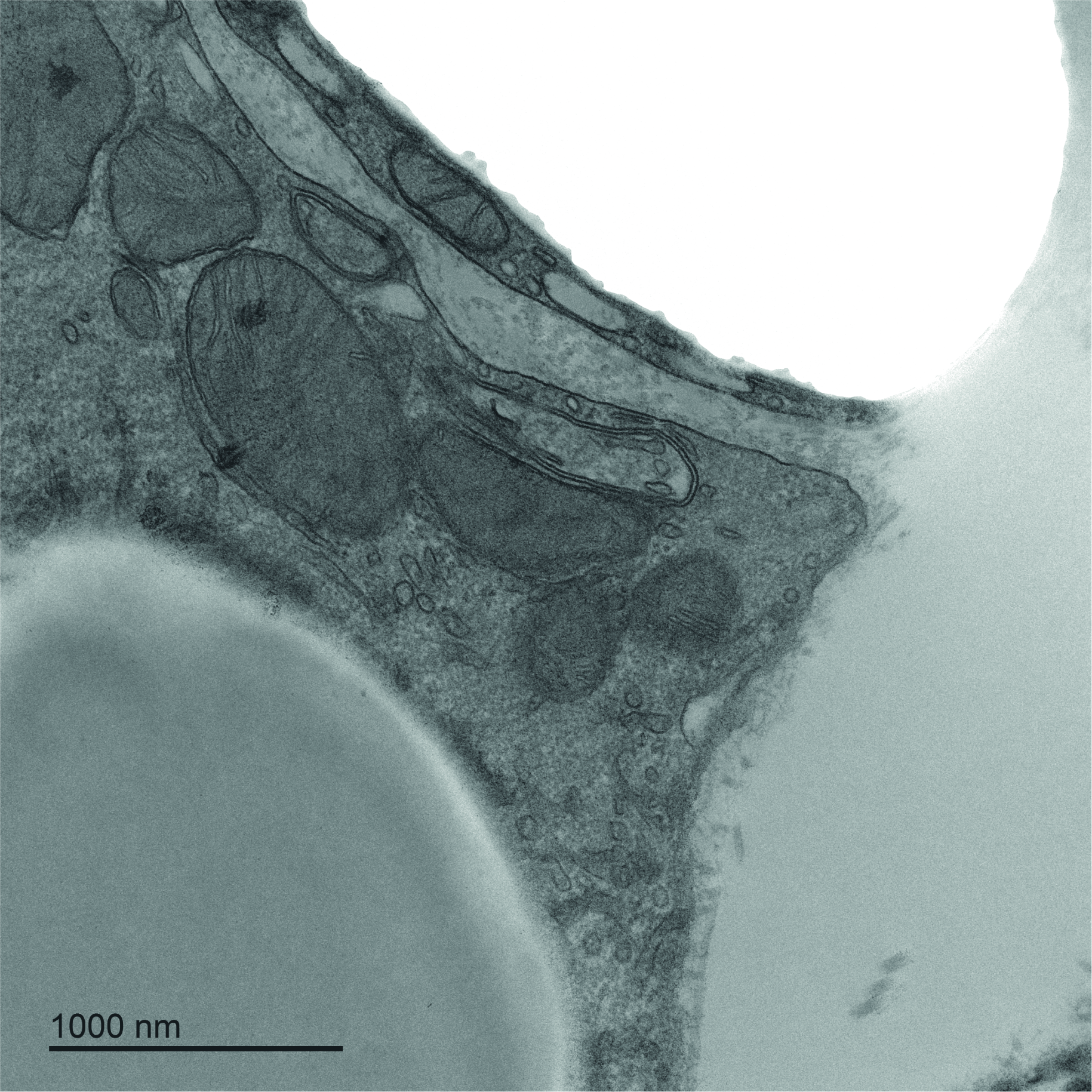

Supplement: Supplementary file 5 — Source Data for Figure 1 [file EMBJ-42-e112202-s008.zip › Figure 1/1G/DSS mWAT.tif]

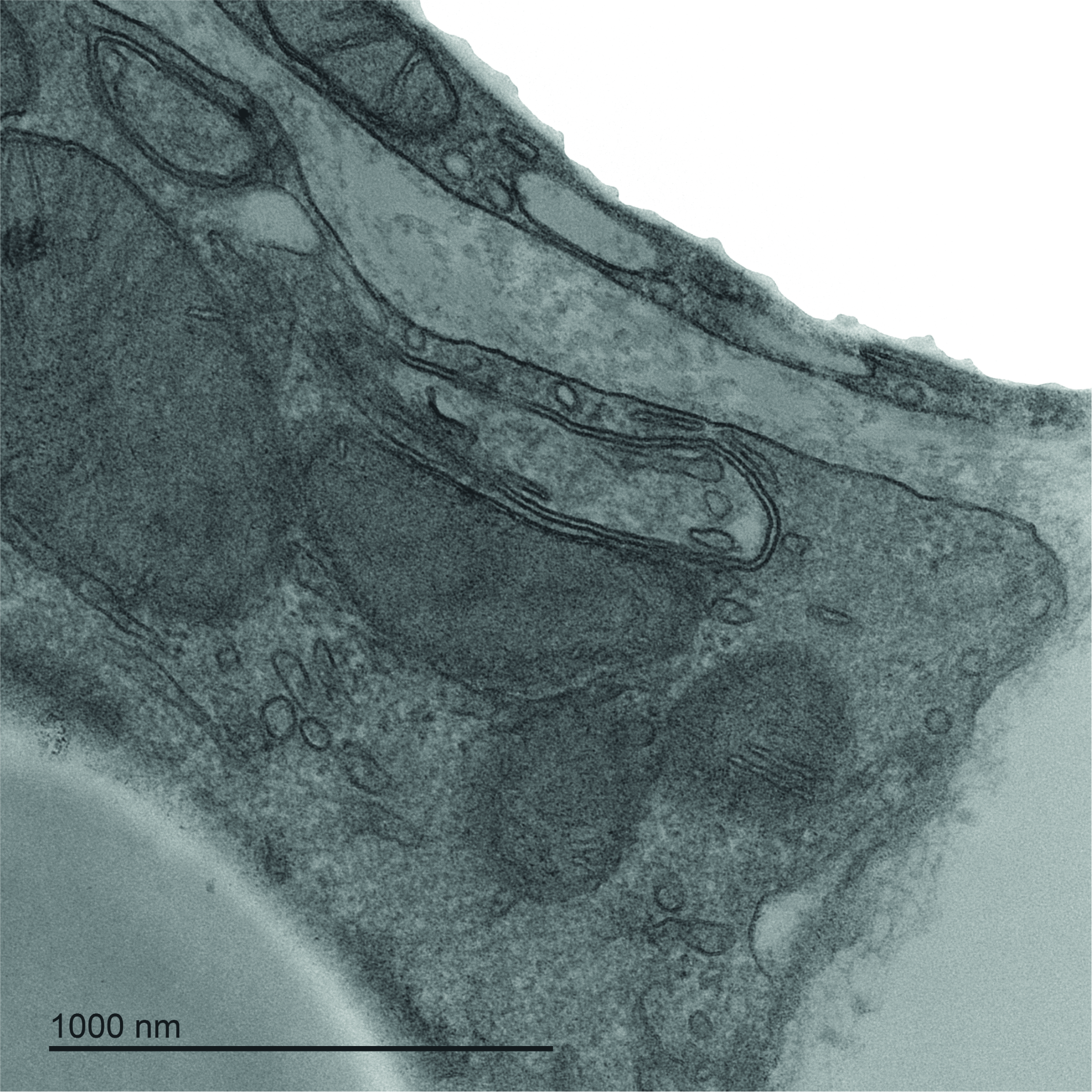

Supplement: Supplementary file 5 — Source Data for Figure 1 [file EMBJ-42-e112202-s008.zip › Figure 1/1G/DSS mWAT-zoom.tif]

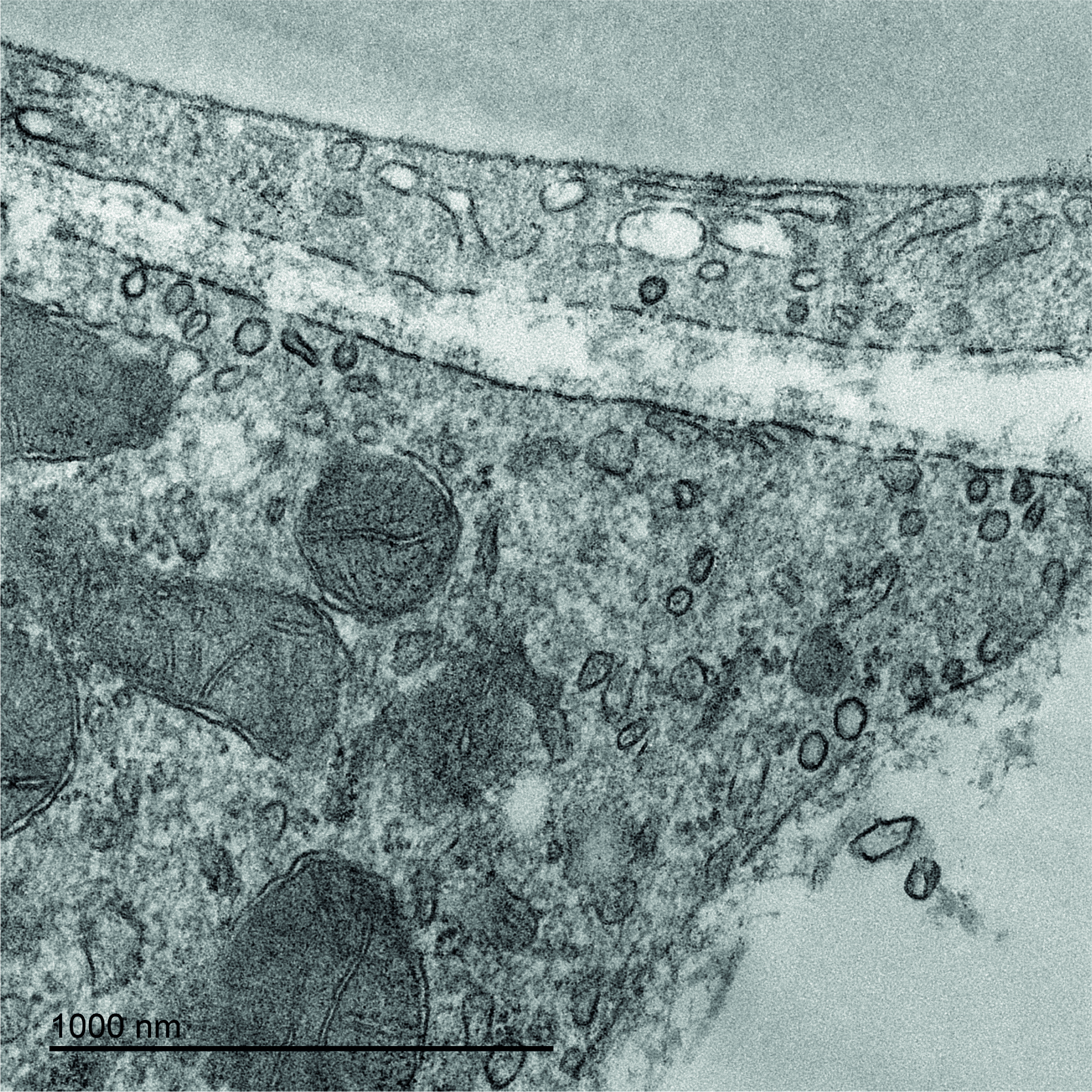

Supplement: Supplementary file 5 — Source Data for Figure 1 [file EMBJ-42-e112202-s008.zip › Figure 1/1G/Water mWAT-zoom.tif]

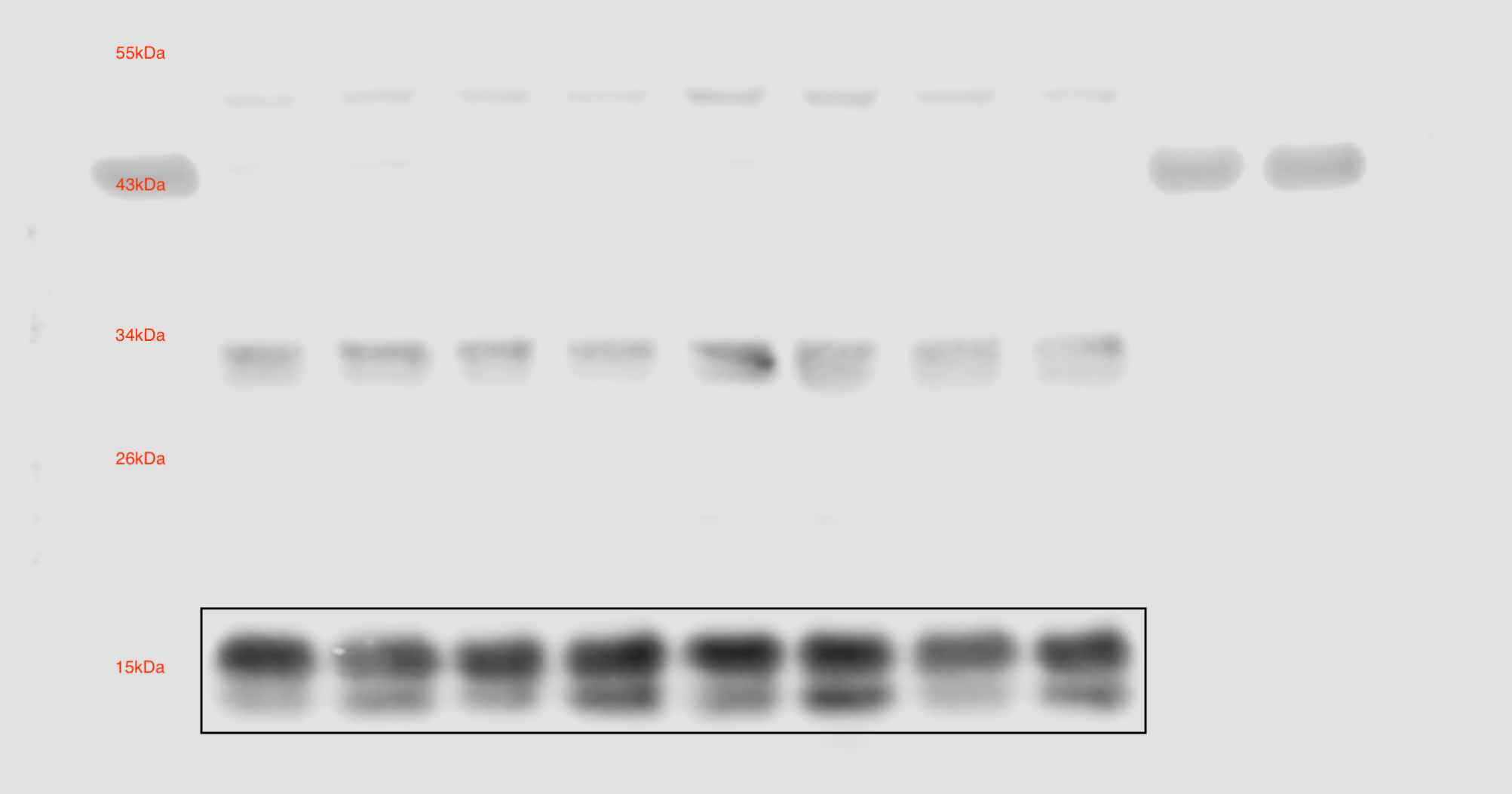

Supplement: Supplementary file 5 — Source Data for Figure 1 [file EMBJ-42-e112202-s008.zip › Figure 1/1I/gWAT_anti-TNF_LC3.tif]

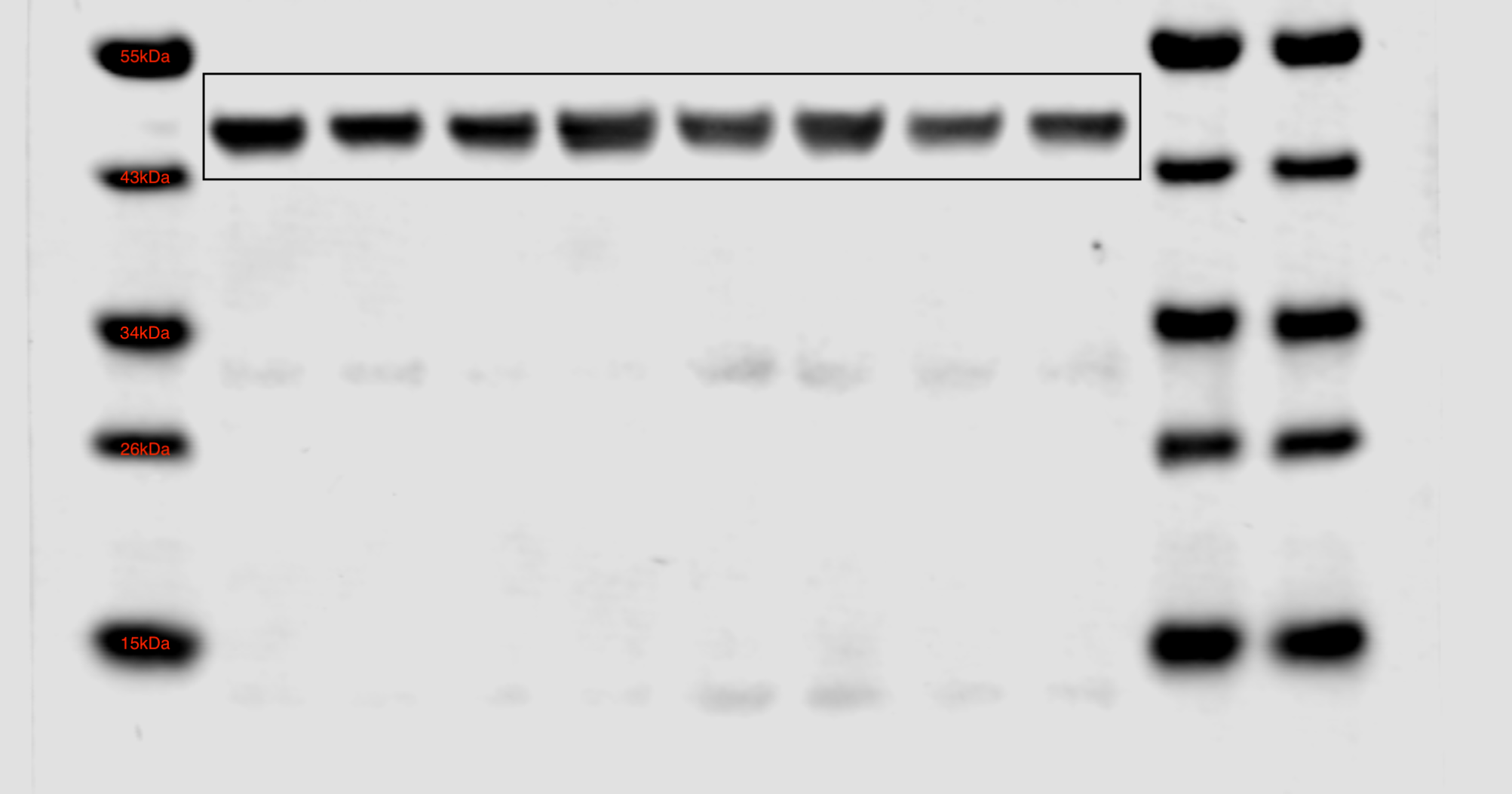

Supplement: Supplementary file 5 — Source Data for Figure 1 [file EMBJ-42-e112202-s008.zip › Figure 1/1I/gWAT_anti-TNF_Actin.tif]

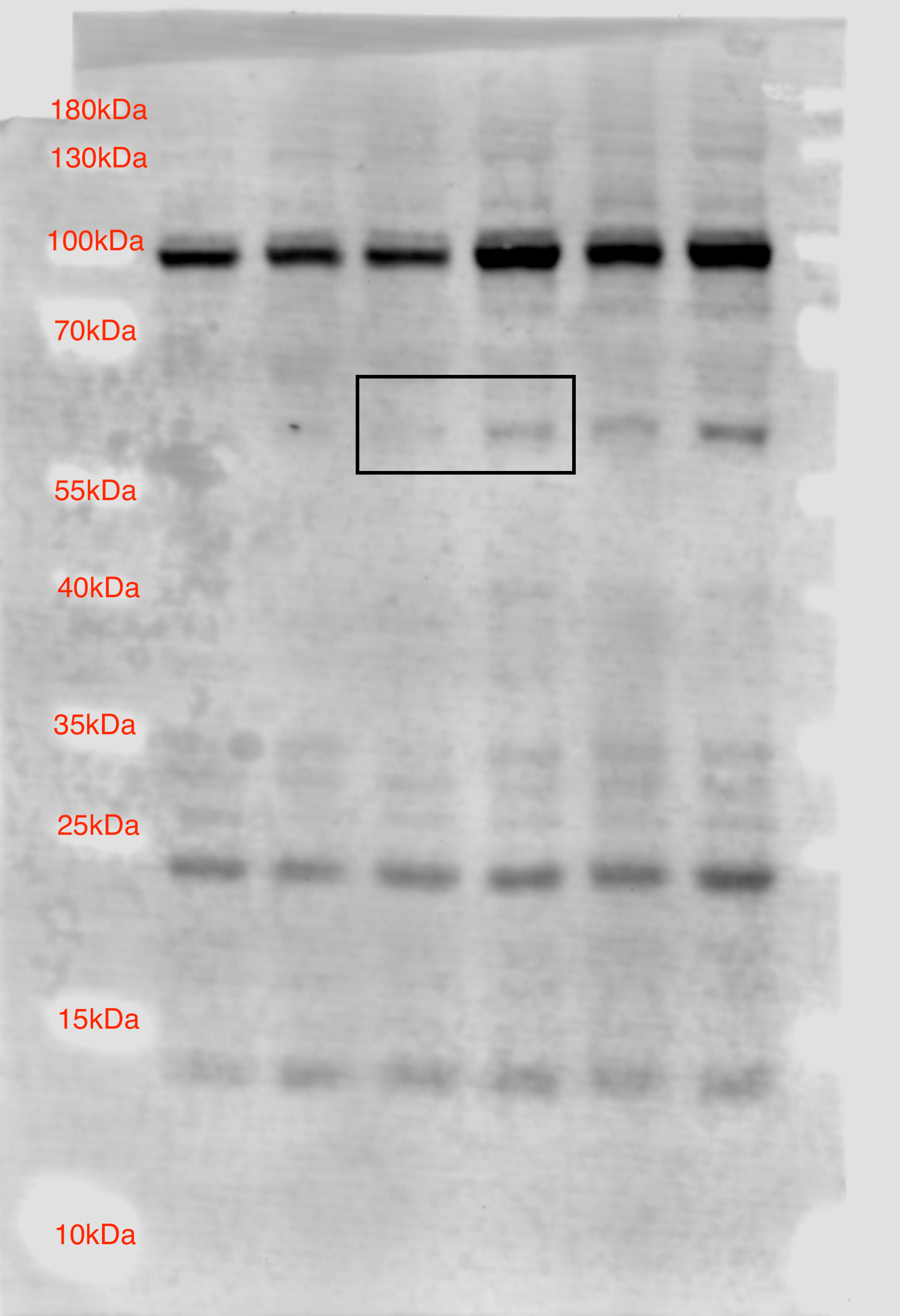

Supplement: Supplementary file 5 — Source Data for Figure 1 [file EMBJ-42-e112202-s008.zip › Figure 1/1E/Atgl BW.tif]

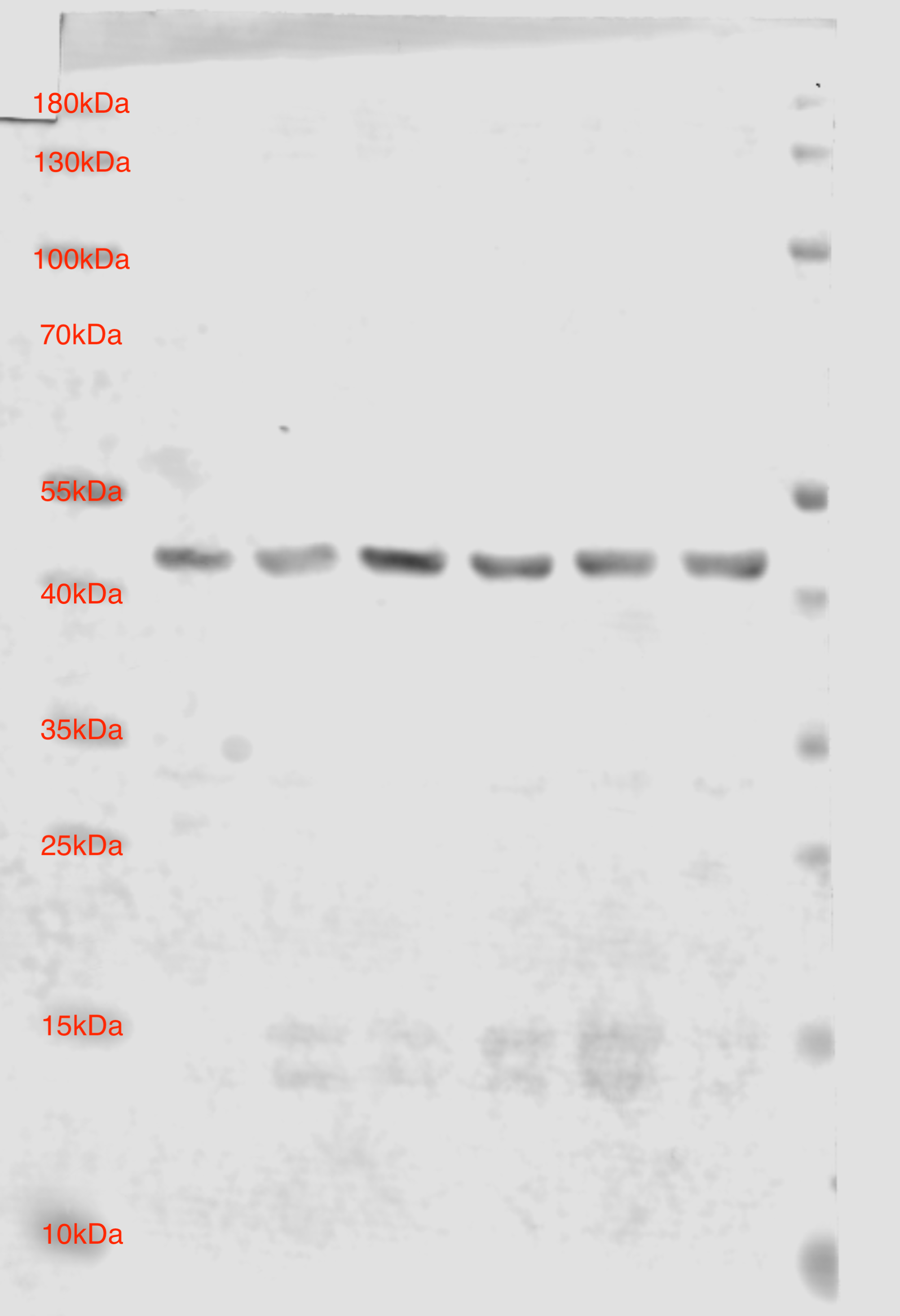

Supplement: Supplementary file 5 — Source Data for Figure 1 [file EMBJ-42-e112202-s008.zip › Figure 1/1E/Actin BW.tif]

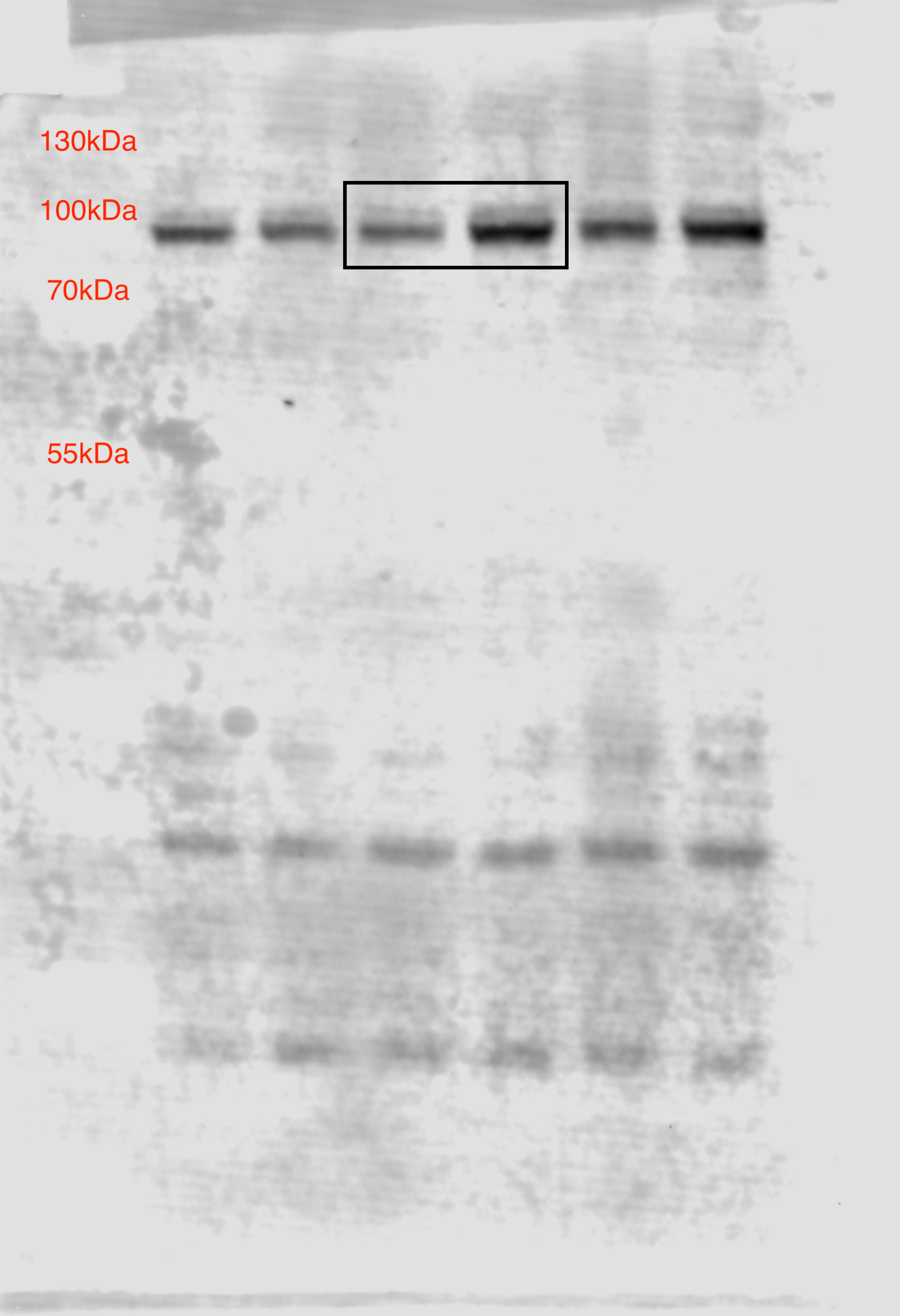

Supplement: Supplementary file 5 — Source Data for Figure 1 [file EMBJ-42-e112202-s008.zip › Figure 1/1E/tHSL BW.tif]

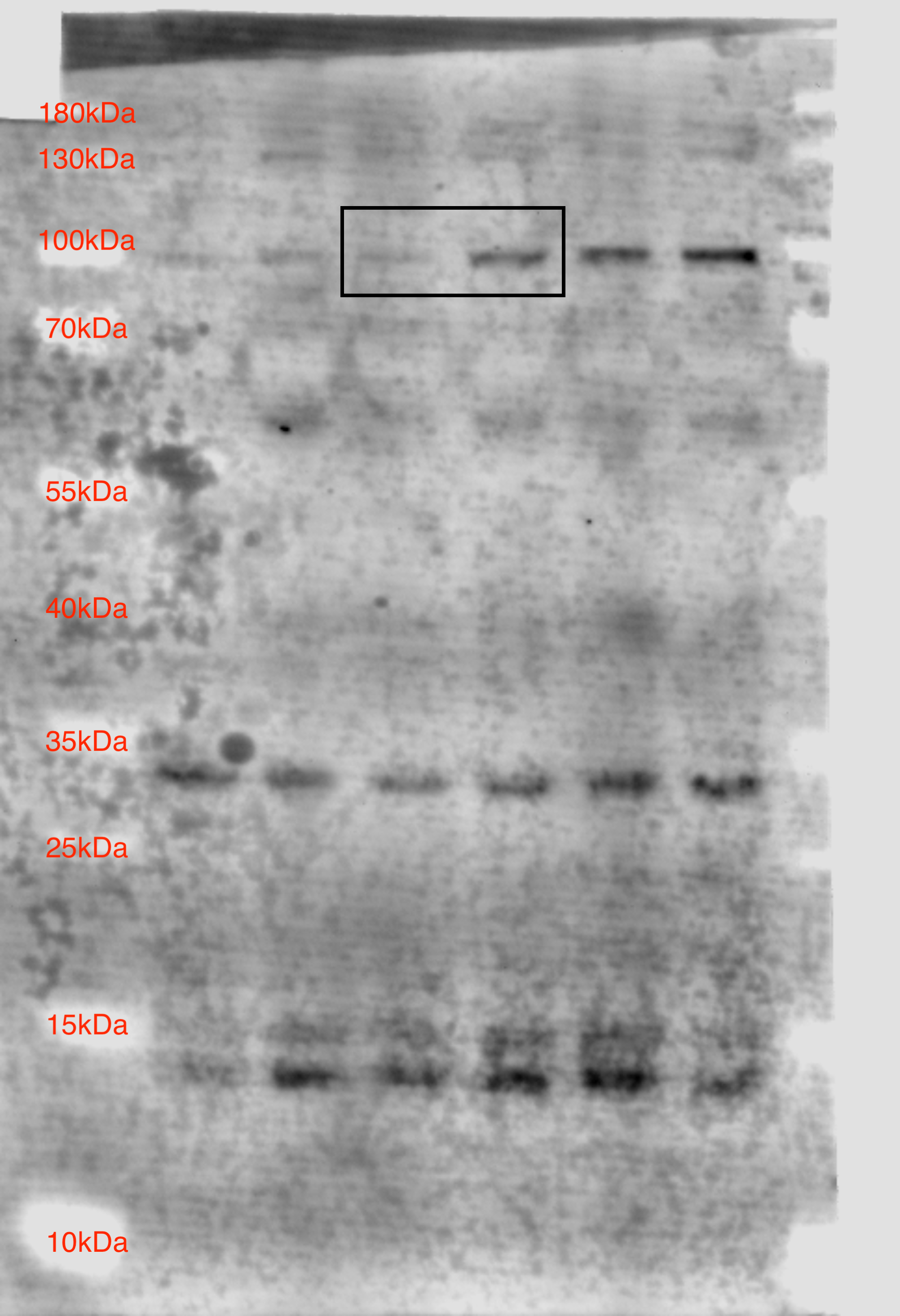

Supplement: Supplementary file 5 — Source Data for Figure 1 [file EMBJ-42-e112202-s008.zip › Figure 1/1E/p660 HSL BW.tif]

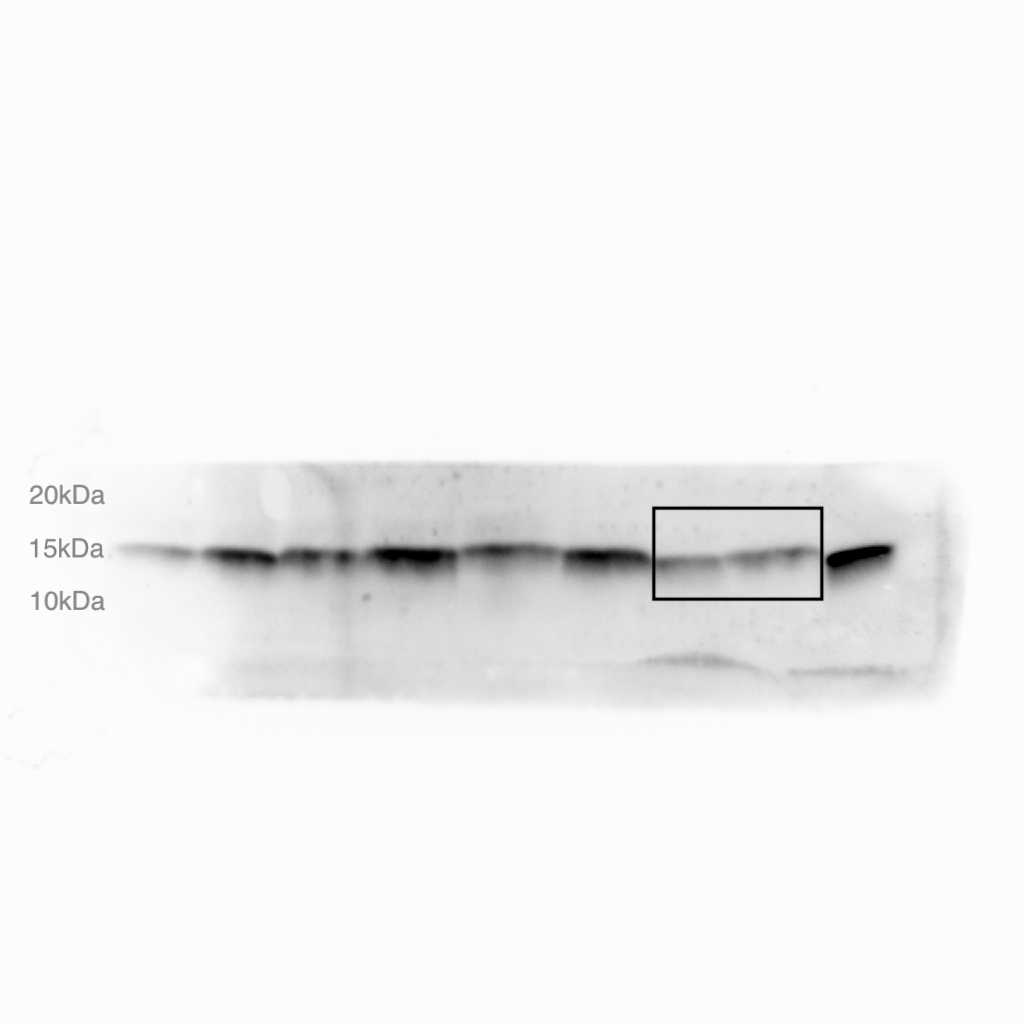

Supplement: Supplementary file 5 — Source Data for Figure 1 [file EMBJ-42-e112202-s008.zip › Figure 1/1J/LC3 - CC patients.tif]

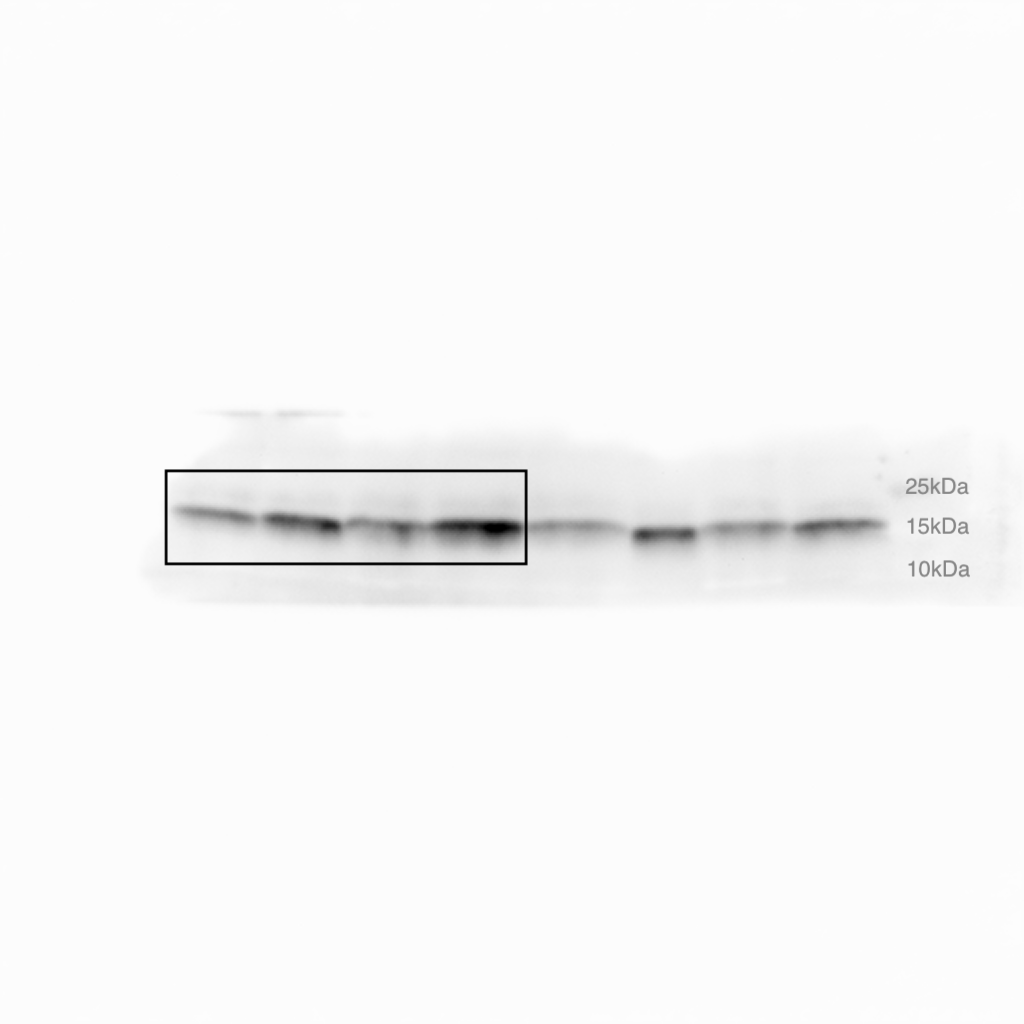

Supplement: Supplementary file 5 — Source Data for Figure 1 [file EMBJ-42-e112202-s008.zip › Figure 1/1J/LC3 - CD patients.tif]

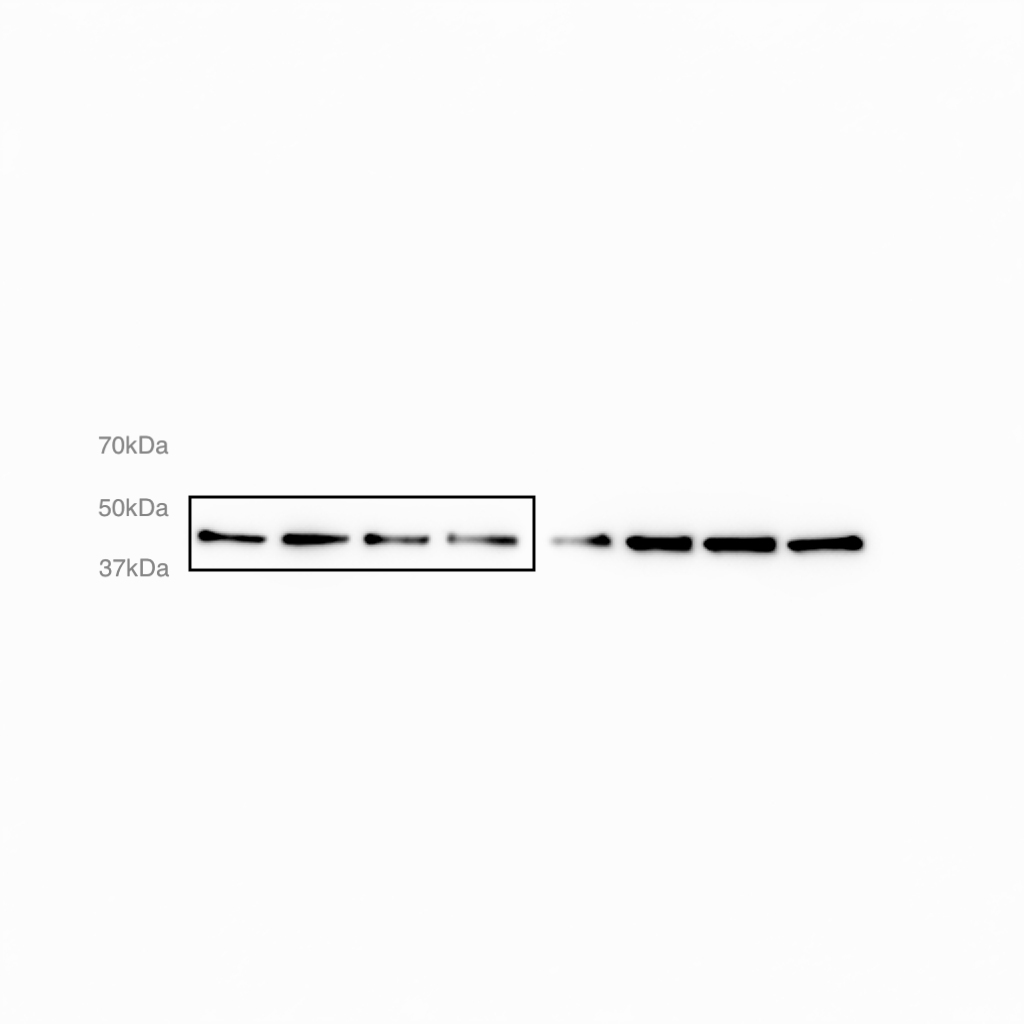

Supplement: Supplementary file 5 — Source Data for Figure 1 [file EMBJ-42-e112202-s008.zip › Figure 1/1J/ACTIN - CD patients.tif]

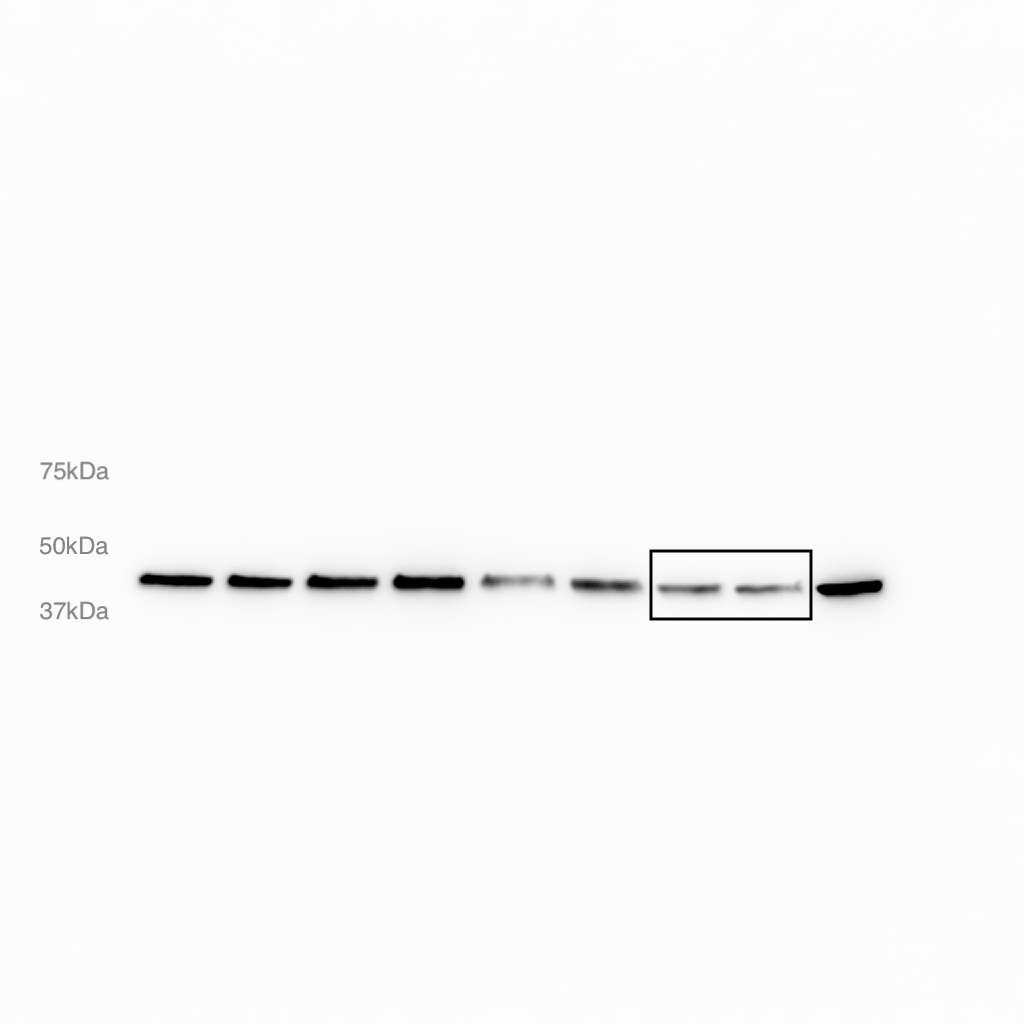

Supplement: Supplementary file 5 — Source Data for Figure 1 [file EMBJ-42-e112202-s008.zip › Figure 1/1J/ACTIN - CC patients.tif]

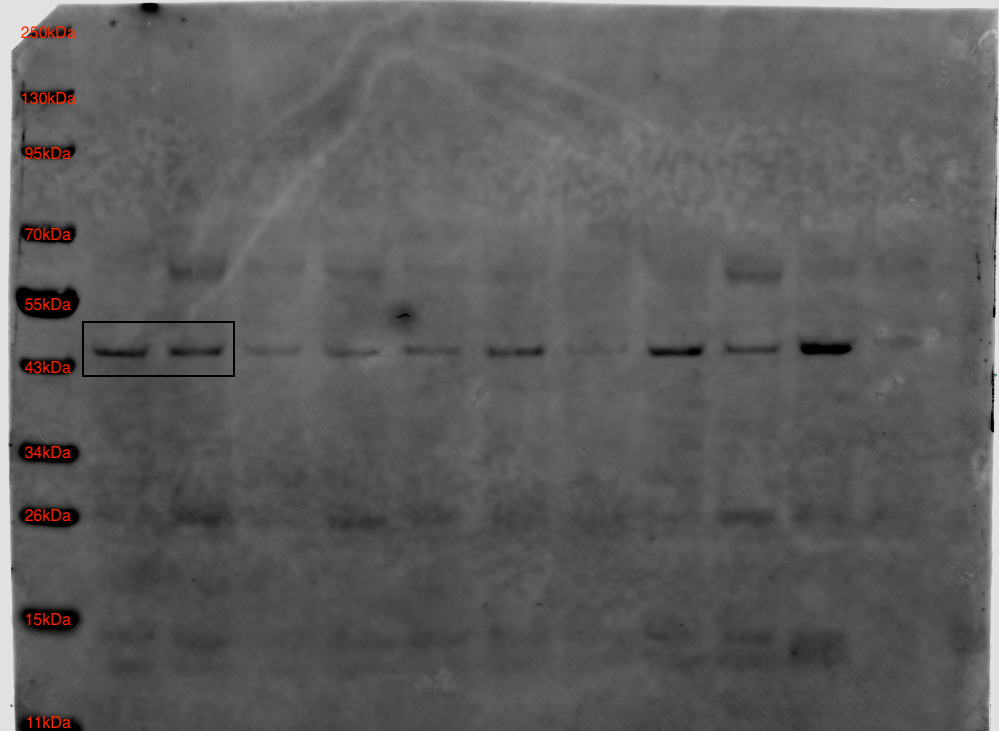

Supplement: Supplementary file 6 — Source Data for Figure 2 [file EMBJ-42-e112202-s010.zip › Figure 2/2C/iWAT 0mg TAM Actin.tif]

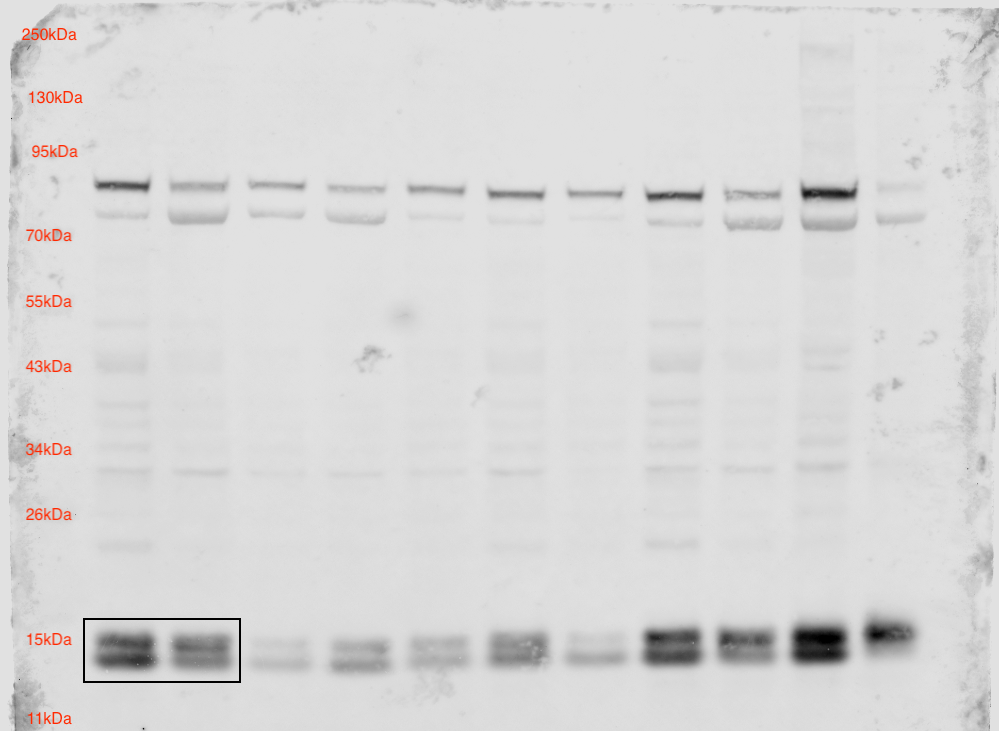

Supplement: Supplementary file 6 — Source Data for Figure 2 [file EMBJ-42-e112202-s010.zip › Figure 2/2C/iWAT 0mg TAM LC3.tif]

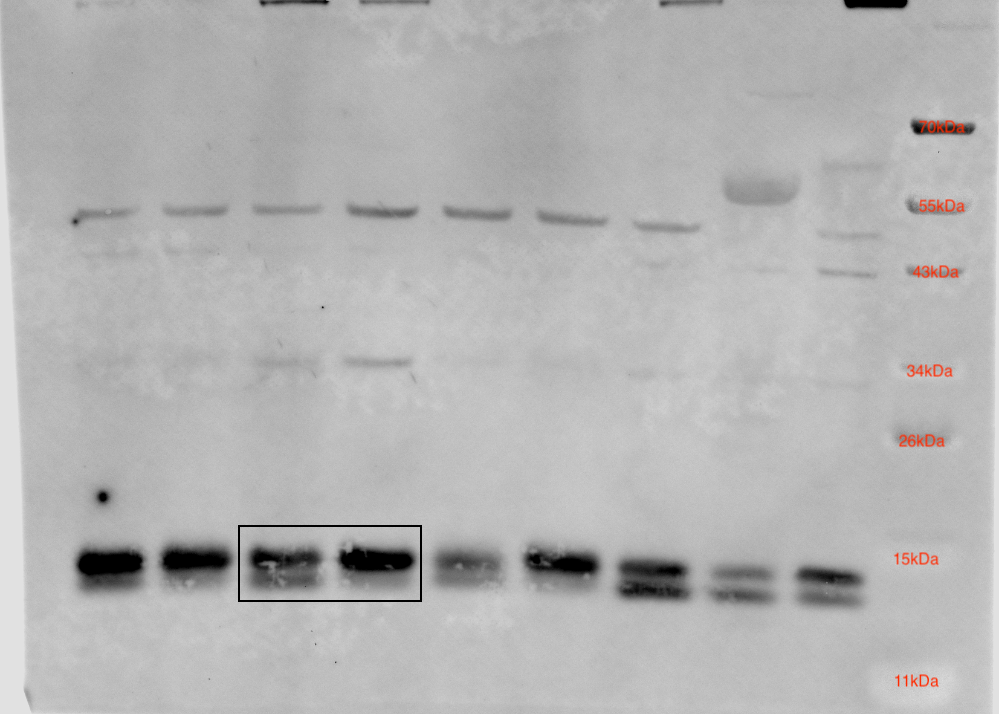

Supplement: Supplementary file 6 — Source Data for Figure 2 [file EMBJ-42-e112202-s010.zip › Figure 2/2C/iWAT 4mg TAM LC3.tif]

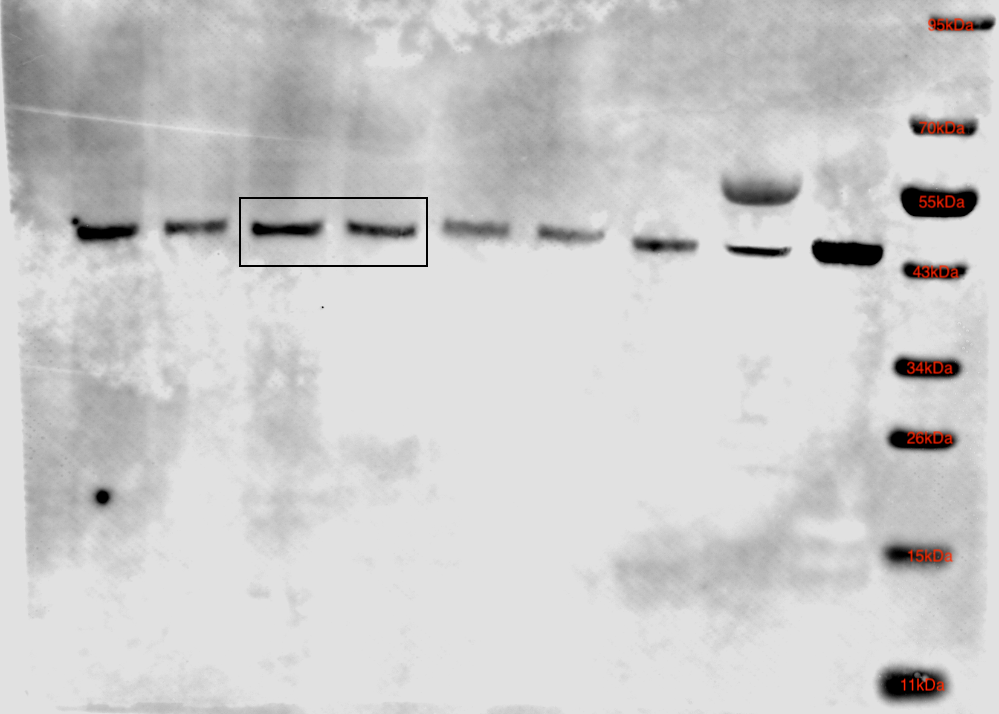

Supplement: Supplementary file 6 — Source Data for Figure 2 [file EMBJ-42-e112202-s010.zip › Figure 2/2C/iWAT 4mg TAM Actin.tif]

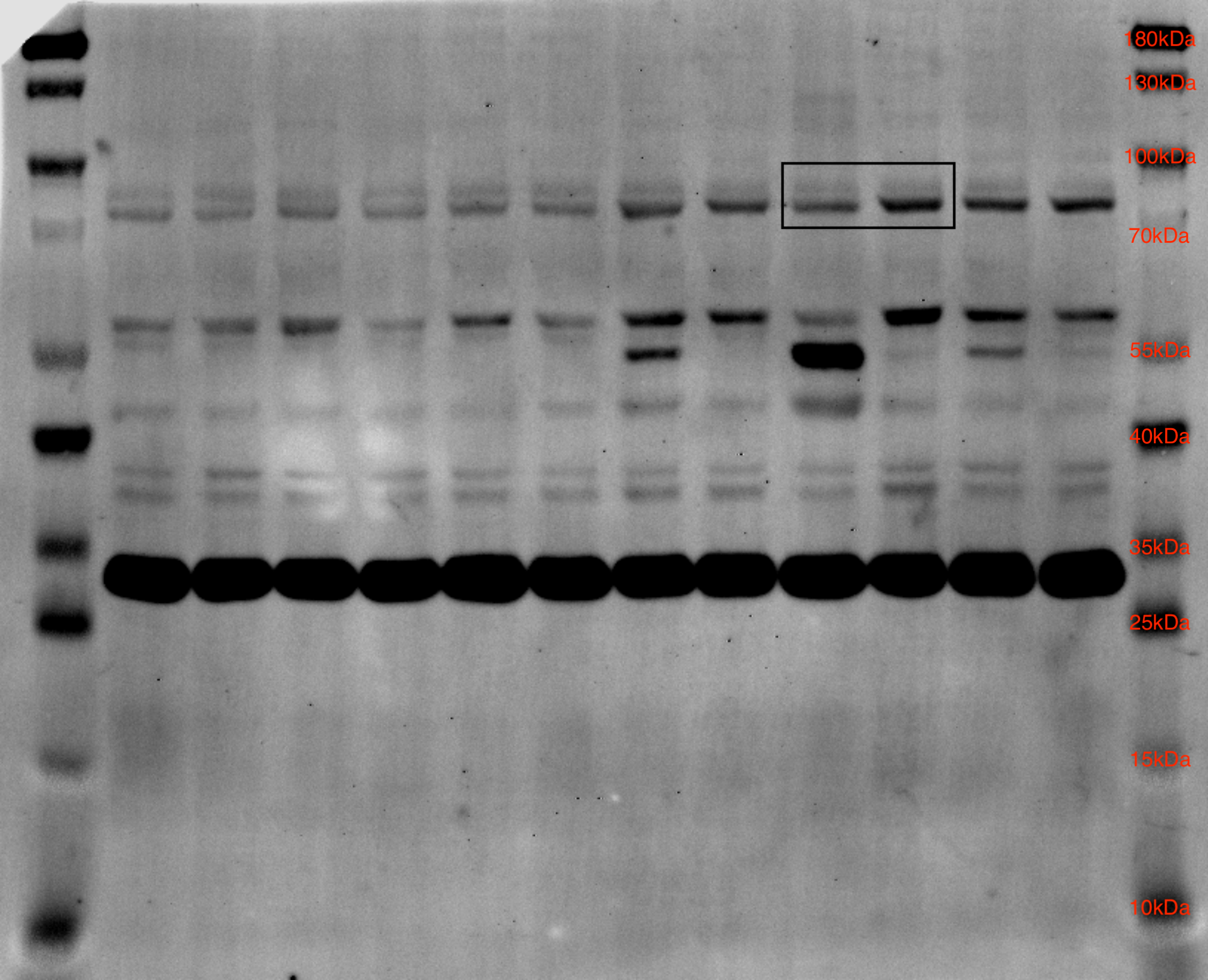

Supplement: Supplementary file 9 — Source Data for Figure 5 [file EMBJ-42-e112202-s006.zip › Figure 5/5F/gWAT NRF2.tif]

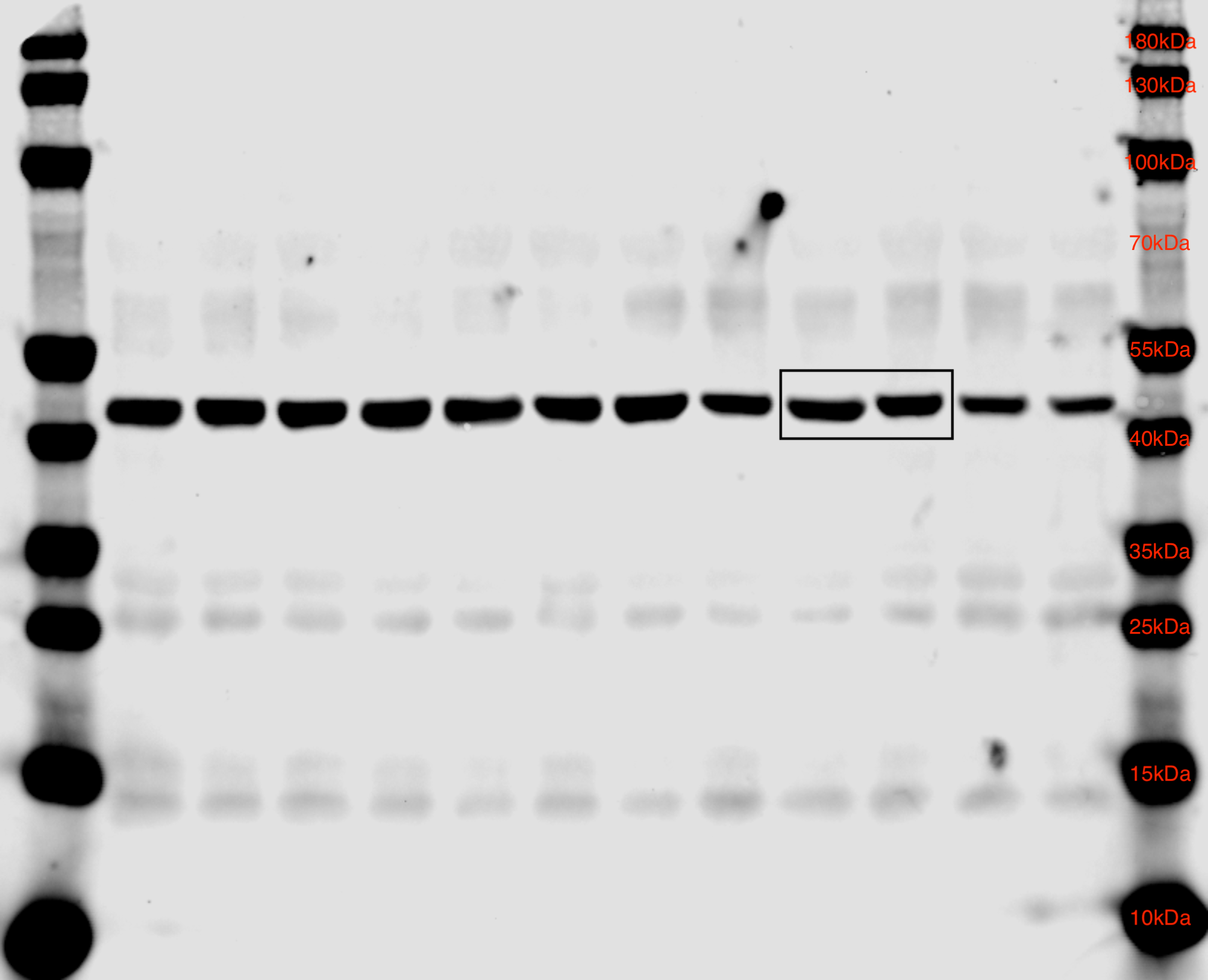

Supplement: Supplementary file 9 — Source Data for Figure 5 [file EMBJ-42-e112202-s006.zip › Figure 5/5F/gWAT Actin.tif]

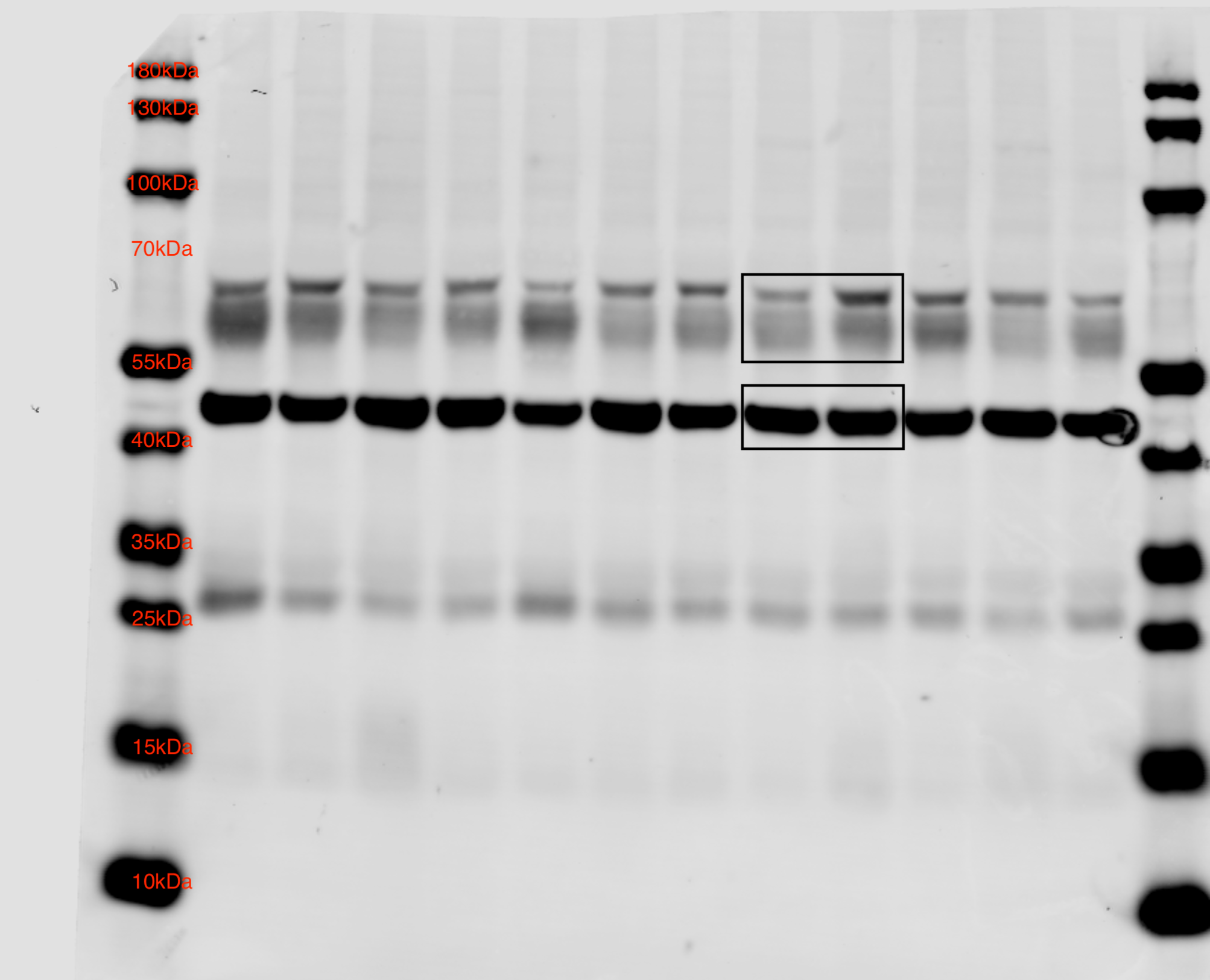

Supplement: Supplementary file 9 — Source Data for Figure 5 [file EMBJ-42-e112202-s006.zip › Figure 5/5H/gWAT EPHX2 ACTIN.tif]

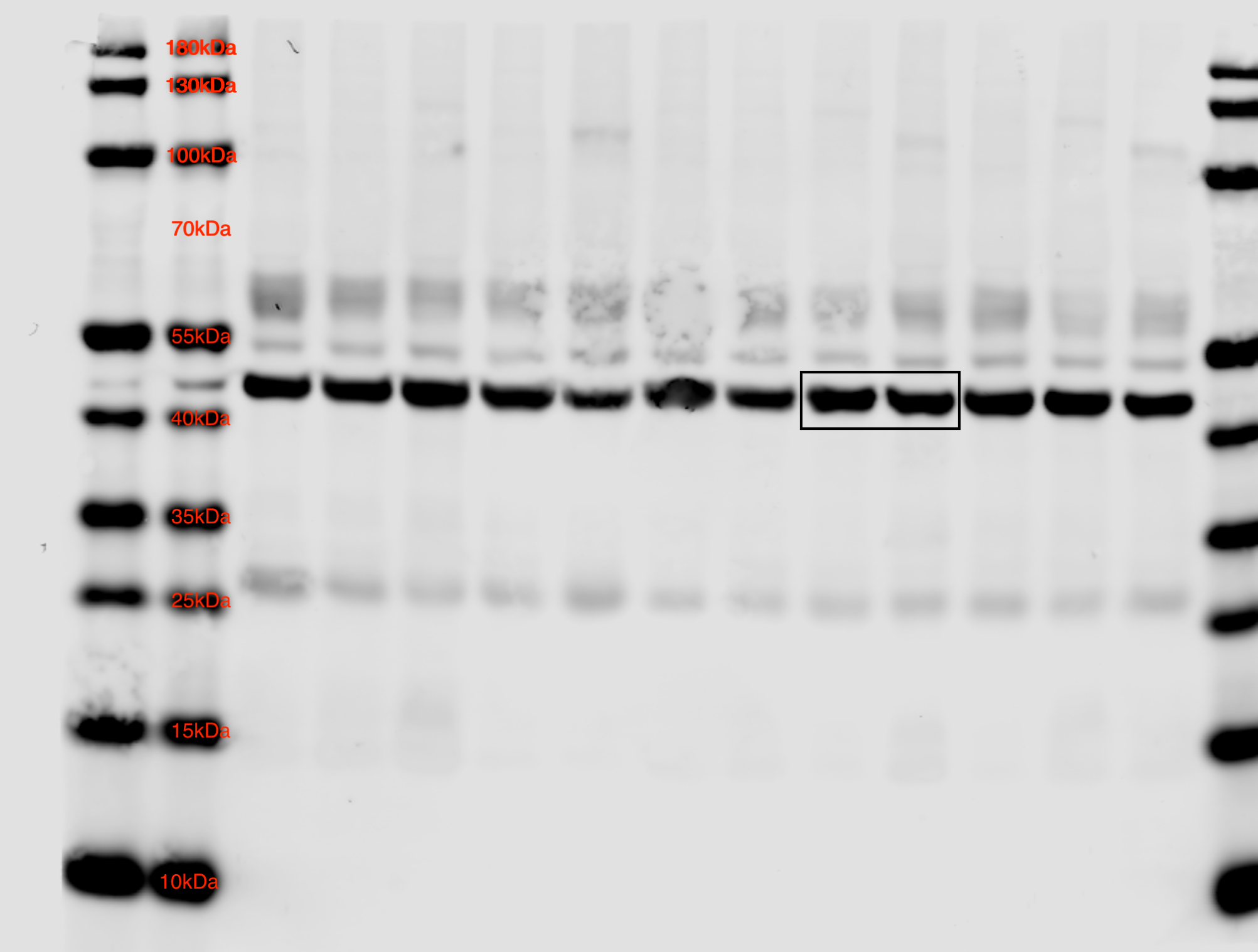

Supplement: Supplementary file 9 — Source Data for Figure 5 [file EMBJ-42-e112202-s006.zip › Figure 5/5H/gWAT ACTIN for EPHX1.tif]

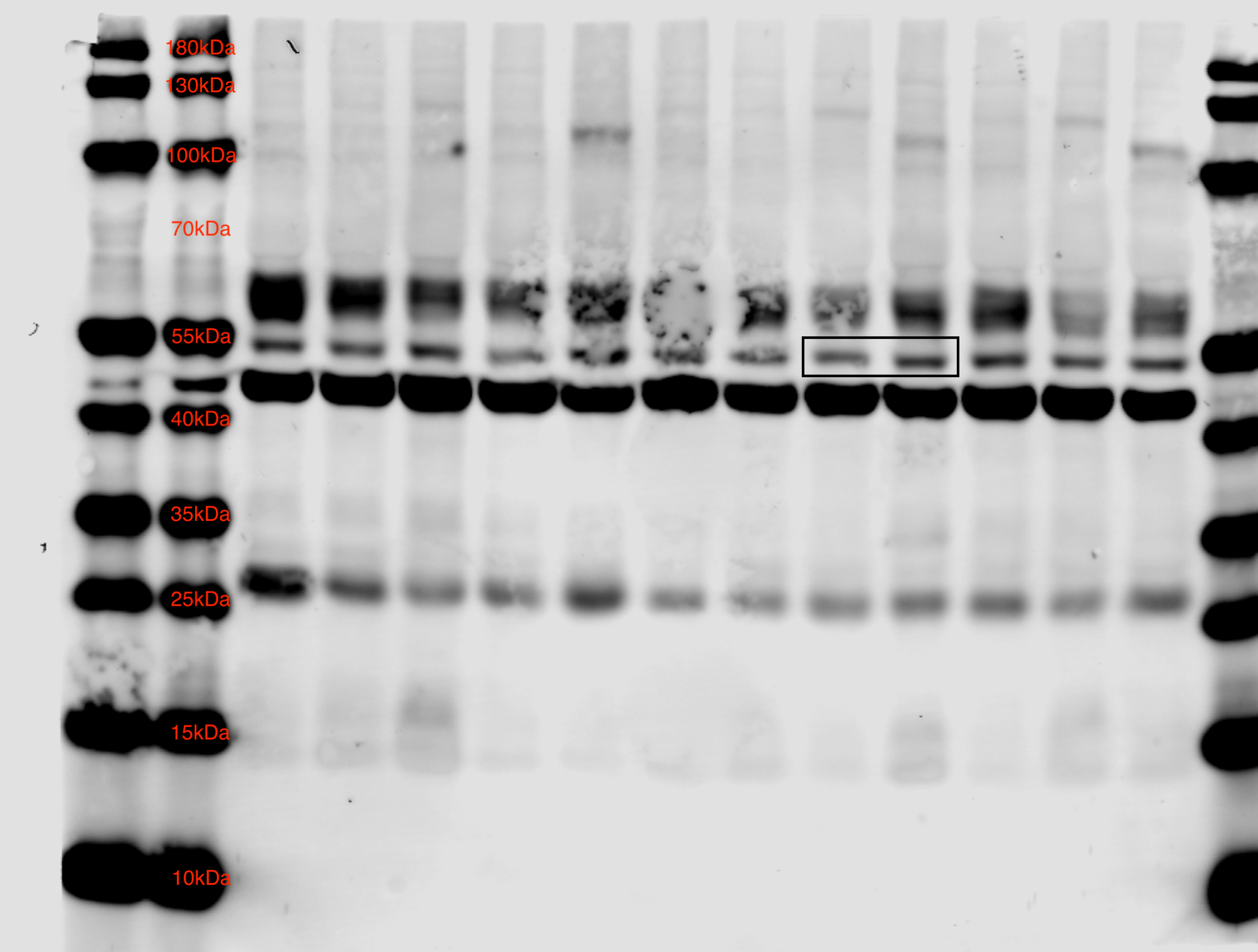

Supplement: Supplementary file 9 — Source Data for Figure 5 [file EMBJ-42-e112202-s006.zip › Figure 5/5H/gWAT EPHX1.tif]
