# Supplementary material for: Adipocyte autophagy limits gut inflammation by controlling oxylipin and IL‐10
Source: EMBO J. 2023 Feb 16;42(6):e112202. doi: 10.15252/embj.2022112202 (PMC10015370; doi:10.15252/embj.2022112202)
Supplement: Supplementary file 9 — Source Data for Figure 5 [file EMBJ-42-e112202-s006.zip › Figure 5/5F/README.rtf]

READMENRF2 antigen is supposed to run around 95-110 kDa. This band seems to run about 90/95 kDa and was therefore annotated as NRF2. https://www.ncbi.nlm.nih.gov/pmc/articles/PMC3503463/ 
