# Supplementary material for: Adipocyte autophagy limits gut inflammation by controlling oxylipin and IL‐10
Source: EMBO J. 2023 Feb 16;42(6):e112202. doi: 10.15252/embj.2022112202 (PMC10015370; doi:10.15252/embj.2022112202)
Supplement: Supplementary file 9 — Source Data for Figure 5 [file EMBJ-42-e112202-s006.zip › Figure 5/5H/README.rtf]

READMEEPHX1 and ACTIN were developed on the same membrane. Both proteins were targeted by a primary antibody raised in mouse, therefore we could not take advantage of the two channels of the LICOR machine. Band for EPHX1 was identified based on its described molecular weight: https://datasheets.scbt.com/sc-135984.pdf at about 52kDa.EPHX1 and ACTIN were developed on the same membrane. Both proteins were targeted by a primary antibody raised in mouse, therefore we could not take advantage of the two channels of the LICOR machine. Band for EPHX1 was identified based on its described molecular weight: https://datasheets.scbt.com/sc-166961.pdf at about 62kDa.
