# Supplementary material for: Adipocyte autophagy limits gut inflammation by controlling oxylipin and IL‐10
Source: EMBO J. 2023 Feb 16;42(6):e112202. doi: 10.15252/embj.2022112202 (PMC10015370; doi:10.15252/embj.2022112202)
Supplement: Supplementary file 12 [file EMBJ-42-e112202-s009.pdf]

THE EMBO JOURNAL

Adipocyte autophagy limits gut inflammation by controlling oxylipin-IL10

Felix Richter, Matthias Friedrich, Nadja Kampschulte, Klara Piletic, Ghada Alsaleh, Ramona Zummach, Julia Hecker, Mathilde Pohin, Nicholas Ilott, Irina Guschina, Sarah Wideman, Errin Johnson, Mariana Borsa, Paula Hahn, Christophe Morisseau, Bruce Hammock, Henk Schipper, Claire Edwards, Rudolf Zechner, Britta Siegmund, Carl Weidinger, Nils Schebb, Fiona Powrie, and Anna Katharina Simon
DOI: 10.15252/embj.2022112202

Corresponding author(s): Anna Katharina Simon (katja.simon@imm.ox.ac.uk)

Review Timeline:

Submission Date:	26th Jul 22
Editorial Decision:	24th Aug 22
Revision Received:	7th Dec 22
Editorial Decision:	11th Jan 23
Revision Received:	15th Jan 23
Accepted:	17th Jan 23

Editor: William Teale

Transaction Report:

(Note: With the exception of the correction of typographical or spelling errors that could be a source of ambiguity, letters and reports are not edited. Depending on transfer agreements, referee reports obtained elsewhere may or may not be included in this compilation. Referee reports are anonymous unless the Referee chooses to sign their reports.)

Dear Katja,

Thank you again for submitting your manuscript for consideration by the EMBO Journal. All three referees have now seen your responses to their reports and have commented on the plan to take the work forward that they contain. In this, not all referees were positive; reviewer 1 does not think that your plan addresses the concerns he/she raised.

That said, and given the other two referees' positive recommendations, I am comfortable with proceeding despite this opinion and would like to invite you to submit a revised version of the manuscript, addressing the comments of all three reviewers. I will ask reviewers 2 and 3 to consider the points raised by referee 1 in their re-reviews. I should add that it is EMBO Journal policy to allow only a single round of revision, and acceptance of your manuscript will therefore depend on the completeness of your responses in this revised version.

When preparing your letter of response to the referees' comments, please bear in mind that this will form part of the Review Process File, and will therefore be available online to the community. For more details on our Transparent Editorial Process, please visit our website: https://www.embopress.org/page/journal/14602075/authorguide#transparentprocess

We generally allow three months as standard revision time. As a matter of policy, competing manuscripts published during this period will not negatively impact on our assessment of the conceptual advance presented by your study. However, we request that you contact the editor as soon as possible upon publication of any related work, to discuss how to proceed. Should you foresee a problem in meeting this three-month deadline, please let us know in advance and we may be able to grant an extension.

Thank you for the opportunity to consider your work for publication. I look forward to your revision.

Best wishes,

William

William Teale, PhD
Editor
The EMBO Journal
w.teale@embojournal.org

When submitting your revised manuscript, please carefully review the instructions below and include the following items:

1) a .docx formatted version of the manuscript text (including legends for main figures, EV figures and tables). Please make sure that the changes are highlighted to be clearly visible.

2) individual production quality figure files as .eps, .tif, .jpg (one file per figure).

3) a .docx formatted letter INCLUDING the reviewers' reports and your detailed point-by-point response to their comments. As part of the EMBO Press transparent editorial process, the point-by-point response is part of the Review Process File (RPF), which will be published alongside your paper.

4) a complete author checklist, which you can download from our author guidelines (https://wol-prod-cdn.literatumonline.com/pb-assets/embo-site/Author Checklist%20-%20EMBO%20J-1561436015657.xlsx). Please insert information in the checklist that is also reflected in the manuscript. The completed author checklist will also be part of the RPF.

5) Please note that all corresponding authors are required to supply an ORCID ID for their name upon submission of a revised manuscript.

6) We require a 'Data Availability' section after the Materials and Methods. Before submitting your revision, primary datasets produced in this study need to be deposited in an appropriate public database, and the accession numbers and database listed under 'Data Availability'. Please remember to provide a reviewer password if the datasets are not yet public (see https://www.embopress.org/page/journal/14602075/authorguide#datadeposition). If no data deposition in external databases is needed for this paper, please then state in this section: This study includes no data deposited in external repositories. Note that the Data Availability Section is restricted to new primary data that are part of this study.

Note - All links should resolve to a page where the data can be accessed.

7) When assembling figures, please refer to our figure preparation guideline in order to ensure proper formatting and readability

in print as well as on screen:
http://bit.ly/EMBOPressFigurePreparationGuideline

Please remember: Digital image enhancement is acceptable practice, as long as it accurately represents the original data and conforms to community standards. If a figure has been subjected to significant electronic manipulation, this must be noted in the figure legend or in the 'Materials and Methods' section. The editors reserve the right to request original versions of figures and the original images that were used to assemble the figure.

8) For data quantification: please specify the name of the statistical test used to generate error bars and P values, the number (n) of independent experiments (specify technical or biological replicates) underlying each data point and the test used to calculate p-values in each figure legend. The figure legends should contain a basic description of n, P and the test applied. Graphs must include a description of the bars and the error bars (s.d., s.e.m.).

9) We would also encourage you to include the source data for figure panels that show essential data. Numerical data can be provided as individual .xls or .csv files (including a tab describing the data). For 'blots' or microscopy, uncropped images should be submitted (using a zip archive or a single pdf per main figure if multiple images need to be supplied for one panel). Additional information on source data and instruction on how to label the files are available at .

10) We replaced Supplementary Information with Expanded View (EV) Figures and Tables that are collapsible/expandable online (see examples in https://www.embopress.org/doi/10.15252/embj.201695874). A maximum of 5 EV Figures can be typeset. EV Figures should be cited as 'Figure EV1, Figure EV2" etc. in the text and their respective legends should be included in the main text after the legends of regular figures.

- For the figures that you do NOT wish to display as Expanded View figures, they should be bundled together with their legends in a single PDF file called *Appendix*, which should start with a short Table of Content. Appendix figures should be referred to in the main text as: "Appendix Figure S1, Appendix Figure S2" etc. See detailed instructions regarding expanded view here: .

- Additional Tables/Datasets should be labelled and referred to as Table EV1, Dataset EV1, etc. Legends have to be provided in a separate tab in case of .xls files. Alternatively, the legend can be supplied as a separate text file (README) and zipped together with the Table/Dataset file.

11) Our journal encourages inclusion of *data citations in the reference list* to directly cite datasets that were re-used and obtained from public databases. Data citations in the article text are distinct from normal bibliographical citations and should directly link to the database records from which the data can be accessed. In the main text, data citations are formatted as follows: "Data ref: Smith et al, 2001" or "Data ref: NCBI Sequence Read Archive PRJNA342805, 2017". In the Reference list, data citations must be labeled with "[DATASET]". A data reference must provide the database name, accession number/identifiers and a resolvable link to the landing page from which the data can be accessed at the end of the reference. Further instructions are available at .

Additional instructions for preparing your revised manuscript:

Please make sure you upload a letter of response to the referees' comments together with the revised manuscript.

Please also check that the title and abstract of the manuscript are brief, yet explicit, even to non-specialists.

When assembling figures, please refer to our figure preparation guideline in order to ensure proper formatting and readability in print as well as on screen:
https://bit.ly/EMBOPressFigurePreparationGuideline
See also guidelines for figure legends: https://www.embopress.org/page/journal/14602075/authorguide#figureformat

IMPORTANT: When you send the revision we will require
- a point-by-point response to the referees' comments, with a detailed description of the changes made (as a word file).
- a word file of the manuscript text.
- individual production quality figure files (one file per figure)
- a complete author checklist, which you can download from our author guidelines (https://www.embopress.org/page/journal/14602075/authorguide).
- Expanded View files (replacing Supplementary Information)
Please see out instructions to authors
https://www.embopress.org/page/journal/14602075/authorguide#expandedview

Please remember: Digital image enhancement is acceptable practice, as long as it accurately represents the original data and conforms to community standards. If a figure has been subjected to significant electronic manipulation, this must be noted in the

figure legend or in the 'Materials and Methods' section. The editors reserve the right to request original versions of figures and the original images that were used to assemble the figure.

Further information is available in our Guide For Authors: https://www.embopress.org/page/journal/14602075/authorguide

We realize that it is difficult to revise to a specific deadline. In the interest of protecting the conceptual advance provided by the work, we recommend a revision within 3 months (22nd Nov 2022). Please discuss the revision progress ahead of this time with the editor if you require more time to complete the revisions. Use the link below to submit your revision:

https://emboj.msubmit.net/cgi-bin/main.plex

Reviewer #1:

Richter and colleagues describe a pathway by which DSS-induced colitis induces autophagy in visceral adipose tissue, triggering an anti-inflammatory crosstalk with the gut, via IL-10. Inducible adipocyte-specific knockout of Atg-7 (Atg7ADKO) mice were generated to inhibit autophagy in the adipocyte and this exacerbated inflammation in the colon. While autophagy induced adipose tissue lipolysis, bulk FFA release was found to not alter inflammation in the colon. Instead, autophagy inhibition altered the balance of epoxy fatty acids (EpFA) and dihydroxl/diol fatty acids (DiolFA) production to a pro-inflammatory ratio. Since these oxylipins did not enter circulation, the authors propose that they trigger a local effect on adipose tissue macrophages increase the secretion of IL-10. The authors also determined that inhibition of adipocyte autophagy (during DSS-induced colitis) is associated with decreased circulating IL-10. However, it was not directly tested whether the small change in the ratio of the oxylipins regulate macrophage IL-10 secretion, or if the levels of IL-10 from adipose may be responsible for the crosstalk promoting gut inflammation. It is well established that gut inflammation is sensitive to the anti-inflammatory cytokine IL-10. Therefore, while most of the findings in this article are associations, the authors highlight a potential adipose/intestine crosstalk pathway that is clinically relevant and informative for future studies.
We appreciate that the reviewer highlights the novel adipose-gut crosstalk, however, this was not demonstrated by association alone. For example, we have clearly shown by knocking out key autophagy genes that autophagy in adipose tissues has anti-inflammatory properties. That this interaction may be mediated through the oxylipin-IL-10 pathway, we agree was by association, however, we have now added additional data that demonstrate direct regulation of IL-10 by oxylipins.

Major comments:

There are few issues with the key Fig. 2C that shows that Atg7 loss inhibits autophagy in adipocytes, and TAM itself does not induce autophagy.

1) It is not clear how to interpret the LC3II data since the actin loading control is invisible for one lane in Fig. 1E, 2C. It looks like the LC3II band is lower than the others, but it may be a loading issue not a biological effect.
We apologise for any uncertainty that the choice of our Western Blot may have caused and provide here below another more representative Western Blot clearly indicating lipidation of LC3B upon *Atg7* deletion from adipocytes (**NEW Figure 2C**). We have also repeated the Western Blots and find a consistent decrease in LC3-II/LC3-I ratios, which is the functional read-out for the lipidation of LC3 by ATG7.

NEW Figure 2C: Loss of Atg7 upon DSS induced colitis inhibits conversion of LC3-I into LC3-II. Representative immunoblot for LC3-I and LC3-II protein expression and quantification of LC3 conversion ratio (LC3-II/LC3-I) (n = 3/group).

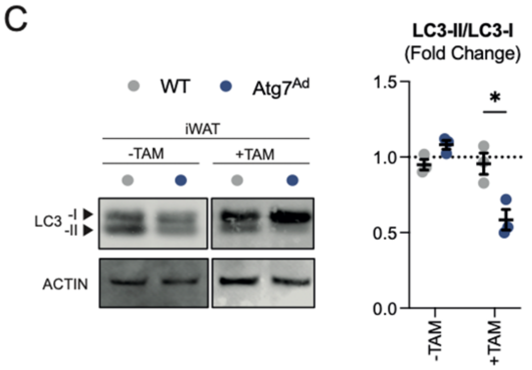

2) Tamoxifen treatment induces acute lipoatrophy and autophagy in adipose tissue within days.
The reviewer states and we were aware that tamoxifen has been reported to induce lipoatrophy and autophagy in adipose tissues. For this reason, we included a washout period of 2 weeks in our experimental design for all our experiments. This was determined from early pilot experiments, we found that mice of both genotypes were starting to gain weight again on day 5 after the last tamoxifen treatment (**Reviewer Figure 1**). With respect to adipose tissue

weights, Ye *et al.* (PMID: 26629402) previously showed that after 2 weeks lipoatrophy is largely recovered, and weight normalized to vehicle-treated controls. Additionally, we did not observe any changes in adipose tissue mass after tamoxifen treatment.

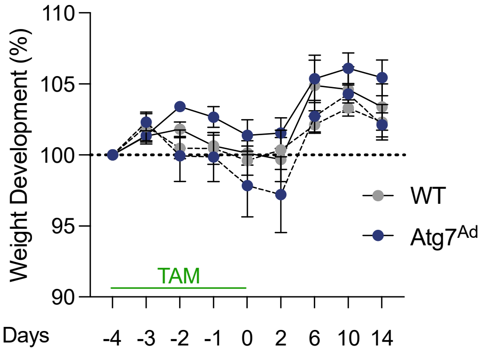

Reviewer Figure 1: Body weight development after tamoxifen administration over a 2-week washout period (before DSS). Dotted lines: Females, continuous lines: males.

Additionally, we had performed an experiment testing the effect of tamoxifen treatment on autophagic flux in inguinal and gonadal white adipose tissue (iWAT and gWAT, respectively) two weeks after tamoxifen treatment. We found that tamoxifen treatment indeed increases the autophagic flux in adipose tissue prior to any DSS treatment and thus tamoxifen treatment of both genotypes was warranted throughout the experimental setup. (**Reviewer Figure 2**). We have added this sentence to the manuscript in the result section:

> *"As tamoxifen is known to induce autophagy and we found this to be true in this setting, we included tamoxifen treatment for all genotypes and added a two-week wash-out period before treatment with DSS (Fig 2D)."*

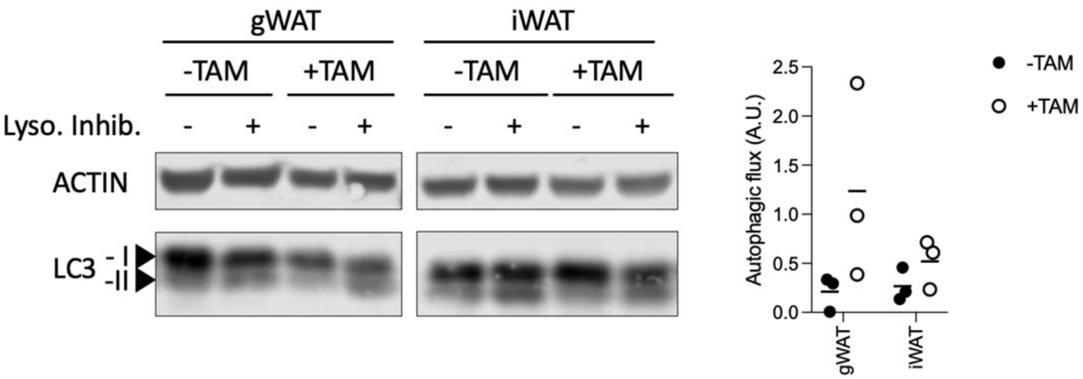

Reviewer Figure 2: Tamoxifen tends to increase autophagic flux in adipose tissues of wild-type mice. Mice were treated with tamoxifen and adipose tissues were collected 2 weeks after last tamoxifen administration and incubated either in the absence or presence of lysosomal inhibitors (100nM Bafilomycin A1 and 20mM NH_4Cl) for 4 hours. Proteins were blotted from inguinal (iWAT) and gonadal white adipose tissues (gWAT).

The only figure with a -TAM control group is Fig. 2C. The data is not interpretable since the variability was so high with only a n=3. Loss of Atg7 reduces LC3II in 2/3 samples vs. the -TAM control. Then the addition of TAM, further reduced LC3.

Therefore, when comparing the TAM control + DSS vs. TAM Atg7ADKO + DSS, the gut inflammation may exacerbate a tissue already sensitized for autophagy by TAM, not reflecting normal endogenous regulation. There are no problems with the studies with the Agtl KO mice or RNAseq that did not require TAM, but since the many of the Westerns in the paper involved TAM and had sample sizes higher than n=10 this figure should be revisited.

In addition to the **new Figure 2C**, **Figure 2B** clearly shows that prior to tamoxifen administration, transcriptional levels of *Atg7* are comparable to WT mice and that only after tamoxifen administration we find a profound reduction (>75%) in transcriptional *Atg7* levels.

We agree with the reviewer that tamoxifen may sensitise the adipose tissue and increase autophagy in the adipose tissue. However, *as the* underlying research question we aimed to answer was to investigate the role of autophagy in mature adipocytes, the model used is the most appropriate model with appropriate controls in place to study this question for the following reasons:

(1) The constitutive Cre recombinase system, (i.e. the non-inducible system without tamoxifen) would have confounded our study since it is well known that knocking out autophagy during differentiation inhibits adipose tissue development (Singh *et al.* (PMID: 19855132), Zhang *et al.* (PMID: 19910529)). Moreover, in an *in vitro* adipocyte differentiation assay we found that the onset of adiponectin expression (which is the driver of our Cre) correlates with high autophagic flux (**Reviewer Figure 3**). For both these reasons, we opted for the inducible system for our study.

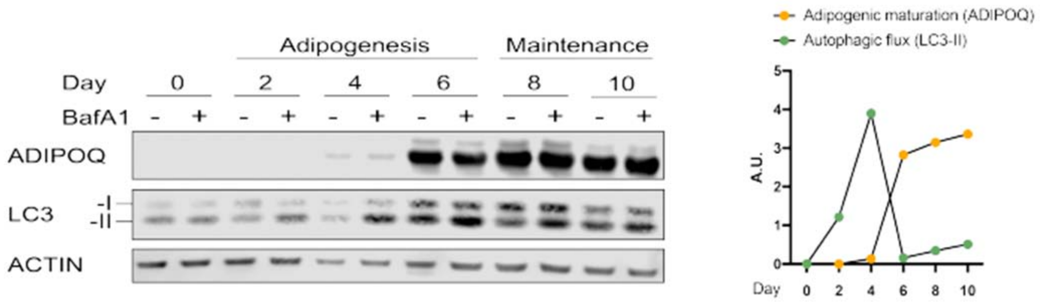

Reviewer Figure 3: In vitro adipogenesis of ST2 cells revealed an overlap of high autophagic activity with adiponectin expression. ST2 cells were differentiated over 6 days in vitro before adding maintenance medium for another 4 days. Every two days autophagic flux was inhibited through inhibition of Bafilomycin A1 (100µM) for 4h before harvesting. Adipocyte maturity was measured using adiponectin.

(2) In addition, we took extra precautions to limit effects of any tamoxifen-induced regulation of autophagy by including the two-week washout period in all our *in vivo* experiments.

(3) We also want to stress that we found that autophagic flux is consistently increased in visceral adipose tissue depots upon DSS-induced colitis even without tamoxifen administration in WT mice. This data has been confirmed on protein (**Fig 1F**) and transcript levels (**Fig 1H**) as well as by EM microscopy (**Fig 1G**). Thus, our data from **Figure 1** clearly demonstrate that autophagy in the adipose tissue is induced by intestinal inflammation in WT mice highlighting its potential relevance in this inflammation model.

Based on these findings and discussion, we added the following limitations to our discussion:

> *"Our study highlights that autophagic flux is increased in visceral adipose tissues of wild-type mice upon DSS-induced colitis. Since tamoxifen itself induces autophagy, it is possible that tamoxifen may potentiate some of the effects observed in the DSS-treated wild-type and $Atg7^{Ad}$ mouse model."*

3) Westerns for other markers of autophagy like p62 would be evidence that the pathway is activated.
We checked p62 protein levels, and while we found no difference in steady state it was increased in $Atg7^{Ad}$ adipose tissues under DSS conditions. See **Reviewer Figure 6A** below.

The authors do not report adipose tissue histology from Atg7ADKO mice, with or without DSS

administration. Do crown structures or adipocyte size worsen in Atg7ADKO mice, since it was shown that there as a reduction of M2-like macrophages?

We thank the reviewer for the suggestion. Crown-like structures (CLS) potentially indicate dying adipocytes and are observed when mice are put on high-fat diet and there they associate with M1-type macrophages. Furthermore, lipolysis can induce CLS formation and influx of macrophages into the adipose tissue (Kosteli *et al.* (PMID: 20877011), Tong *et al.* (PMID: 34504646)). While we did observe an increased presence of CLS upon DSS-induced colitis, both genotypes had the same number of CLS (**Reviewer Figure 4**). However, while CD206-expressing macrophages can also be part of CLS structures, their reduction as measured by flow cytometry (Fig EV6B) does not necessarily indicate a reduction in CLS formation (Springer *et al.* (PMID: 31323189)). We have now added this finding as a sentence to the description of Figure EV6:

> *"In adipose tissues, ATM frequencies were increased by DSS (Fig EV6A), which resulted in an increased presence of crown-like structures, however, this was comparable between wild-type and Atg7^{Ad} mice."*

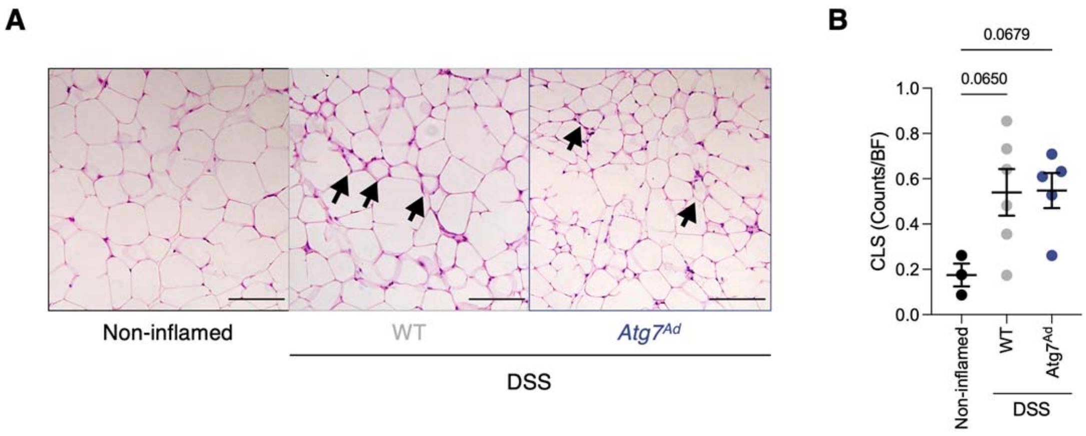

Reviewer Figure 4: CLS are increased at day 7 of DSS-induced colitis, independent of the genotype observed. Mesenteric adipose tissues from wild-type or *Atg7^{Ad}* mice treated with DSS-induced colitis were sacrificed at day 7 and fixed in formalin. Paraffin tissue sections were stained with H&E and CLS were counted in a blinded manner. (A) Representative histology sections for each group were presented. (B) Quantification of CLS per bright field section were shown.

The FFA assays representing changes in lipolysis would be more convincing if glycerol was measured.

Both read-outs, FFA and glycerol, are used interchangeably to measure lipolysis. As asked for by the reviewer, we repeated the experiments with glycerol as a readout and reproduced our earlier findings from the FFA assays, showing that autophagy-deficient adipocytes also release less glycerol upon stimulation with either isoproterenol (Iso) or TNFα (**NEW Figure 3A and 3B**, respectively).

The text was adjusted accordingly in the result section of the text:

> *"As expected, FFA and glycerol release was reduced upon lipolysis stimulation in autophagy-deficient as compared to autophagy-proficient adipocytes (**Fig 3A**)."*

> *"In the presence of TNFα, FFA and glycerol release was significantly blunted in autophagy-deficient compared to wild-type adipocytes (**Fig 3B**)."*

There is no data to show a link between adipose tissue macrophages IL-10 secretion and adipocyte oxylipins, as they did not treat macrophages with the lipids. Could conditioned

media from adipocytes +/- Atg7 used to treat macrophages? The proposition could be validated by including this type of experiment, because Fig. 6 describes the adipose autophagy oxylipin association which is totally independent from the Fig. 7 that shows the association between adipose autophagy and IL-10.

This is a great suggestion and has been raised by all 3 reviewers. We are demonstrating a direct link between oxylipin levels and IL-10 secretion in three different ways:

First, we investigated the effects of different epoxy fatty acids on Il-10 production from macrophages *in vitro*. To address this, we used RAW264.7 macrophages which were pre-treated with epoxy fatty acids and then stimulated with LPS. The addition of epoxy fatty acid 11,12-EET increased *Il10* transcriptional levels and both 11,12-EET and 19,20-EDP led to secretion of IL-10 into the supernatant after 12h of culture (**NEW Figure 7A and 7B,** respectively).

Second, we inhibited cytochrome P450 in adipose tissue explants from water- or DSS-treated WT mice by adding the pan-cytochrome P450 inhibitor 1-ABT *ex vivo* (see cartoon **Fig 5I**). 1-ABT prevents the CYP450-mediated production of epoxy fatty (Su *et al.* (PMID: 9688677)). The addition of 1-ABT significantly inhibited the DSS-induced IL-10 secretion from adipose tissue explants from WT mice (**NEW Figure 7C**), indicating a crucial role of cytochrome P450 in the secretion of IL-10 from the adipose tissue during DSS-induced colitis.

Third, we measured the effect of EPHX enzymes on the production of IL-10 by using EPHX inhibitors. For this, we extracted adipose tissue explants from DSS-treated WT or *Atg7^{Ad}* mice and cultured them either in the absence or presence of the EPHX1 inhibitor NTPA and EXPH2 inhibitor TPPU. This *ex vivo* inhibition resulted in a recovery of IL-10 secretion from *Atg7^{Ad}* adipose tissue explants (**NEW Figure 7D**), suggesting that EPHX enzymes activity is responsible for the regulation of IL-10 secretion from autophagy-deficient adipose tissues.

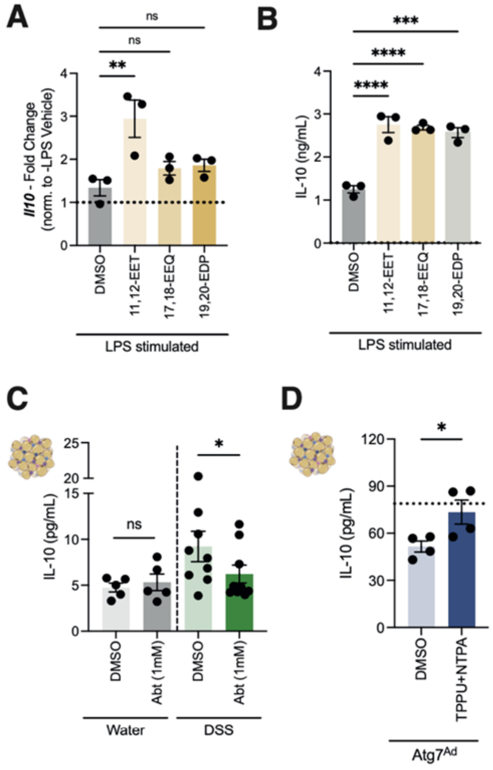

NEW Figure 7: Cytochrome P450-EPHX pathway regulates IL-10 secretion from autophagy-deficient adipose tissues upon DSS-induced intestinal inflammation.

(A) Quantification of *Il10* transcript levels in RAW264.7 upon stimulation with epoxy fatty acids (n = 3/group).

(B) Quantification of IL-10 protein levels in the supernatant of RAW264.7 upon stimulation with epoxy fatty acids (n = 3/group).

(C) Quantification of IL-10 protein levels in the supernatant of *ex vivo* cultured adipose tissues from water- or DSS-treated wild-type mice in the absence or presence of the cytochrome P450 inhibitor 1-ABT (n = 5-9/group).

(D) Quantification of IL-10 protein levels in the supernatant of *ex vivo* cultured adipose tissues from DSS-induced *Atg7^{Ad}* mice in the absence or presence of the EPHX1 inhibitor NTPA and EPHX2 inhibitor TPPU (n = 4/group).

Data are represented as mean ± s.e.m. (A,B) One-Way ANOVA. (C,D) Paired Student's t-test.

We included the following result section in the new manuscript:

"*Cytochrome P450-EPHX pathway regulates IL-10 secretion from autophagy-deficient adipocytes during intestinal inflammation*
*To establish a more mechanistic link between the increased function of the cytochrome P450-EPHX pathway and IL-10 in adipose tissues, we first determined whether EpFA supplementation improves IL-10 production from macrophages in vitro. Pre-treatment of RAW264.7 macrophages with different EpFA increased Il10 transcript levels upon LPS stimulation (**Fig 7A**), which was further confirmed on protein level in the supernatant (**Fig 7B**). Cytochrome P450 enzymes are key for the production of EpFA. Inhibition of cytochrome P450 resulted in a marked reduction of IL-10 secretion from DSS-induced wild-type adipose tissues (**Fig 7C**), suggesting that cytochrome P450 is crucial for adipose tissue-derived IL-10 during intestinal inflammation. Lastly, blockade of EPHX1 and EPHX2 in Atg7^{Ad} adipose tissue explants rescued IL-10 production (**Fig 7D**), establishing that EPHX enzyme activity can control IL-10 in autophagy-deficient adipose tissues. Collectively, this data indicates that EpFA and enzymes controlling their production and degradation can alter adipose tissue IL-10 levels during intestinal inflammation and this is dependent on autophagy."*

The downstream metabolites of AA and DHA were investigated, but were these precursor lipids changed by in adipocytes due to autophagy? Were other major signaling lipids derived from these precursors ruled out from the lipidomics data?
For this revision we addressed the abundance of different free, non-esterified fatty acids in each adipose tissue depot in the context of DSS-induced colitis. Relative to the WT, we found that adipose tissues from *Atg7^{Ad}* mice had lower levels of LA, while AA and DHA levels remained fairly stable. The decrease in LA may reflect the reduced lipolytic capacity of autophagy-deficient adipocytes, reducing the total levels of free, non-esterified LA in the tissue. This figure was added to the manuscript (**Reviewer Figure 5A, NEW Figure 5J**).

We previously performed a serum lipidomic profiling detecting more than 400 lipid species across 12 lipid classes (such as TAG, cholesterol, ceramides etc) of WT and *Atg7^{Ad}* mice treated with water or DSS-induced colitis. As expected, we found that DSS colitis shifted the serum lipid profile in the mice (**Reviewer Figure 5B**). However, we found no clear differential effects on any of the lipid classes between WT and *Atg7^{Ad}* mice (**Reviewer Figure 5C**). As such, we excluded effects of other major lipid classes in the observed "hyper-inflamed" phenotype.

To address the reviewer's comments further, we then focused on other signalling lipids associated with the arachidonic acid cascade outside the CYP450-EPXH pathway. We compared the expression of key enzymes in the LOX and COX pathways (**Reviewer Figure 5D**). Extracting the expression values from our RNAseq dataset, we found that most enzymes in the COX and LOX pathway were downregulated during DSS-induced colitis (**Reviewer Figure 5E**). In contrast, DSS-induced colitis increased the expression of oxylipin-producing cytochrome Cyp2j6 and j8 (by about 20-30%) in the adipocytes. Together, our transcriptomic data indicated significant changes only in the CYP450-EPXH pathway.

To investigate this pathway, we used targeted lipidomic approach measuring products of the 15-LOX pathway (15-HETE and 17-HDHA) and 5-LOX pathway (5-HETE, 4-HDHA and 7-HDHA). While we were unable to detect 15-HETE and 7-HDHA in the tissues, among the others, only the 5-LOX product 4-HDHA was significantly decreased in mWAT of *Atg7^{Ad}* mice, but not gWAT, during DSS-induced colitis (**Reviewer Figure 5F**). Together, with the transcriptional data, we conclude that LOX-derived products may play a small role, and their impact was not ultimately excluded.

This has been added to the discussion in the manuscript:

“Other oxylipin species, produced via the LOX and COX pathway, can also modulate DSS-induced colitis (Crittenden et al, 2021; Willenberg et al, 2015). However, most genes involved in these pathways were reduced in visceral adipocytes during DSS-induced colitis. It is plausible however that other oxylipins may contribute to the observed phenotype in the Atg7^{Ad} mouse model.”

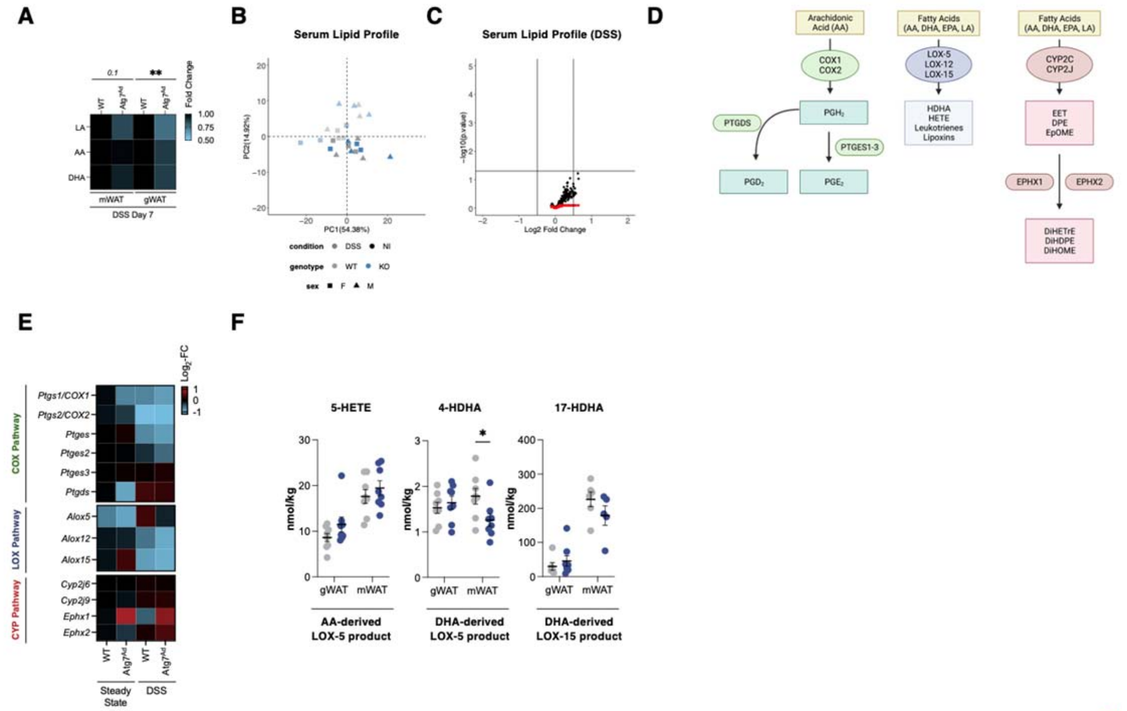

Figure Reviewer 5: Changes in lipid pathway genes and lipidomics in WT and Atg7^{Ad} during DSS induced colitis. (A) Changes in oxylipin precursor fatty acid species in mWAT and gWAT during DSS-induced colitis. (B) PCA analysis of serum lipidome of WT and *Atg7^{Ad}* mice during homeostasis and DSS-induced colitis. Light shading indicates DSS, colour indicates genotype and shape indicates sex of the mouse. (C) Volcano plot of serum lipid species present in the serum of DSS-treated WT and *Atg7^{Ad}* mice. (D) Schematic overview of COX, LOX and CYP450 pathway for the production of lipid mediators. (E) Expression of key enzymes in COX, LOX and CYP450 pathway in visceral adipocytes at steady state and during DSS-induced colitis. Data normalized to WT steady state mice. (F) Concentration of LOX pathway products in mWAT and gWAT in DSS-treated WT and *Atg7^{Ad}* mice.

The data clearly shows that in response to DSS administration, the Ephex1 increases, and Atg7 inhibits this. However, the proposed mechanism that regulates Ephex1 is questionable. The authors suggest that this is mediated by Nrf2, but the data for Nrf2 target gene expression all have a very low fold change, and the gene expression data was the basis of the rationale. Nrf2 protein was increased, but any other marker for Nrf2 activation was not presented. Showing adipocyte p62 mRNA and protein levels, levels of other associated proteins that regulate Nrf2 like Keap1, and canonical Nrf2 target gene expression would support whether Nrf2 is active.

We thank the reviewer for the comment. Previous reports indicate that loss of *Atg7* can increase Nrf2 activation (Cai *et al.* (PMID: 30428342)). In line with the loss of autophagy, we found that p62 does indeed accumulate in gonadal adipose tissue of *Atg7^{Ad}* mice in the DSS condition. (**Reviewer Figure 6A**). Interestingly, we were unable to observe an increased accumulation of KEAP1 (**Reviewer Figure 6B**). This may be due to the fact that we used the detergent soluble protein fraction since we used Triton-X to reduce lipid burden during protein

extraction (Komatsu et al (PMID: 20173742) see Fig 4). This confirms the findings by Cai *et al.* (PMID: 30428342) who also found a limited accumulation of KEAP1 in autophagy-deficient adipocytes. In line with our data, the authors found an increase in NRF2 protein levels and increase of NRF2-target genes.

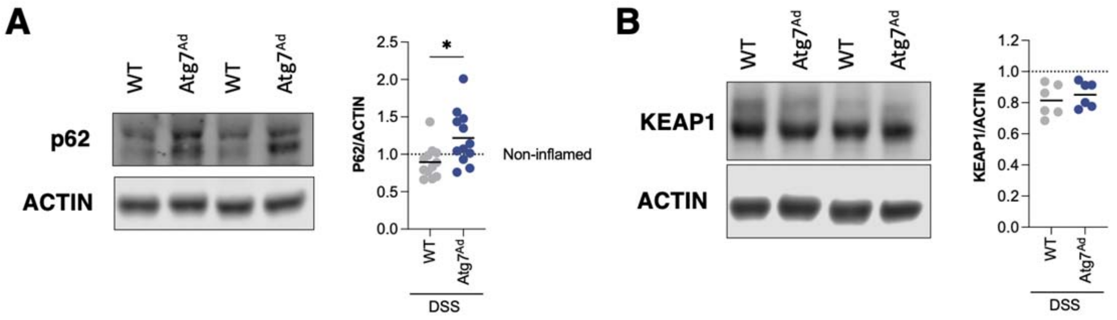

Reviewer Figure 6: Adipocyte autophagy loss results in increased p62 protein accumulation. (A) Immunoblotting for p62 in WT and *Atg7*^{Ad} mice upon DSS-induced colitis showing an accumulation of p62 in autophagy-deficient adipocytes, measured in gonadal adipose tissue. Data pooled from two independent experiments. (B) Immunoblotting for KEAP1 showed no accumulation of KEAP1. Data representative for two independent experiments.

One of the differentially expressed genes from Fig. 6C is Esr1. Estrogen controls macroautophagy signaling and mitophagic flux in white adipocytes and Nrf2 activity. Maybe that is what is post-transcriptionally regulating Nrf2? Or Era regulating autophagy independently? Since this could be a good candidate is there a reason this was not addressed? https://www.ncbi.nlm.nih.gov/pmc/articles/PMC8212422/

Initially, we were also quite surprised to see this strong Esr1 expression. However, we realized that this was probably due to our experimental setup, where we used WT mice (*Atg7*$^{f/f}$) and their *Atg7*^{Ad} (*Adipoq-Cre*^{ERT2} *Atg7*$^{f/f}$) littermates. The ERT2 is a human-derived, mutated estrogen receptor 1, with increased affinity to tamoxifen (Indra et al. (PMID: 10536138)). In contrast to the reviewer's suggestion, we hypothesize that the high expression of *Esr1* is due to the human transgene expressed by our KO mice, which carry the *Cre*^{ERT2} allele. Indeed, the sequence homology of the ERT2 transgene to human *ESR1* is 99%, and its homology to the mouse *Esr1* is about 86%. To assess whether the Kallisto alignment accidentally picked up the human *ESR1* in the RNAseq and mapped it to the mouse *Esr1* transcript, we firstly used mouse-specific Taqman qPCR specific probes to mouse *Esr1* and found mouse *Esr1* transcripts were not upregulated in *Adipoq-Cre*^{ERT2} *Atg7*$^{f/f}$ visceral adipocytes (**Reviewer Figure 7A**). We furthermore re-analyzed our transcriptomic sequence alignment with the pseudoalignment tool Kallisto and included the human *ESR1* sequence in our reference genome. Re-alignment shows clearly that our KO *Atg7*^{Ad} mice had an artificial increase in mouse *Esr1* transcripts when the alignment was performed only using the mouse *Esr1*, but not when human *ESR1* transcript were included (**Reviewer Figure 7B**). Finally, the normalized counts of the mouse Esr1 transcript were comparable across the groups and treatment conditions (**Reviewer Figure 7C**).

We added a disclaimer to the high Esr1 expression levels in the result section:

> *"The latter was verified to be caused by the overexpression of the Cre-ERT2 construct which mapped to mouse Esr1."*

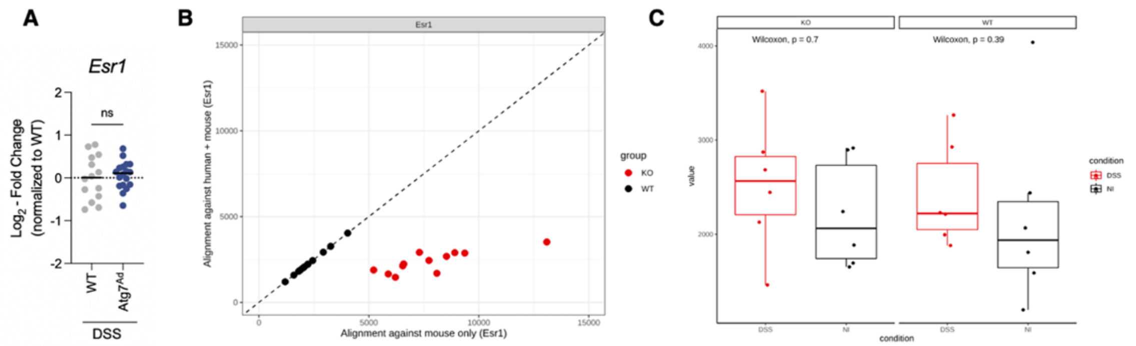

Reviewer Figure 7: Atg7 loss in adipocytes does not increase mouse Esr1 transcripts. (A) Using RNA extracted from visceral adipocytes, we quantified *Esr1* transcript levels using mouse specific Taqman probes (Mm00433149_m1), showing no difference in endogenous *Esr1* expression in mice. (B) Correlation between mouse Esr1 counts, when Kallisto reference genome contained the human ESR1 transcript (y-axis) or only the mouse genome (x-axis). The data indicates that KO mice have an artificially high level if only aligned to the mouse genome (x-axis) but is not different when compared to a reference genome that contained both the mouse *Esr1* and human *ESR1* sequence (y-axis). (C) Comparison of counts of the mouse *Esr1* transcript across experimental groups.

Minor comments:

Understandably use of DSS results in high variability, and some experiments required many mice to see a small effect in the treatment group. However, several panels have data points that look like outliers that may be skewing the data towards the desired outcome. How were outliers removed?
Aside from our previous data processing during which we excluded only samples with clear technical errors (e.g. errors during sample preparation or similar), we now also included a ROUT analysis that identifies any data outliers and removed them from further analysis. We tested all datasets for normal distribution before applying any appropriate testing.

Could it be clarified why 19, 20 EpDPE was highlighted rather any of the other significantly upregulated oxylipins for Fig. 6J?
We highlighted 19,20-EpDPE since it has been previously linked to anti-inflammatory functions and alterations of IL-10 levels (McDougle et al. (PMID: 28687674)). In the revised version of this manuscript, we have removed the figure from the paper and show instead the changes in ratios for all EpFA:DiolFA pairs (**NEW Fig EV4D**).

Fig. 3F shows DSS administration increases adipocyte pHSL. It is suggested that lipolysis is responsible for the decrease in FFA in Atg7ADKO mice (Fig. 4). It is not shown whether adipocyte pHSL changes in the Atg7ADKO mice.
We thank the reviewer for this interesting question. To address this, we harvested gWAT on day 7 after DSS administration. As previously observed, we find that HSL was more phosphorylated at serine residue 660 (in line with activated lipolysis) compared to non-inflamed control mice in WT mice. This increase, however, is no longer observed in *Atg7^{Ad}* mice (**NEW Figure 3C**). These findings are in line with our findings that *Atg7^{Ad}* mice were less lipolytic compared to wild-type mice and suggests an intriguing crosstalk between the two pathways.

C

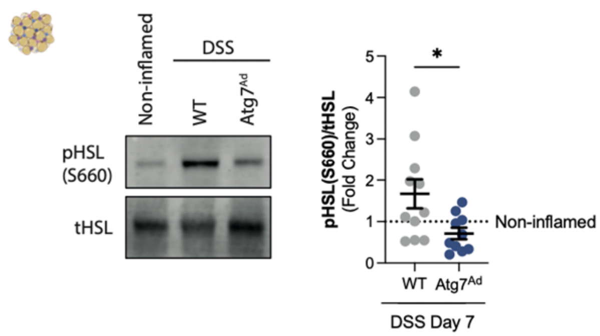

NEW Figure 3C: Adipocyte-specific loss of Atg7 (Atg7^{Ad}) results in reduced phosphorylation of HSL during DSS-induced colitis. (C) Representative Western Blot for phosphorylation of serine-660 and total HSL levels (left). Quantification of pHSL levels upon DSS induced colitis. Dotted line corresponds to non-inflamed control (right). Values were normalized to non-inflamed control. Data representative for two independent experiments.

The corresponding lines were added in the result section:

> *"Induction of HSL phosphorylation of HSL was reduced in adipose tissues of Atg7^{Ad} mice, suggesting a reduced lipolytic potential of autophagy-deficient adipocytes (**Fig 3C**)."*

And this sentence was added to the discussion:

> *"In addition, we found that phosphorylation of HSL was reduced in autophagy-deficient adipocytes, which could indicate a role of autophagy in regulating upstream kinase activity, such as PKA, during intestinal inflammation."*

Text is very small and blurry in Fig. 3C.
We corrected this. It was most likely a consequence of compression of the original file.

There are no error bars on any of the figures.
We thank the reviewer for pointing this out and have reformatted the data according to the journals' statistics reporting guidelines using the mean +/- sem.

Altogether, there is limited enthusiasm for the data presented.
We hope that, with the newly added data, we could elicit more enthusiasm for the presented study, its conceptual insights into the crosstalk between adipose tissues and the intestine and control of signalling lipids by macroautophagy.

Reviewer #2:

The genetic association of autophagy and IBD has launched studies that have defined anti-inflammatory roles of the pathway in distinct cellular compartments, especially the intestinal epithelium and myeloid cells. In this manuscript, the authors examine how autophagy in adipose tissues limits intestinal inflammation. Richter et al find that DSS-induced intestinal inflammation increases autophagy in adipose tissue and that the loss of autophagy through deletion of ATG7 in adipocytes exacerbates inflammation. Rather than through a mechanism involving the availability of free fatty acids, they show that this loss of autophagy results in a shift in oxylipin levels downstream of NRF2-mediated transcription of Ephx1, which results in decreased secretion of IL-10 from adipose tissue macrophages. The authors propose that this loss of anti-inflammatory IL-10 exacerbates the intestinal inflammation observed in DSS-treated mice that are deficient in autophagy in adipose tissue.

This study sheds important light on the understudied interaction between adipose tissue and the gut. The experiments are generally carried out well. Also, the proposed mechanism is quite interesting and should appeal to the journal's broad readership.
We thank the reviewer for their interest in the study and the results.

One weakness is the effect of deleting Atg7 on inflammatory outcomes (weight loss and histology) is modest. This may be difficult to address.
We believe the effect sizes observed are small because the adipose tissue is one of the many important components that fine tunes the inflammatory response but does not cause or prevent intestinal inflammation in its own right.

Another weakness is the evidence supporting the mechanism downstream of Atg7 deletion is mainly correlative.
We have now provided further evidence that dysregulation of the oxylipin pathway changes IL-10 production in the adipose tissue and can alter macrophage IL-10 expression levels. We believe this newly added data further supports the proposed mechanism.

Specific points:

1. It would be helpful if the authors can discuss why they believe autophagy is induced in adipose tissue upon DSS treatment. This was not clearly explained in the text. Perhaps the analysis in Fig 3 can help address this question.
During DSS induced colitis, the gut barrier becomes disrupted and leaky. The disruption of the epithelial barrier will activate the intestinal immune system leading to an increased production of TNFα (Noti et al. (PMID: 20439544)) (**EV Fig 1D**). Previous reports show that autophagy can be induced in 3T3-L1 differentiated adipocytes using TNFα (Ju et al. (PMID: 30741926)). We therefore hypothesized that TNFα may regulate adipocyte autophagy during DSS-induced inflammation. We have now gathered data suggesting that autophagic flux in the adipose tissue is controlled by TNFα as antibody-mediated blockade of TNFα *in vivo* reduces autophagy levels in the adipose tissue (**NEW Fig 1I**).

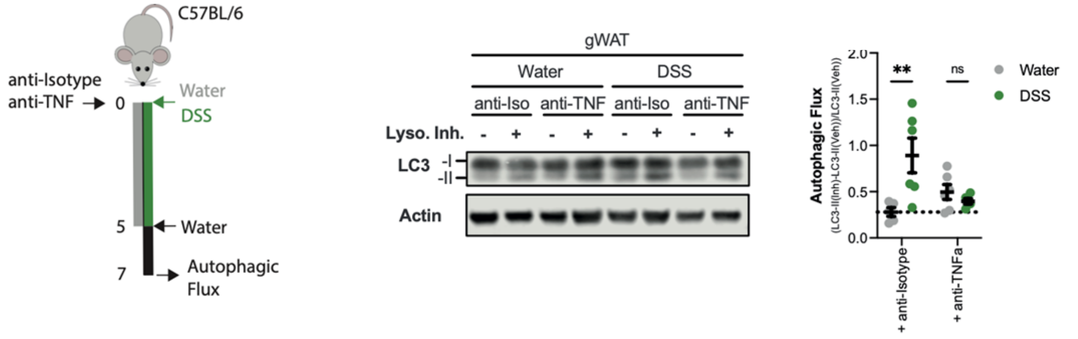

NEW Figure 1I-J: Antibody-mediated blockade of TNFa reduced autophagic flux in gonadal adipose tissues. (A) Mice were treated with either TNF*a*-neutralizing antibody or isotype control at the start of the DSS regime. (B) Subsequently, gonadal white adipose tissues were isolated and cultured in the absence or presence of lysosomal inhibitors (100nM Bafilomycin A1 and 20mM NH_4Cl). (C) Quantification of immunoblots. Data collected from three independent experiments.

We also ensured that mice on anti-TNF treatment would still become inflamed as it was apparent by the reduced colon length and the decrease in BW (**NEW Fig EV1G-H**). This was in line with previous reports showing that loss of TNF did not prevent intestinal inflammation. Interestingly, we also found that anti-TNF treatment partially rescued adipose tissue loss (**NEW Fig EV1I**). This can be explained by the fact that TNF is also a potent inducer of lipolysis, and that blocking TNF may reduce adipose tissue lipolysis.

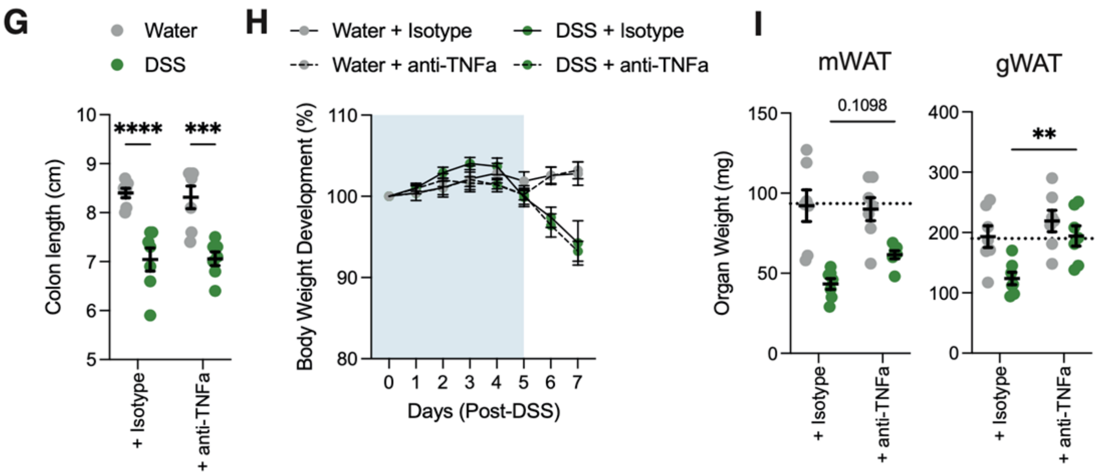

NEW Figure EV1G-I: Antibody-mediated blockade of TNFa does not change induction of intestinal inflammation upon DSS colitis but may partially preserve adipose tissue mass. (G) Colon length measurement upon treatment with TNF*a*-neutralizing antibody or isotype control at the start of the DSS regime. (H) Body weight development upon treatment with TNF*a*-neutralizing antibody or isotype control at the start of the DSS regime. (I) Adipose tissue weights for mWAT and gWAT upon treatment with TNF*a*-neutralizing antibody or isotype control at the start of the DSS regime.

We added this data into the manuscript with the corresponding section in the result section:

> *"TNFα has previously been shown to be a potent inducer of autophagy in in vitro differentiated 3T3-L1 cells, prompting the hypothesis that the release of TNF during DSS-induced intestinal inflammation augments autophagic flux in the adipose tissue. To test this, we blocked TNFα in vivo using a neutralizing antibody (Fig 1I). Mice treated with anti-TNFα and anti-Isotype showed similar body weight loss and colon shortening during DSS-induced inflammation indicating that mice were similarly inflamed (Fig EV1G-H). In contrast, loss of adipose tissue mass was partially prevented by neutralization of TNFα (Fig EV1I), possibly indicating a reduced release of lipids from the adipose tissue. Importantly, we found that adipose tissue autophagic flux was reduced upon anti-TNFα treatment (Fig 1J)."*

2. It is reasonable to assume that altered IL-10 levels contribute to the exacerbated inflammation because of the established role of this cytokine. However, there is a lack of direct evidence showing that NRF2 and oxylipin mediates inflammation in DSS-treated Atg7 AD mice. There are genetic tools available to investigate NRF2/KEAP1 in mice, although it may be difficult to generate appropriate animal lines during the revision period. Would it be possible to exogenously administer EpFA to test whether IL-10 secretion is rescued and

inflammation is reduced in DSS-treated Atg7 AD mice? Can the authors provide stronger support for a direct role of EPHX1?

This is a great suggestion and has been raised by all 3 reviewers. We are demonstrating a direct link between oxylipin levels and IL-10 secretion in three different ways:

First, we investigated the effects of different epoxy fatty acids on Il-10 production from macrophages *in vitro*. To address this, we used RAW264.7 macrophages which were pre-treated with epoxy fatty acids and then stimulated with LPS. The addition of epoxy fatty acid 11,12-EET increased *Il10* transcriptional levels and both 11,12-EET and 19,20-EDP led to secretion of IL-10 into the supernatant after 12h of culture (**NEW Figure 7A and 7B,** respectively).

Second, we inhibited cytochrome P450 in adipose tissue explants from water- or DSS-treated WT mice by adding the pan-cytochrome P450 inhibitor 1-ABT *ex vivo* (see cartoon **Fig 5I**). 1-ABT prevents the CYP450-mediated production of epoxy fatty (Su *et al.* (PMID: 9688677)). The addition of 1-ABT significantly inhibited the DSS-induced IL-10 secretion from adipose tissue explants from WT mice (**NEW Figure 7C**), indicating a crucial role of cytochrome P450 in the secretion of IL-10 from the adipose tissue during DSS-induced colitis.

Third, we measured the effect of EPHX enzymes on the production of IL-10 by using EPHX inhibitors. For this, we extracted adipose tissue explants from DSS-treated WT or *Atg7*^{Ad} mice and cultured them either in the absence or presence of the EPHX1 inhibitor NTPA and EXPH2 inhibitor TPPU. This *ex vivo* inhibition resulted in a recovery of IL-10 secretion from *Atg7*^{Ad} adipose tissue explants (**NEW Figure 7D**), suggesting that EPHX enzymes activity is responsible for the regulation of IL-10 secretion from autophagy-deficient adipose tissues.

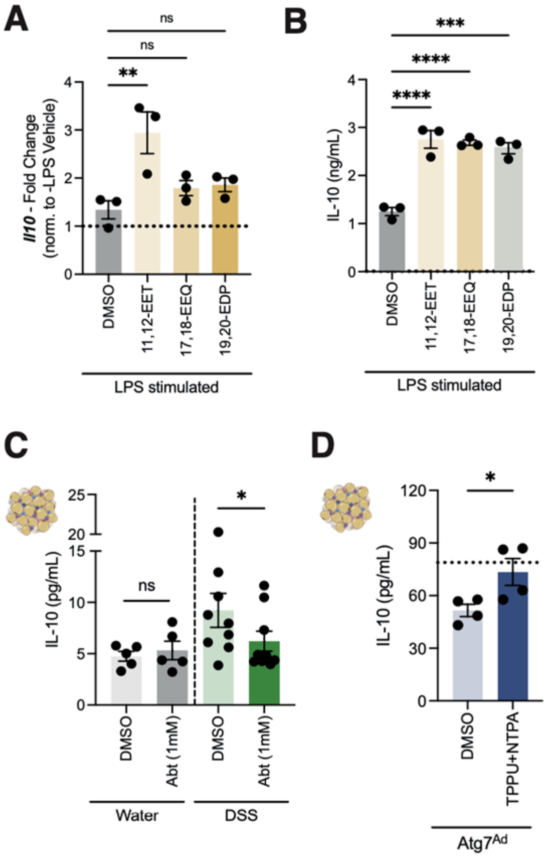

NEW Figure 7: Cytochrome P450-EPHX pathway regulates IL-10 secretion from autophagy-deficient adipose tissues upon DSS-induced intestinal inflammation.
(A) Quantification of *Il10* transcript levels in RAW264.7 upon stimulation with epoxy fatty acids (n = 3/group).
(B) Quantification of IL-10 protein levels in the supernatant of RAW264.7 upon stimulation with epoxy fatty acids (n = 3/group).
(C) Quantification of IL-10 protein levels in the supernatant of *ex vivo* cultured adipose tissues from water- or DSS-treated wild-type mice in the absence or presence of the cytochrome P450 inhibitor 1-ABT (n = 5-9/group).
(D) Quantification of IL-10 protein levels in the supernatant of *ex vivo* cultured adipose tissues from DSS-induced *Atg7*^{Ad} mice in the absence or presence of the EPHX1 inhibitor NTPA and EPHX2 inhibitor TPPU (n = 4/group).
Data are represented as mean ± s.e.m. (A,B) One-Way ANOVA. (C,D) Paired Student's t-test.

We included the following result section in

the new manuscript:

> "<u>*Cytochrome P450-EPHX pathway regulates IL-10 secretion from autophagy-deficient adipocytes during intestinal inflammation*</u>
> *To establish a more mechanistic link between the increased function of the cytochrome P450-EPHX pathway and IL-10 in adipose tissues, we first determined whether EpFA supplementation improves IL-10 production from macrophages in vitro. Pre-treatment of RAW264.7 macrophages with different EpFA increased Il10 transcript levels upon LPS stimulation (**Fig 7A**), which was further confirmed on protein level in the supernatant (**Fig 7B**). Cytochrome P450 enzymes are key for the production of EpFA. Inhibition of cytochrome P450 resulted in a marked reduction of IL-10 secretion from DSS-induced wild-type adipose tissues (**Fig 7C**), suggesting that cytochrome P450 is crucial for adipose tissue-derived IL-10 during intestinal inflammation. Lastly, blockade of EPHX1 and EPHX2 in* $Atg7^{Ad}$ *adipose tissue explants rescued IL-10 production (**Fig 7D**), establishing that EPHX enzyme activity can control IL-10 in autophagy-deficient adipose tissues. Collectively, this data indicates that EpFA and enzymes controlling their production and degradation can alter adipose tissue IL-10 levels during intestinal inflammation and this is dependent on autophagy."*

3. Although they show less IL-10 secretion from adipose tissues taken from DSS-treated Atg7 AD mice, it is unclear if this is specifically due to a decreased proportion of M2 macrophages that produce IL-10. It would be helpful for the authors to perform the flow cytometry experiment in Fig 7B with wild-type and Atg7AD mice side-by-side with appropriate markers.

We thank the reviewer for suggesting this experiment. We performed the flow cytometry experiment as done in the previous figure 7B using $Atg7^{Ad}$ mice and their littermate controls. As previously shown, DSS-induced colitis increased IL-10 staining in adipose tissue macrophages (as designated by CD11b+ F4/80^{high}) isolated from WT mice during DSS induced colitis (**NEW Figure 6C-D**). This increase was no longer observed in the $Atg7^{Ad}$ KO mice upon DSS induced colitis. We also stained for IL-10 in CD4+ T cells (CD11b- TCRb+ CD4+) and B cells (CD11b- CD19+), showing no changes in these cell populations (**NEW Figure 6E**).

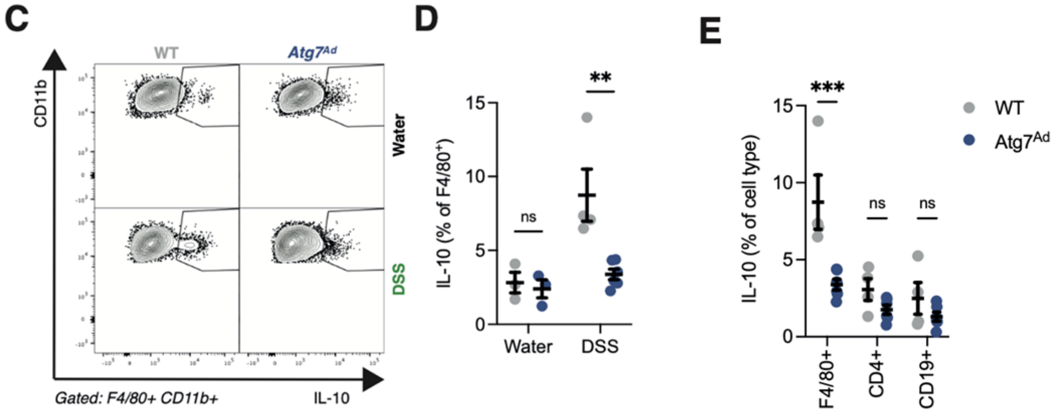

NEW Figure 6C-E: IL-10 production is downregulated in adipose tissue macrophages from $Atg7^{Ad}$ ***mice compared to WT mice upon DSS-induced colitis.*** (B) Representative flow cytometry blot for IL-10 in ATMs (gated as CD11b^{+} F4/80^{high}). (C) Quantification of flow cytometry results. (D) Frequency of IL-10^{+} cell in gonadal adipose tissue immune cells populations, including CD4+ T cells (CD11b^{-} TCRb^{+} CD4^{+}) and B cells (CD11b^{-} CD19^{+}), at day 7 upon DSS-induced colitis. Data pooled from two independent experiments.

In the supplementary Figure (now **Fig EV6**), we showed a slight reduction in CD206 and more pronounced reductions in CD36 (another M2 marker) in the adipocyte autophagy-deficient mice. Similarly, we found that in WT mice, there is an upregulation of CD36 upon DSS-induced colitis, while CD206 remains largely unchanged upon DSS colitis (**Reviewer Figure 8**). Roughly 85-95% of all detected macrophages keep the tissue-resident marker CD206. While the expression of CD206 remains largely unchanged, we found that ATMs increase secretion and production of IL-10 in the adipose tissue (see **now Figure 6A-D**). We believe the reduction of CD36 and CD206 expression observed in the $Atg7^{Ad}$ mouse model may rather be a reflection of a change in lipid composition and less likely a reflection of a complete polarization change. To better reflect this, we altered the text in the manuscript related to the expression of CD206 and CD36 on ATMs:

> *"EpFA can alter macrophage polarization and increase tissue-resident macrophage marker expression such as CD206 (Lopez-Vicario et al, 2015). In line with reduced EpFA levels in the adipose tissue, $Atg7^{Ad}$ ATMs had a slightly reduced CD206 expression (**Fig EV6B**) but remained the predominant type of macrophage in the tissue. In addition, expression of CD36, a lipid scavenging receptor which is commonly found on M2-type macrophages and induced on ATMs during lipolysis, was increased on the surface of ATMs in wild-type mice during DSS colitis. However, CD36 expression was not increased in $Atg7^{Ad}$ mice (**Fig EV6C**), indicating a distinct adaptation to different lipid availability in the adipose tissue."*

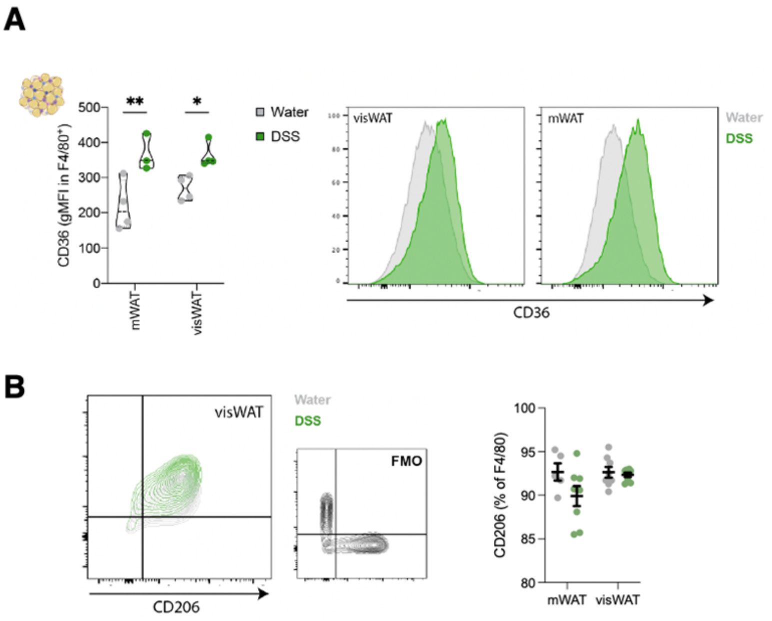

Reviewer Figure 8: Most macrophages in DSS-inflamed adipose tissues are expressing the tissue resident macrophage marker CD206. (A) Expression of CD36 was increased on ATMs in both mWAT and visWAT upon DSS-induced colitis. (B) Expression of tissue-resident marker CD206 ATMs upon DSS-induced colitis in WT mice.

Furthermore, the definition of macrophages from adipose tissues are difficult to be classified by distinct makers, which make them pro- or anti-inflammatory, but ATMs rather exist as a continuum (Caslin *et al.* (PMID: 32237081)). Characterizing IL-10 producing ATMs in DSS-induced colitis or human IBD conditions is an important future research avenue, to better understand their contribution in the pathologies.

Reviewer #3:

This study by Richter et al. reveals novel insights into the interaction between the inflamed gut and visceral adipose tissue and propose that adipose tissue autophagy play an important anti-inflammatory role during colitis. The manuscript demonstrates that adipocyte autophagy is activated during DSS induced colitis and that it has an anti-inflammatory effect limiting colitis. They found that the anti-inflammatory effect is unlikely to be due to increase release of FA from adipose tissue (using Atg7-/- mice). However, analysis of transcriptional changes mediated by loss of autophagy showed increase expression of genes involved in xenobiotic metabolism such as epoxide hydrolase and cytochrome P450 monooxygenase and the key transcription factor Nrf2. Oxylipin profiles in adipose tissue confirmed that adipocyte autophagy is required to increase conversion of oxylipins to more anti-inflammatory forms. Loss of adipocyte autophagy ultimately resulted in decreased number of CD206 anti-inflammatory macrophages and IL-10 secretion by visceral adipose tissue.

This study is important and highly relevant to human Crohn's disease which is often found to be associated with expansion and attachment of mesenteric adipose tissue to the inflamed intestine. While solidly constructed, the study does not demonstrate that the anti-inflammatory effect associated with autophagy is mediated by oxylipins and the translational relevance has not been explored. I have thus the following suggestions to improve the mechanistic insights and clinical relevance of the paper:

- Would it be possible to selectively block/activate the EPHX1 or EPHX2 enzymes to confirm involvement of oxylipins in anti-inflammatory effect of adipocyte autophagy? Or attempt to rescue loss of adipocyte autophagy with EpFA?
This is a great suggestion and has been raised by all 3 reviewers. We are demonstrating a direct link between oxylipin levels and IL-10 secretion in three different ways:

First, we investigated the effects of different epoxy fatty acids on Il-10 production from macrophages *in vitro*. To address this, we used RAW264.7 macrophages which were pre-treated with epoxy fatty acids and then stimulated with LPS. The addition of epoxy fatty acid 11,12-EET increased *Il10* transcriptional levels and both 11,12-EET and 19,20-EDP led to secretion of IL-10 into the supernatant after 12h of culture (**NEW Figure 7A and 7B,** respectively).

Second, we inhibited cytochrome P450 in adipose tissue explants from water- or DSS-treated WT mice by adding the pan-cytochrome P450 inhibitor 1-ABT *ex vivo* (see cartoon **Fig 5I**). 1-ABT prevents the CYP450-mediated production of epoxy fatty (Su *et al.* (PMID: 9688677)). The addition of 1-ABT significantly inhibited the DSS-induced IL-10 secretion from adipose tissue explants from WT mice (**NEW Figure 7C**), indicating a crucial role of cytochrome P450 in the secretion of IL-10 from the adipose tissue during DSS-induced colitis.

Third, we measured the effect of EPHX enzymes on the production of IL-10 by using EPHX inhibitors. For this, we extracted adipose tissue explants from DSS-treated WT or *Atg7*^{Ad} mice and cultured them either in the absence or presence of the EPHX1 inhibitor NTPA and EXPH2 inhibitor TPPU. This *ex vivo* inhibition resulted in a recovery of IL-10 secretion from *Atg7*^{Ad} adipose tissue explants (**NEW Figure 7D**), suggesting that EPHX enzymes activity is responsible for the regulation of IL-10 secretion from autophagy-deficient adipose tissues.

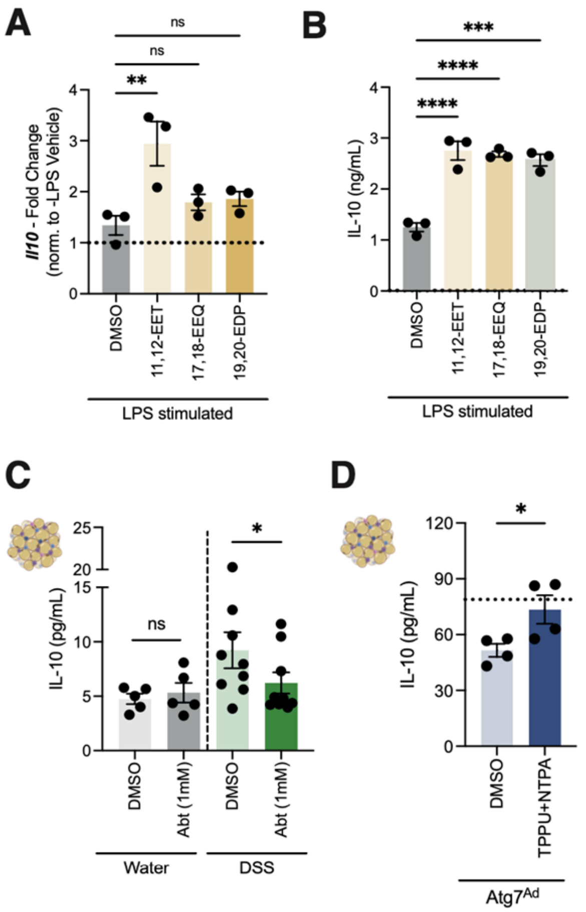

NEW Figure 7: Cytochrome P450-EPHX pathway regulates IL-10 secretion from autophagy-deficient adipose tissues upon DSS-induced intestinal inflammation.

(A) Quantification of *Il10* transcript levels in RAW264.7 upon stimulation with epoxy fatty acids (n = 3/group).

(B) Quantification of IL-10 protein levels in the supernatant of RAW264.7 upon stimulation with epoxy fatty acids (n = 3/group).

(C) Quantification of IL-10 protein levels in the supernatant of *ex vivo* cultured adipose tissues from water- or DSS-treated wild-type mice in the absence or presence of the cytochrome P450 inhibitor 1-ABT (n = 5-9/group).

(D) Quantification of IL-10 protein levels in the supernatant of *ex vivo* cultured adipose tissues from DSS-induced *Atg7*^{Ad} mice in the absence or presence of the EPHX1 inhibitor NTPA and EPHX2 inhibitor TPPU (n = 4/group).

Data are represented as mean ± s.e.m. (A,B) One-Way ANOVA. (C,D) Paired Student's t-test.

We included the following result section in the new manuscript:

> "*<u>Cytochrome P450-EPHX pathway regulates IL-10 secretion from autophagy-deficient adipocytes during intestinal inflammation</u>*
> *To establish a more mechanistic link between the increased function of the cytochrome P450-EPHX pathway and IL-10 in adipose tissues, we first determined whether EpFA supplementation improves IL-10 production from macrophages in vitro. Pre-treatment of RAW264.7 macrophages with different EpFA increased Il10 transcript levels upon LPS stimulation (**Fig 7A**), which was further confirmed on protein level in the supernatant (**Fig 7B**). Cytochrome P450 enzymes are key for the production of EpFA. Inhibition of cytochrome P450 resulted in a marked reduction of IL-10 secretion from DSS-induced wild-type adipose tissues (**Fig 7C**), suggesting that cytochrome P450 is crucial for adipose tissue-derived IL-10 during intestinal inflammation. Lastly, blockade of EPHX1 and EPHX2 in Atg7*^{Ad} *adipose tissue explants rescued IL-10 production (**Fig 7D**), establishing that EPHX enzyme activity can control IL-10 in autophagy-deficient adipose tissues. Collectively, this data indicates that EpFA and enzymes controlling their production and degradation can alter adipose tissue IL-10 levels during intestinal inflammation and this is dependent on autophagy."*

- Would it be possible to verify in human samples of mesenteric adipose tissue of Crohn's disease and relevant control that autophagy is activated in adipocytes?

We thank the reviewer for this comment, which we understand to be an important addition to the study in order to show its potential relevance to human disease. While we managed indeed to collect tissue samples from IBD patients over the revision period, we only obtained fat samples from 3 Crohn's Disease patients and one from a colon cancer patient as control

(under 60 years of age). We excluded any samples from patients aged 60 years and over since aging can reduce autophagic flux itself. From each patient, a piece of the inflamed creeping fat tissue (*CrF*) and an adjacent non-inflamed mesenteric tissue (*Ad. MAT*) piece (as control) were collected. As a further control, we collected mesenteric adipose tissue (MAT) near the caecum from a colorectal cancer patient. Tissue pieces for each location were either treated with 20mM NH_4Cl and 100µM Bafilomycin A1 to inhibit autophagic flux or incubated in DMSO for 4 hours at 37°C (same protocol as for the mouse tissue). Our results indicate that creeping fat tissues from Crohn's Disease patients have higher autophagic flux compared to their adjacent non-inflamed control (**New Fig 1J**). This is in line with our findings from the DSS mouse model.

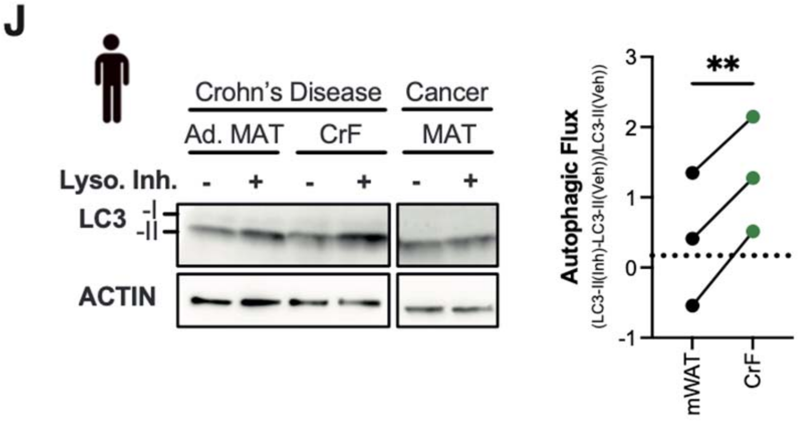

Revision Figure 9: Autophagic flux is increased in creeping fat tissue of Crohn's Disease patients. Measurement of autophagic flux in creeping fat (CrF) and non-inflamed adjacent mesenteric (Ad. MAT) using lysosomal inhibition using 20mM NH_4Cl and 100µM Bafilomycin A1. Similar to the mouse model, we found that autophagic flux was increased in the creeping fat (*CrF*) compared to non-inflamed adjacent mesenteric (*Ad. MAT*).

These findings indicate to us that there may be a connection between intestinal inflammation and adipocyte autophagy. Furthermore, we can speculate that the interactions observed in the mouse model of colitis may equally be present in the human condition. However, future studies will be required to better correlate changes in adipocyte autophagy during Crohn's disease with the increased IL-10 expression, previously observed in adipose tissue macrophages (Kredel et al. (PMID: 22543156)).

As above mentioned, we added the figure to the manuscript and added the following paragraph in the result section:

> *"Lastly, we wanted to assess whether the increase in autophagic flux also occurs in IBD. For this purpose, we collected creeping fat tissues and adjacent non-inflamed mesenteric adipose tissues from Crohn's disease patients. Interestingly, similar to the DSS-induced mice, we found an increased autophagic flux in the creeping fat compared to the same patient's adjacent non-inflamed mesenteric adipose tissue (Fig 1J)."*

- The results from RNAseq show that loss of Atg7 has no big effect on transcription. At the moment figures 4 and 5 feels like a diversion from the main purpose of the RNAseq experiment which was to identify transcriptional changes linked to loss of Atg7 explaining worse colitis outcome. The effect on the transactional regulation of genes involved in lipolysis are important but are apparently independent from Atg. Would it be possible to address this

finding earlier in the result part? I would suggest that results from figure 4A to C could go to supplementary.

We thank the author for this suggestion and restructured the paper. As suggested by the reviewer, we removed the analysis focussing on the transcriptional impact of DSS-induced colitis from the main figures and put it into the supplementary section.

In the revised version, we now discuss the increased lipolysis and autophagy in WT mice upon DSS induced colitis immediately in Figure 1. We then move onto autophagy's role in regulating lipolysis and show it is causing the observed effects. Finally, we present the transcriptomics that helped us to identify the Cyp450-EPHX pathway and demonstrate that it is responsible for altered IL-10 secretion from the adipose tissue.

Minor comments:

- In Figure 1:

Check statistic statement for B.

In this figure, we show that DSS-induced colitis results in a body weight loss as previously described (i.e. Kwon *et al.* (PMID: 33674694)) correlating with intestinal inflammation. We repeated the experiment and applied a two-way repeated measures ANOVA, since the same mice were weighed over time. The experiment indicates that since the start of the treatment, the DSS treated mice lose weight on day 6 (*p=0.0501*) and significantly lose more weight on day 7 (p=*0.0026)*.

For E, should you not use Mann-Whitney test instead of t-test?

In this previous figure 1E (now figure 1F) we used a t-test since we have seen in previous studies that the autophagic flux follows a normal distribution. We additionally tested our dataset for normality and found no indication of a non-normal distribution, with the caveat that 3-4 data points, in fact anything fewer than <20 data points, do not allow for a very reliable determination of underlying distribution.

To address the concerns of the reviewer further, we also applied a Mann-Whitney test for the requested graphs and found that the Mann-Whitney does not change the overall conclusions of the figure (*p(mWAT)=0.028* and *p(gWAT)=0.057*), indicating an increased autophagic flux in adipose tissues of DSS-treated mice.

For G, check normality.

We tested the dataset dealing with transcriptional changes in *Atg8* homologues in adipocytes and SVF (**NEW Figure 1H**) for normal distribution using D'Agostino & Pearson test and the Shapiro-Wilk test. The dataset passed normality testing using either of these tests.

- Could you discuss the possible impact of DSS on lipid absorption and potential drop in circulating FFA levels (and GAT loss).

We included in the discussion a section on the issues of intestinal disruption on lipid absorption and its impact on circulating FFA levels:

> *"Lipid uptake occurs across the intestine with highest levels of lipid absorption in the proximal small intestine. DSS leads to a disruption of the epithelial barrier towards the distal colon. However, some studies report that DSS can alter the morphology of the small intestine such as the jejunum, affecting its function and possibly lower dietary lipid absorption (Yazbeck et al, 2011). This could therefore alter uptake of dietary FFA, although FFA absorption in the context of DSS-induced colitis has not been conclusively determined. The observed decline in serum FFA levels may be connected to a reduced food intake during DSS-induced colitis (Vidal-Lletjos et al, 2019), their possible reduced absorption, and the depletion of lipid stores, such as gonadal adipose tissues. The induction of autophagy in the adipose tissue may help to maintain circulating FFA levels in addition to curb inflammation through signalling lipids."*

- Is mesenteric more affected by weight loss than Gonadal? Could you express at % loss compared to control or use different axis ?

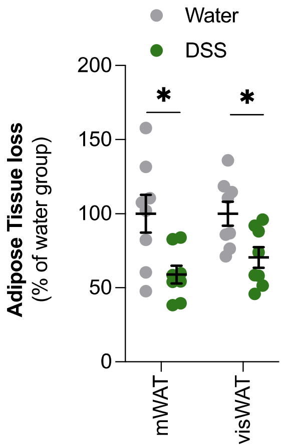

We thank the reviewer for the question. We expressed the adipose tissue loss (compared to the water-treated control group) in the **Reviewer Figure 10**. The loss of adipose tissues was comparable between the adipose tissues.

Reviewer Figure 10: Adipose wasting is equally affected in different adipose tissue depots. Changes in adipose tissue loss from Figure 1C was normalized to each water-treated control group, showing equal reduction in adipose tissue mass.

Figure 3: Provide repeat for 3F

As suggested by the reviewer, we repeated the experiment in two more independent experiments with 1-2/group/experiment. Our Western Blot results confirm the increase in pHSL and ATGL upon DSS-induced colitis (**Reviewer Figure 10**) and the data points were added to the figure (previously Fig. 3F, **now Figure 1E**).

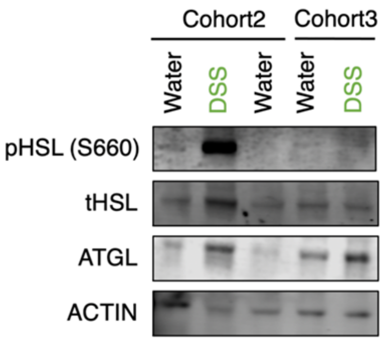

Reviewer Figure 10: Repeats of lipolysis key enzymes in gonadal adipose tissue at day 7 upon DSS-induced colitis. To repeat the experiment previously shown in Fig 3F, we performed two more independent small experiments showing that ATGL and pHSL are increased upon DSS-induced colitis. Data points were added to previous graph (**now Fig 1E**).

Figure 4E: Could you please show effect for each lipid species with stats, it is difficult to see effect on individual species.

All FFA species are now shown as bar graphs in the **NEW figure 3E**.

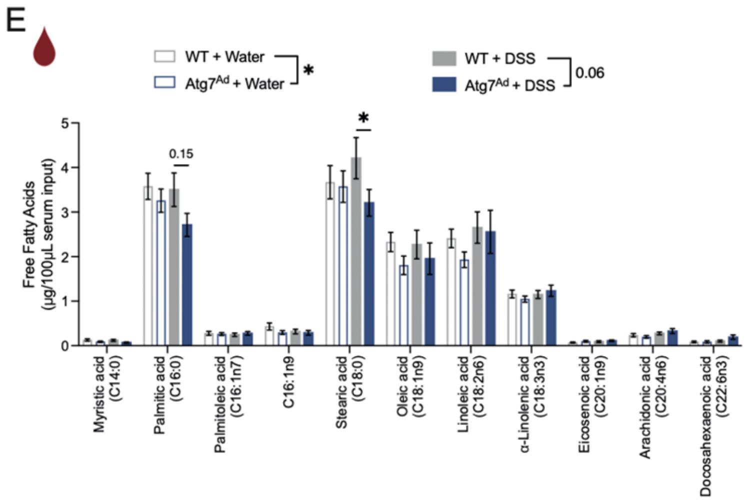

NEW Figure 3E: Free fatty acid species in serum of mice treated with water or DSS. Concentration of individual FFA species in serum in water-treated and DSS-treated mice as measured by FID-GC (n = 12-14/group).

Dear Katja,

We have now received re-review reports from two referees. As you will see, you have addressed their concerns satisfactorily. Before I can finally accept the manuscript though, there are some remaining editorial points which need to be addressed. In this regard would you please:

- remove text highlights
- rearrange figures to limit the number of EV figures to 5
- check the given email address for Sarah Wideman (sarah.wideman@imm.ox.ac.uk) which appears not to be correct
- include your Orcid ID
- include funding information for Project-ID 375876048 - TRR 241 and ERC AdG 670930 on our submission website. SFB-TRR 241 B01 is missing from the Acknowledgements section, but is referred to in the manuscript text
- rename the Conflict of Interest statement the Disclosure and Competing Interests statement
- remove the Authors' Contribution section from the manuscript text
- include callouts in the manuscript text for Figure 4C-4K (also please check whether the callouts on page 9 should be 4C-4K instead of mentioned 5C-5K)
- include an Appendix 1 file with a Table of Contents
- The EBI transcriptomic dataset access code 'E-MTAB-12498' is not accessible. Please check.
- We include a synopsis of the paper (see http://emboj.embopress.org/). Please provide me with a general summary statement and 3-5 bullet points that capture the key findings of the paper, along with a half-page summary figure.

EMBO Press is an editorially independent publishing platform for the development of EMBO scientific publications.

Best wishes,

William

William Teale, PhD
Editor
The EMBO Journal
w.teale@embojournal.org

Use the link below to submit your revision:

https://emboj.msubmit.net/cgi-bin/main.plex

Referee #2:

The manuscript now convincingly show the role of adipocyte autophagy in limiting gut inflammation through regulation of an oxylpin-IL-10 axis. I'm satisfied with the authors revision of the manuscript and would like to thank them for thoroughly and clearly answering all points raised by the reviewers.

Referee #3:

The authors have addressed my previous concerns. The new data are appreciated and the revised manuscript is much stronger.

All editorial and formatting issues were resolved by the authors.

2nd Revision - Editorial Decision 17th Jan 2023

Dear Katja,

I am pleased to inform you that your manuscript has been accepted for publication in the EMBO Journal.

Congratulations on making this really interesting connection. I am so glad everything worked out!

Please note that it is EMBO Journal policy for the transcript of the editorial process (containing referee reports and your response letter) to be published as an online supplement to each paper. If you do NOT want this, you will need to inform the Editorial Office via email immediately. More information is available here: https://www.embopress.org/page/journal/14602075/authorguide#transparentprocess

Your manuscript will be processed for publication in the journal by EMBO Press. Manuscripts in the PDF and electronic editions of The EMBO Journal will be copy edited, and you will be provided with page proofs prior to publication. Please note that supplementary information is not included in the proofs.

You will be contacted by Wiley Author Services to complete licensing and payment information. The required 'Page Charges Authorization Form' is available here: https://www.embopress.org/pb-assets/embo-site/tej_apc.pdf - please download and complete the form and return to embopressproduction@wiley.com

EMBO Press participates in many Publish and Read agreements that allow authors to publish Open Access with reduced/no publication charges. Check your eligibility: https://authorservices.wiley.com/author-resources/Journal-Authors/open-access/affiliation-policies-payments/index.html

Should you be planning a Press Release on your article, please get in contact with embojournal@wiley.com as early as possible, in order to coordinate publication and release dates.

If you have any questions, please do not hesitate to call or email the Editorial Office. Thank you for your contribution to The EMBO Journal.

Best wishes,

William

William Teale, PhD
Editor
The EMBO Journal
w.teale@embojournal.org

** Click here to be directed to your login page: https://emboj.msubmit.net

EMBO Press Author Checklist

Corresponding Author Name: Anna Katharina Simon	
Journal Submitted to: EMBO Journal	
Manuscript Number: EMBOJ-2022-112202	

USEFUL LINKS FOR COMPLETING THIS FORM
The EMBO Journal - Author Guidelines
EMBO Reports - Author Guidelines
Molecular Systems Biology - Author Guidelines
EMBO Molecular Medicine - Author Guidelines

Reporting Checklist for Life Science Articles (updated January
This checklist is adapted from Materials Design Analysis Reporting (MDAR) Checklist for Authors. MDAR establishes a minimum set of requirements in transparent reporting in the life sciences (see Statement of Task: 10.31222/osf.io/9sm4x). Please follow the journal's guidelines in preparing your
Please note that a copy of this checklist will be published alongside your article.

Abridged guidelines for figures
1. Data
The data shown in figures should satisfy the following conditions:
- the data were obtained and processed according to the field's best practice and are presented to reflect the results of the experiments in an accurate and unbiased manner.
- ideally, figure panels should include only measurements that are directly comparable to each other and obtained with the same assay.
- plots include clearly labeled error bars for independent experiments and sample sizes. Unless justified, error bars should not be shown for technical
- if n<5, the individual data points from each experiment should be plotted. Any statistical test employed should be justified.
- Source Data should be included to report the data underlying figures according to the guidelines set out in the authorship guidelines on Data

2. Captions
Each figure caption should contain the following information, for each panel where they are relevant:
- a specification of the experimental system investigated (eg cell line, species name).
- the assay(s) and method(s) used to carry out the reported observations and measurements.
- an explicit mention of the biological and chemical entity(ies) that are being measured.
- an explicit mention of the biological and chemical entity(ies) that are altered/varied/perturbed in a controlled manner.
- the exact sample size (n) for each experimental group/condition, given as a number, not a range;
- a description of the sample collection allowing the reader to understand whether the samples represent technical or biological replicates (including how many animals, litters, cultures, etc.).
- a statement of how many times the experiment shown was independently replicated in the laboratory.
- definitions of statistical methods and measures:
  - common tests, such as t-test (please specify whether paired vs. unpaired), simple χ2 tests, Wilcoxon and Mann-Whitney tests, can be unambiguously identified by name only, but more complex techniques should be described in the methods section;
  - are tests one-sided or two-sided?
  - are there adjustments for multiple comparisons?
  - exact statistical test results, e.g., P values = x but not P values < x;
  - definition of 'center values' as median or average;
  - definition of error bars as s.d. or s.e.m.

Please complete ALL of the questions below.
Select "Not Applicable" only when the requested information is not relevant for your study.

Materials

Newly Created Materials	Information included in the manuscript?	In which section is the information available? (Reagents and Tools Table, Materials and Methods, Figures, Data Availability Section)
New materials and reagents need to be available; do any restrictions apply?	Not Applicable	

Antibodies	Information included in the manuscript?	In which section is the information available? (Reagents and Tools Table, Materials and Methods, Figures, Data Availability Section)
For **antibodies** provide the following information:<br>- Commercial antibodies: RRID (if possible) or supplier name, catalogue number and or/clone number<br>- Non-commercial: RRID or citation	Yes	Materials and Methods

DNA and RNA sequences	Information included in the manuscript?	In which section is the information available? (Reagents and Tools Table, Materials and Methods, Figures, Data Availability Section)
**Short novel DNA or RNA including primers, probes**: provide the sequences.	Not Applicable	Taqman Probes are stated in Materials and Methods

Cell materials	Information included in the manuscript?	In which section is the information available? (Reagents and Tools Table, Materials and Methods, Figures, Data Availability Section)
**Cell lines:** Provide species information, strain. Provide accession number in repository **OR** supplier name, catalog number, clone number, and/**OR** RRID.	Not Applicable	
**Primary cultures:** Provide species, strain, sex of origin, genetic modification status.	Not Applicable	
Report if the cell lines were recently **authenticated** (e.g., by STR profiling) and tested for mycoplasma contamination.	Not Applicable	

Experimental animals	Information included in the manuscript?	In which section is the information available? (Reagents and Tools Table, Materials and Methods, Figures, Data Availability Section)
**Laboratory animals or Model organisms:** Provide species, strain, sex, age, genetic modification status. Provide accession number in repository **OR** supplier name, catalog number, clone number, **OR** RRID.	Yes	Materials and Methods
**Animal observed in or captured from the field:** Provide species, sex, and age where possible**.**	Not Applicable	
Please detail **housing and husbandry conditions**.	Yes	Materials and Methods

Plants and microbes	Information included in the manuscript?	In which section is the information available? (Reagents and Tools Table, Materials and Methods, Figures, Data Availability Section)
**Plants:** provide species and strain, ecotype and cultivar where relevant, unique accession number if available, and source (including location for collected wild specimens).	Not Applicable	
**Microbes:** provide species and strain, unique accession number if available, and source.	Not Applicable	

Human research participants	Information included in the manuscript?	In which section is the information available? (Reagents and Tools Table, Materials and Methods, Figures, Data Availability Section)
If collected and within the bounds of privacy constraints report on age, sex and gender or ethnicity for all study participants.	Yes	Materials and Methods

Core facilities	Information included in the manuscript?	In which section is the information available? (Reagents and Tools Table, Materials and Methods, Figures, Data Availability Section)
If your work benefited from core facilities, was their service mentioned in the acknowledgments section?	Yes	Acknowledgements

Design

Study protocol	Information included in the manuscript?	In which section is the information available? (Reagents and Tools Table, Materials and Methods, Figures, Data Availability Section)
If study protocol has been **pre-registered, provide DOI in the manuscript**. For clinical trials, provide the trial registration number **OR** cite DOI.	Not Applicable	
Report the **clinical trial registration number** (at ClinicalTrials.gov or equivalent), where applicable.	Not Applicable	

Laboratory protocol	Information included in the manuscript?	In which section is the information available? (Reagents and Tools Table, Materials and Methods, Figures, Data Availability Section)
Provide DOI OR other citation details if **external detailed step-by-step protocols** are available.	Yes	Adipose tissue protein extraction (An YA, Scherer PE (2020) Mouse Adipose Tissue Protein Extraction. DOI: 10.21769/BioProtoc.3631)

Experimental study design and statistics	Information included in the manuscript?	In which section is the information available? (Reagents and Tools Table, Materials and Methods, Figures, Data Availability Section)
Include a statement about **sample size** estimate even if no statistical methods were used.	Not Applicable	
Were any steps taken to minimize the effects of subjective bias when allocating animals/samples to treatment (e.g. **randomization procedure**)? If yes, have they been described?	Not Applicable	
Include a statement about **blinding** even if no blinding was done.	Yes	Histology samples were scored in a blinded manner.
Describe **inclusion/exclusion criteria** if samples or animals were excluded from the analysis. Were the criteria pre-established?<br><br>If sample or data points were omitted from analysis, report if this was due to attrition or intentional exclusion and provide justification.	Yes	Samples which were lost during experiments (i.e. errors during preparation) were excluded from final analysis. Further, outliers based on ROUT (Q=1%) were removed from analysis as stated in section "Statistical Analysis")
For every figure, are **statistical tests** justified as appropriate? Do the data meet the assumptions of the tests (e.g., normal distribution)? Describe any methods used to assess it. Is there an estimate of variation within each group of data? Is the variance similar between the groups that are being statistically compared?	Yes	Figures

Sample definition and in-laboratory replication	Information included in the manuscript?	In which section is the information available? (Reagents and Tools Table, Materials and Methods, Figures, Data Availability Section)
In the figure legends: state number of times the experiment was **replicated** in laboratory.	Yes	Figures, Materials and Methods
In the figure legends: define whether data describe **technical or biological replicates**.	Yes	Figures (all data points are biological data points)

Ethics

Ethics	Information included in the manuscript?	In which section is the information available? (Reagents and Tools Table, Materials and Methods, Figures, Data Availability Section)
Studies involving **human participants**: State details of **authority granting ethics approval** (IRB or equivalent committee(s), provide reference number for approval.	Yes	Materials and Methods
Studies involving **human participants**: Include a statement confirming that **informed consent** was obtained from all subjects and that the experiments conformed to the principles set out in the WMA Declaration of Helsinki and the Department of Health and Human Services Belmont Report.	Yes	Materials and Methods
Studies involving **human participants:** For publication of **patient photos**, include a statement confirming that consent to publish was obtained.	Not Applicable	
Studies involving experimental **animals**: State details of **authority granting ethics approva**l (IRB or equivalent committee(s), provide reference number for approval. Include a statement of compliance with ethical regulations.	Yes	Materials and Methods
Studies involving **specimen and field samples:** State if relevant **permits** obtained, provide details of authority approving study; if none were required, explain why.	Not Applicable	

Dual Use Research of Concern (DURC)	Information included in the manuscript?	In which section is the information available? (Reagents and Tools Table, Materials and Methods, Figures, Data Availability Section)
Could your study fall under dual use research restrictions? Please check biosecurity documents and list of **select agents and toxins** (CDC): https://www.selectagents.gov/sat/list.htm	Not Applicable	
If you used a select agent, is the security level of the lab appropriate and reported in the manuscript?	Not Applicable	
If a study is subject to dual use research of concern regulations, is the name of the **authority granting approval and reference number** for the regulatory approval provided in the manuscript?	Not Applicable	

Reporting

The MDAR framework recommends adoption of discipline-specific guidelines, established and endorsed through community initiatives. Journals have their own policy about requiring specific guidelines and recommendations to complement MDAR.

Adherence to community standards	Information included in the manuscript?	In which section is the information available? (Reagents and Tools Table, Materials and Methods, Figures, Data Availability Section)
State if relevant guidelines or checklists (e.g., **ICMJE, MIBBI, ARRIVE, PRISMA**) have been followed or provided.	Not Applicable	
For **tumor marker prognostic studies,** we recommend that you follow the **REMARK** reporting guidelines (see link list at top right). See author guidelines, under 'Reporting Guidelines'. Please confirm you have followed these guidelines.	Not Applicable	
For **phase II and III randomized controlled trials**, please refer to the **CONSORT** flow diagram (see link list at top right) and submit the CONSORT checklist (see link list at top right) with your submission. See author guidelines, under 'Reporting Guidelines'. Please confirm you have submitted this list.	Not Applicable	

Data Availability

Data availability	Information included in the manuscript?	In which section is the information available? (Reagents and Tools Table, Materials and Methods, Figures, Data Availability Section)
Have **primary datasets** been deposited according to the journal's guidelines (see 'Data Deposition' section) and the respective accession numbers provided in the Data Availability Section?	Yes	Materials and Methods, Data Availability
Were **human clinical and genomic datasets** deposited in a public access-controlled repository in accordance to ethical obligations to the patients and to the applicable consent agreement?	Not Applicable	
Are **computational models** that are central and integral to a study available without restrictions in a machine-readable form? Were the relevant accession numbers or links provided?	Not Applicable	
If publicly available data were reused, provide the respective **data citations in the reference list**.	Yes	Figures, References
